# Assessment of clinical and neuroimaging efficacy of treatment targeting tau pathology in mild cognitive impairment and mild to moderate Alzheimer’s disease with hydromethylthionine mesylate using external control data^✰^^✰✰^

**DOI:** 10.1016/j.tjpad.2026.100560

**Published:** 2026-04-17

**Authors:** Bjoern O Schelter, Helen Shiells, Serena Lo, Nafeesa Nazlee, Emily Evans, Peter Bentham, Serge Gauthier, Henrik Zetterberg, Gordon K Wilcock, Lutz Froelich, Alistair Burns, Emer MacSweeney, Clive Ballard, Jin-Tai Yu, Tay Siew Choon, Vahe Asvatourian, Natalia Muehlemann, Jan Priel, Karin Kook, Tenecia Sullivan, Diane Downie, Sonya Miller, Carol Pringle, John M․D Storey, Tom Baddeley, Charles R Harrington, Roger Staff, Anca-Larisa Sandu, Claire Hull, Richard Stefanacci, Claude M Wischik, Alzheimer's Disease Neuroimaging Initiative

**Affiliations:** a TauRx Therapeutics Ltd., Aberdeen AB24 5RP, UK; b Institute for Complex Systems and Mathematical Biology, University of Aberdeen, Aberdeen, UK; c Birmingham and Solihull Mental Health NHS Foundation Trust, Birmingham, UK; d Department of Neurology and Neurosurgery, McGill University, Montreal, Quebec, Canada; e Department of Psychiatry and Neurochemistry, Institute of Neuroscience & Physiology, Sahlgrenska Academy at the University of Gothenburg, Mölndal, Sweden; f Clinical Neurochemistry Laboratory, Sahlgrenska University Hospital, Mölndal, Sweden; g Department of Neurodegenerative Disease, UCL Institute of Neurology, London, United Kingdom; h UK Dementia Research Institute at UCL, London, United Kingdom; i Hong Kong Centre for Neurodegenerative Diseases, Clear Water Bay, Hong Kong, China; j Wisconsin Alzheimer’s Disease Research Centre, University of Wisconsin School of Medicine and Public Health, University of Wisconsin-Madison, Madison WI 53792, USA; k Geratology, University of Oxford, Oxford, UK; l Department of Geriatric Psychiatry, Medical Faculty Mannheim, Central Institute of Mental Health, Heidelberg University, Mannheim (SCU-B), Germany; m University of Manchester, Manchester, UK; n Re:Cognition Health London UK; o Clinical and Biomedical Sciences, Faculty of Health and Life Sciences, University of Exeter, Exeter, UK; p Department of Neurology, Huashan Hospital, Fudan University, Shanghai, China; q Cytel Inc. 675 Massachusetts Ave, Cambridge, MA 02139, USA; r Salamandra LLC, Bethesda, MD, USA; s Department of Chemistry, University of Aberdeen, Aberdeen AB24 3UE, UK; t Institute of Medical Sciences, University of Aberdeen, Aberdeen AB25 2ZD, UK; u Aberdeen Royal Infirmary NHS Grampian, Aberdeen Royal Infirmary, Foresterhill, Aberdeen AB25 2ZN, UK; v Aberdeen Biomedical Imaging Centre, School of Medicine, Medical Sciences and Nutrition, University of Aberdeen, Lilian Sutton Building, Foresterhill, Aberdeen AB25 2ZD, UK; w Thomas Jefferson University, Philadelphia, PA, USA

**Keywords:** Hydromethylthionine mesylate, Tau aggregation inhibitor, Alzheimer’s disease, Critical Path for Alzheimer’s Disease (CPAD)

## Abstract

**Background:**

Hydromethylthionine mesylate (HMTM) targets tau pathology and also has tau-independent symptomatic activity. A traditional randomised placebo-controlled trial (RCT) was precluded by loss of blinding due to urinary colouration and therapeutic activity at the minimum dose required to maintain blinding.

**Objective:**

To evaluate the efficacy of HMTM in participants with mild cognitive impairment (MCI) and mild to moderate dementia due to Alzheimer’s disease (AD).

**Methods:**

Because a traditional RCT was not feasible without loss of blinding, we compared HMTM 16 mg/day in TRx-237–039 with propensity score matched true placebo controls from the FDA-sponsored Critical Path for AD (CPAD) database with the same inclusion/exclusion criteria (protocol TRx-237–080). We also compared HMTM 16 mg/day with matched natural history controls from the Alzheimer’s Disease Neuroimaging Initiative (ADNI) and with a meta-analysis of placebo arms from trials in comparable populations in analyses specified prior to the 104-week database lock of TRx-237–039.

**Participants:**

Propensity score matching yielded 127 pairs (HMTM *n* = 127; CPAD placebo *n* = 127) in the CPAD comparison, and 189 pairs in the ADNI comparison. A total of 218 receiving HMTM 16 mg/day were compared with meta-analytic controls (*n* = 1805–8567).

**Intervention:**

HMTM 16mg/day

**Measurements:**

Primary outcomes in TRx-237–080 were change from baseline to 78 weeks in ADAS-Cog_13_ and whole brain volume (WBV). CDR-Sum of Boxes (CDR-SB) and CDR-Global were analysed at 104 weeks. ADAS-cog_11_ and WBV were analysed in ADNI comparisons, and ADAS-cog_11_, ADCS-ADL_23_, CDR-SB and WBV were analysed in meta-analytic comparisons.

**Results:**

Compared with matched CPAD placebo, HMTM 16 mg/day produced statistically significant differences in change on ADAS-Cog_13_ (*p* < 0.0001) and WBV at 78 (primary; *p* < 0.0001) and 104 weeks (*p* < 0.0001), and CDR-SB differed significantly overall (104-weeks; *p* < 0.001) and in MCI (*p* = 0.007). The odds of progressing to a more advanced CDR-Global stage were lower with HMTM (overall OR 0.31) and particularly in MCI (OR 0.15) versus CPAD placebo. Clinical and brain atrophy outcomes were similarly statistically significant in comparisons with ADNI case-matched natural history data and in meta-analytic comparisons.

**Conclusion:**

Comparisons of HMTM treatment with CPAD, ADNI, and meta-analytic controls provide evidence consistent with clinical benefit HMTM. It has the potential to offer an accessible oral treatment option which could be delivered with minimal patient/physician burden.

## 1 Introduction

Hydromethylthionine (HMT) is a potent oral tau aggregation inhibitor [[1], [2], [3]] which also has tau-independent symptomatic activity [4,5]. Hydromethylthionine mesylate (HMTM) is a stable crystalline form of HMT which permits direct delivery and absorption [1,3]. Methylthionium chloride (MTC) is an oxidised form of methylthionium which needs to be reduced in the stomach prior to absorption and delivery to the brain as HMT [1]. HMT, whether delivered as MTC [6] or HMTM [7], has been shown to reverse scopolamine-induced cognitive deficits in a standard mouse model for symptomatic activity.

In an accompanying paper [8], we reported the results of a Phase 3 trial TRx-237–039 in participants with a positive amyloid-PET scan at baseline and a clinical diagnosis of Alzheimer’s disease (AD) ranging in severity from mild cognitive impairment (MCI) to moderate dementia. This compared HMTM 16 mg/day with MTC 4 mg twice weekly on a varying schedule to maintain blinding with respect to the possibility of urinary colouration. Due to its atypical pharmacokinetics [9], the plasma concentration of HMT delivered by the low dose of MTC unexpectedly reached levels overlapping those required for activity in the scopolamine mouse model. This confounded the assessment of clinical efficacy of HMTM 16 mg/day at 52 weeks, but not treatment effects on progression of neurodegeneration and tau pathology measured by plasma biomarkers or grey matter atrophy over 52 – 104 weeks [8]. Statistically significant differences in cognitive decline according to initial randomisation emerged at 78 and 104 weeks in participants with MCI, with no clinical decline on cognitive or functional endpoints relative to baseline in those receiving HMTM 16 mg/day over 104 weeks [8].

Since TRx-237–039 did not have a clinically inert placebo control, we now report the results of further comparisons of HMTM 16 mg/day with external placebo control trial populations from the Critical Path in Alzheimer’s Disease (CPAD) database meeting the same inclusion/exclusion criteria and matched on important baseline characteristics using propensity score matching (PSM) under a new protocol and statistical analysis plan (SAP; TRx-237–080). In addition to the requirement for amyloid β-PET scan positivity to confirm that participants were on the AD-continuum [10], subjects were matched on age, sex, apolipoprotein E4 and smoking status, completion of high school education and disease severity as measured by both MMSE score and CDR-global score. We also report comparisons with PSM-matched natural history data from the Alzheimer’s Disease Neuroimaging Initiative (ADNI) database and with meta-analytic comparisons with publicly available data from placebo arms of trials in comparable populations. We provide evidence consistent with reduction in decline in patients treated with HMTM 16 mg/day relative to external control arm data, with clinically meaningful differences of 5–6 ADAS-cog_13_ units and 2–3 CDR-SB units relative to placebo data from external trial populations.

## 2 Methods

### 2.1 Study design and participants

The feeder studies for TRx-237–080 were from participants receiving HMTM 16 mg/day in TRx-237–039 and the studies available in the CPAD database from placebo arms of recent blinded trials in participants spanning MCI to moderate AD severity. A description of the rationale and the design of the feeder study TRx-237–039 (NCT03446001; EudraCT: 2017–003,558-17) has been published previously [11]. The study was conducted between 2017 and 2023 at 82 study sites located in Canada, the European Union, United Kingdom, and United States of America. Participants had to be less than 90 years of age and have a clinical diagnosis of probable AD or MCI due to AD [12,13]. The study was a double-blind, placebo-controlled, 52-week trial followed by a 52-week open-label extension in which all participants received HMTM 16 mg/day. All participants in TRx-237–039 provided written informed consent prior to enrolling in the study; legal representatives provided consent on behalf of patients with reduced decision-making capacity. Informants for the participants also provided consent for involvement. Similar consent requirements were met in external trials in comparable populations [listed in Supplementary Methods] and in the ADNI population. TRx-237–039 was conducted in accordance with the Declaration of Helsinki and the International council for Harmonisation Guidelines for Good Clinical Practice, and approval of the study protocol and all related documents was obtained from the appropriate Independent Ethics Committees and Institutional Review Boards for all study sites. An independent Data and Safety Monitoring Board was established for oversight of accruing safety information.

Because of the study design, data were available from participants randomised to receive HMTM 16 mg/day continuously over 104 weeks [8]. Clinical diagnosis as MCI-AD or mild to moderate dementia due to AD in TRx-237–039 was based on Investigator judgement according to the National Institute on Aging / Alzheimer’s Association (NIA/AA) 2011 criteria [12,13] who were required to confirm adherence to the diagnostic criteria listed in Data Verification Form. Participants with a clinical diagnosis of dementia and a CDR score of 2.0 or either 1.0 or 0.5 were classified as having either moderate or mild dementia respectively. Those with a clinical diagnosis of MCI had a CDR score of either 1.0 or 0.5.

### 2.2 Statistical methodology for clinical and WBV outcomes

**TRx-237–080.** The external matched placebo arm data used in the preparation of this paper come from the Critical Path Institute (C-Path) via the Critical Path for Alzheimer’s Disease (CPAD) database. C-Path is a nonprofit organization operating as an independent, public-private partnership with the U.S. FDA, created under the auspices of the FDA’s Critical Path Initiative program in 2005 [14]. Founded in 2008, the CPAD database contains, but is not limited to, demographic information, APOε4 genotype, concomitant medications, and cognitive scales (MMSE and ADAS-Cog). Limited AD biomarker data (biofluids, tau or amyloid positron emission tomography (PET), EEG data) are also available.

Comparisons with placebo subjects from the CPAD database were prespecified under a new protocol and SAP (TRx-237–080) prior to any access to the CPAD data. The CPAD database was used because it provides the most appropriate source of external data from clinical trials in comparable populations in which data were available in sufficient granularity to permit matching at the per-subject level. TRx-237–080 was finalised on 19-Nov-2024 prior to receipt of CPAD data on 26-Nov-2024. Selection of CPAD subjects and participants from TRx-237–039 who received 16 mg/day was based on meeting the same inclusion/exclusion criteria (including amyloid β-PET scan positivity) and matching using PSM for the covariates age, sex, *APO*ε*4* genotype and smoking status, completion of high school education and disease severity as measured by both MMSE score and CDR-global score. Although disclosure of dates for initiation and completion of external trial data was not permitted, the only trials in which amyloid β-PET scan positivity was included as a selection criterion date from 2015 to 2023 which provides a good overlap with the timing conduct of TRx-237–039 (2017 – 2023). The inclusion or exclusion of specific subjects from either TRx-237–039 or CPAD for key statistical comparisons could not be a known *a priori* because criteria for selection of matched subjects were defined algorithmically in the TRx-237–080 protocol. The variables used as covariates for matching were chosen because they have been shown to be predictors of progression in AD and the corresponding data were available from both sources. Subjects from the CPAD database who could be matched on inclusion/exclusion criteria and baseline covariates were classified as having either MCI or mild/moderate AD on basis of the diagnostic classification of the corresponding case from TRx-237–039. Optimal PSM was performed using the logit of the propensity score as a distance measure within 0.1 standard deviation and a 1:1 ratio. Amyloid β-PET scan positivity was required as an inclusion criterion for participants since this requirement is not generally used in trials in mild-to-moderate AD [12] and for these more severe subjects’ amyloid positivity was assumed if unavailable.

**Fig. 1:**
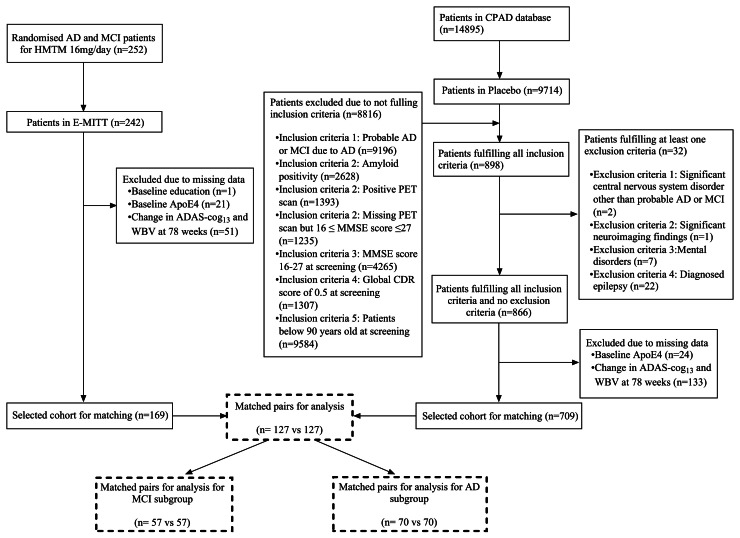
Inclusion/exclusion flowchart.

The matching as illustrated in the flowchart (Fig. 1) resulted in a true placebo arm comprising 127 subjects from the CPAD trial. Change from the baseline to 78 weeks in ADAS-cog_13_ and WBV were the key outcomes as dependent variables with treatment group as the independent variable using linear regression models and an ordinal model for change in CDR-global. MMSE was analysed at 52 and 104 weeks. CDR-SB and CDR-global were analysed only at 104 weeks (timepoints when data from both sources were available).

Since there were too few participants in the CPAD database who were not treated with acetylcholinesterase inhibitors or memantine to permit matching, this criterion was dropped for the primary analysis, but a modified version (exclusion of participants who started these drugs after randomisation and before their assessment at 104 weeks and matched according to prior history of taking these drugs) was applied and used in a sensitivity analysis, resulting in 106 pairs being available for analysis (Supplementary Table 6 and Supplementary Table 7).

Case selection requirements for the comparisons defined in TRx-237–080 included availability of data from both sources at 78 weeks. This approach, while ensuring strict comparability, only permitted comparisons in 112 cases for ADAS-cog_13_ and 99 cases for WBV change. To take account of data from all subjects meeting the required inclusion/exclusion criteria, sensitivity analyses were conducted comparing all participants randomised to receive HMTM 16 mg/day and subjects from the CPAD database. The extent of data missing at baseline or at Week 78 is summarised in Supplementary Table 1.

We undertook several quantitative bias analyses to assess the extent of bias in outcomes arising from missing covariate data or participant drop-out. Missing values were imputed with multiple imputation under various missing-at-random (MAR) and not-missing-at-random (NMAR) scenarios. For each imputed complete dataset, we used inverse propensity score weighting (IPW) to create a weighted pseudo-population in which measured baseline covariates were balanced between treatment groups with subsequent weighted linear regression to estimate the treatment effects and their standard errors. Rubin’s rules [15] were applied to pool results over all imputations. The methodology for calculating of IPW weights is provided in Supplementary methods. Propensity scores (PS) were estimated using three alternative modelling approaches: covariate balancing propensity score (CBPS), Bayesian additive regression trees (BART) and logistic regression (LR). Weighted linear regression was subsequently performed using weights from each of the three methods separately. In weighted linear regressions with ADAS-Cog_13_ change from baseline as outcome, we used treatment arm and baseline ADAS-Cog_13_ as covariates and where WBV change from baseline was the outcome we used treatment arm and baseline WBV as covariates. IPW using the three methods and weighted linear regression were performed with the R package WeightIt v1.5.1 [16]. Variance of the parameters was estimated using (the WeightIt default) method based on the asymptotically correct M-estimation as implemented in lm_weightit (vcov = "asympt") for logistic-regression-based and CBPS-based weights which accounts for the estimation of weights. For BART-based weights, asymptotically correct M-estimation is not available. For this we used the "HC0″ robust sandwich variance method as implemented in lm_weightit (vcov = "HC0") which treats the weights as fixed.

In these quantitative bias analyses, all 866 patients from CPAD and all randomized 252 patients from TRx-237–039 contributed to the estimates of the treatment effects on change in ADAS-cog_13_ and WBV over 78 weeks. Out of 252 HMTM patients, 105 had an MCI-AD diagnosis patients from TRx-237–039 and were analysed separately in a subgroup analysis. To investigate robustness of the results with respect to potential unmeasured confounding we calculated E-values.

**ADNI.** Data used in the preparation of this article were obtained from the ADNI database (adni.loni.usc.edu). The ADNI was launched in 2003 as a public-private partnership, led by Principal Investigator Michael W. Weiner, MD. The primary goal of ADNI has been to test whether serial magnetic resonance imaging (MRI), positron emission tomography (PET), other biological markers, and clinical and neuropsychological assessment can be combined to measure the progression of MCI and early AD. The ADNI population provides information on the natural history of disease progression in a real-world setting and was particularly useful because of its focus on collection of neuroimaging data which could be compared for grey matter atrophy at the voxel level.

ADNI participants who could be matched by PSM with TRx-237–039 participants were compared at 52-weeks as the primary timepoint and participants receiving HMTM 16 mg/day were compared as a subgroup. Subjects were matched on age, sex, smoking history, education, *APOE* genotype, non-use of AChEIs or memantine and baseline MMSE. There were too few ADNI subjects with a positive amyloid β-PET scan to permit application of this criterion. There were 431 TRx-237–039 trial participants and 322 ADNI cohort patients available for matching (Supplementary Figure 1). Matching was performed using the Nearest Neighbour PSM method with a calliper of 0.10–0.20 (depending on resulting sample sizes) of the standard deviation of the estimated logit and 1:1 matching without replacement. There were 189 matched pairs available for comparison with TRx-237–039 of whom a subset of 75 pairs was available for comparison of HMTM 16 mg/day. Methodological details for comparative analyses of grey matter atrophy using statistical parametric mapping (SPM) are provided in Supplementary methods.

**Meta-analytic controls.** All available participants in TRx-237–039 receiving HMTM 16 mg/day over 52 and 78 weeks were compared separately with meta-analytic controls available from a systematic literature review of published trials including also PubMed, the Cochrane Library and clinicaltrials.gov to identify full-text publications in English reporting double-blind, placebo-controlled, randomized clinical trial in patients with probable AD. As the MMSE distribution at baseline in participants with an MCI-AD clinical diagnosis in TRx-237–039 ranged from 16 – 27, and the criteria for MCI in clinical trials vary, we used data from published trials described as being in MCI/early AD populations for comparisons with the TRx-237–039 MCI population. Trials described as being in participants with mild-to-moderate AD were included as such. Measures of change in ADAS-cog, ADCS-ADL or WBV over at least 52 weeks were required. The meta-analytic comparisons included 59 publications describing 65 clinical trials. Studies excluded typically did not report key baseline characteristic (e.g., MMSE), did not report any measure of variance of change (e.g., standard deviation, standard error of the mean or confidence intervals), or reported only a sub-group analysis of a randomized clinical trial. Simple linear scaling based on maximum available score was used to generate common ADAS-cog_11_ and ADCS-ADL_23_ scores for trials using ADAS-cog and ADCS-ADL variants. Availability of a positive amyloid β-PET scan was included as a selection criterion only for trials described as being in MCI/early AD populations. Of the trials in mild-to-moderate AD listed in Supplementary methods, only 2 were found to include a baseline amyloid β-PET scan. Meta-analysis was performed using the R programming language (Version 4.3.1 or higher) utilizing the “rma” function from the “metafor” package, fitting a random-effects model using a restricted maximum likelihood estimator. Between study heterogeneity was assessed using the I² statistic. Assuming unequal variances, a two-sided independent samples *t*-test was used to assess the significance of differences between pooled means from the meta-analysis and the TRx-237–039 study. Corresponding p-values and 95% confidence intervals (CI) were calculated to quantify the uncertainty around the mean difference, and a p-value <0.05 was considered statistically significant. Meta-analytic means and standard deviations calculated separately from trials in MCI and mild-to-moderate AD populations were weighted to approximate the distribution in Study TRx-237–039 to provide overall comparisons. The number of studies and placebo subjects in these analyses are listed in the Supplementary methods.

### 2.3 MRI imaging

Sites included in TRx-237–039 were required to complete an imaging technical evaluation questionnaire to evaluate their technical and personnel capabilities, including machine description, availability of phantoms, onsite availability of an MRI-specific technologist, site experience in evaluating brain MRI, experience in AD and other dementia trials, etc. After site qualification for quality and standardisation, structural T1-weighted images were acquired using three-dimensional magnetisation-prepared rapid gradient echo or equivalent sequences to obtain 3-D T1 images of the brain at baseline and follow-up. Sequence parameters were selected to match the Alzheimer’s Disease Neuroimaging Initiative (ADNI) acquisition protocol (https://adni.loni.usc.edu/methods/mri-tool/mri-analysis/). All data were centrally collected, quality-controlled, and processed by an imaging core lab (Image and Data Archive (IDA), Laboratory of Neuro Imaging, University of Southern California) who performed quality checks to ensure that the appropriate sequences were acquired and that images were in the correct format and orientation. The processing accounted for any variation in total intracranial volume caused by machine drift over time. Upon completion of the study, the data were transferred to the University of Aberdeen for statistical parametric processing and analysis. The 3-D T1 brain images were processed using the Computational Anatomy Toolbox (CAT12: https://doi.org/10.1101/2022.06.11.495736) longitudinal integrated pipeline for voxel brain morphometry (VBM: https://doi.org/10.1006/nimg.2000.0582). The CAT12 pipeline includes various quality control steps and covers the entire analysis workflow, from longitudinal data processing to statistical analysis.

## 3 Results

### 3.1 TRx-237–080

We undertook a comparison of participants receiving HMTM 16 mg/day in TRx-237–039 who could be matched to a clinical trial placebo population identified from the CPAD database under a new protocol and SAP (Supplementary Methods). Of 9714 AD cases with placebo data in CPAD, 866 met the TRx-237–039 inclusion/exclusion criteria. Of these 709 had primary endpoint data to 78 weeks (Fig. 1). There were 169 participants in TRx-237–039 receiving HMTM 16 mg/day who had data available at 78 weeks. Their baseline characteristics prior to matching are shown in Table 1. Of these, 112 could be matched for comparison of ADAS-cog_13_ change to 78 weeks with baseline characteristics and standardised mean differences (SMDs) shown in Supplementary Table 2. There were 99 cases which could be matched for WBV change, with baseline characteristics and SMDs shown in Supplementary Table 2. As can be seen, the populations were balanced for all covariates considered with mean logit propensity scores −1.2 (SD 1.2) for ADAS-cog_13_ matching and −1.1 (1.2) for WBV matching. The baseline characteristics and SMDs for matched cases split by diagnosis as MCI (*N* = 49 each for ADAS-cog_13_, mean logit propensity score −1.3, SD 1.1; *N* = 44, each for WBV, mean logit propensity score −1.3, SD 1.1) or mild/moderate AD (*N* = 63 each for ADAS-cog_13_, mean logit propensity score −1.1, SD 1.2; *N* = 55 each for WBV, mean logit propensity score −0.9, SD 1.3 each for WBV), shown Supplementary Tables 3 and 4.

**Table 1:** Demographic and baseline clinical characteristics before and after matching TRx-237–039 and CPAD.

	Pre-matching		Post-matching	
Characteristic	CPAD Placebo	TRx-237–039 HMTM 16mg/day	CPAD Placebo	TRx-237–039 HMTM 16 mg/day
	*N* = 709*^1^*	*N* = 169*^1^*	*N* = 127^1^	*N* = 127^1^
Age (years)	73 (68, 79)	73 (66, 78)	71 (66, 78)	73 (66, 79)
Sex				
F	431 (61%)	109 (64%)	87 (69%)	89 (70%)
M	278 (39%)	60 (36%)	40 (31%)	38 (30%)
*APOE4*	474 (67%)	77 (46%)	54 (43%)	63 (50%)
Post-secondary Education	422 (60%)	73 (43%)	59 (46%)	60 (47%)
Smoking	35 (4.9%)	71 (42%)	30 (24%)	31 (24%)
Baseline CDR				
Mild impairment (1)	228 (32%)	70 (41%)	54 (43%)	51 (40%)
Moderate impairment (2)	11 (1.6%)	8 (4.7%)	3 (2.4%)	5 (3.9%)
Questionable impairment (0·5)	470 (66%)	91 (54%)	70 (55%)	71 (56%)
Baseline MMSE				
Mild	492 (69%)	79 (47%)	71 (56%)	63 (50%)
Moderate	103 (15%)	57 (34%)	33 (26%)	39 (31%)
Very mild	114 (16%)	33 (20%)	23 (18%)	25 (20%)
Baseline MMSE	23 (21, 25)	22 (19, 25)	22 (20, 25)	22 (19, 24)
Baseline ADAS-Cog_13_	29 (24, 34)	27 (20, 33)	29 (25, 36)	27 (20, 34)
Baseline ADAS-Cog_11_	18 (14, 22)	17 (13, 23)	18 (14, 24)	17 (13, 23)
Baseline WBV (cm^3^)	965.39 (900·25, 1035·29)	974.52 (912·14, 1048·68)	951.34 (886·16, 1037·06)	971.18 (911·72, 1041·80)
Logit Propensity Score	−2.1 (1.0)	−0.5 (1.5)	−1.17 (−2·09, −0·28)	−1.17 (−2·09, −0·25)

**Fig. 2:**
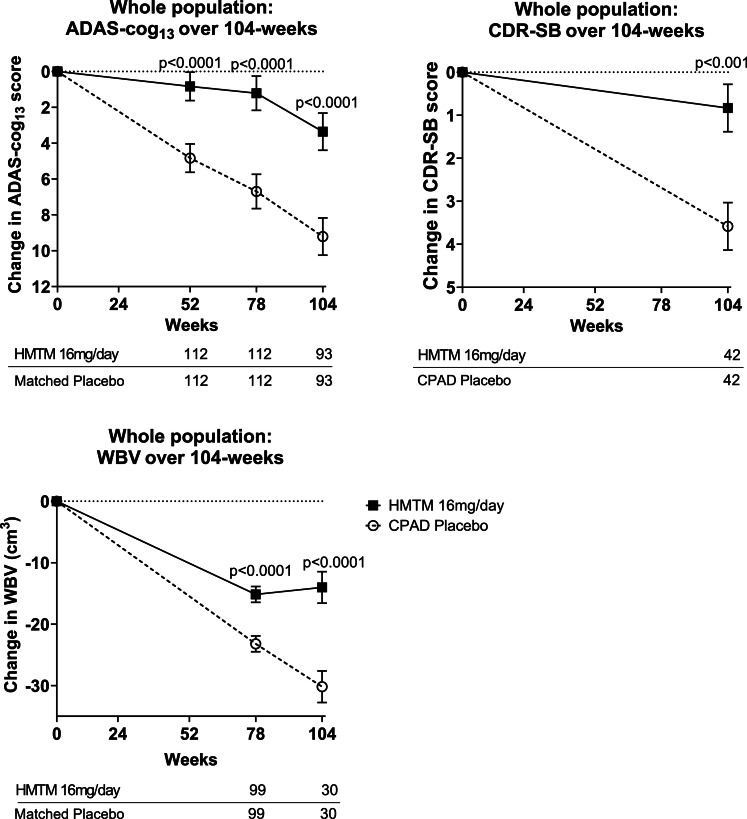
Change from baseline in ADAS-cog_13_, CDR-SB and WBV over 104-weeks in HMTM 16mg/day versus matched CPAD placebo group. Error bars denote SEM.

The principal outcome results for change in ADAS-cog_13_ and change in WBV are presented in Fig. 2 and Table 2**.** The differences on change in ADAS-cog_13_ and WBV between the HMTM 16 mg/day group and the matched placebo group were highly significant at 78 weeks, and also at 52 (ADAS-cog_13_ only) and 104 weeks. Change on CDR-SB was significant at 104 weeks; MMSE was directionally consistent at 52 and 104 weeks but not significant. Subgroup analyses indicated benefits predominantly in MCI, with significant effects on ADAS-cog_13_ and WBV at 78 and 104 weeks, CDR-SB at 104 weeks, and MMSE at 52-weeks, whereas in mild-to-moderate AD significance was limited to ADAS-cog_13_ and WBV at 104 weeks (Table 2)**.**

**Table 2:** Primary and secondary outcomes at 52, 78 and 104 weeks. Coprimary outcomes are indicated in bold.

Outcomes	Time Point	No. of Participants Evaluated	TRx-237–039 HMTM 16mg/day	Matched Placebo	TRx-237–039 HMTM 16mg/day vs Matched Placebo
	(weeks)	N	Mean ± SE	Mean ± SE	p-value (2-sided)
**Whole Population**					
ADAS-cog_13_	52	112	0·831 ± 0·781	4·830 ± 0·781	<0·0001
	**78**	**112**	**1·215 ± 0·951**	**6·696 ± 0·951**	**<0·0001**
	104	93	3·359 ± 1·033	9·215 ± 1·033	<0·0001
WBV (cm^3^)	**78**	**99**	**−15·18 ± 1·30**	**−23·42 ± 1·30**	**<0·0001**
	104	30	−14·02 ± 2·57	−30·20 ± 2·57	<0·0001
CDR-SB	104	42	0·763 ± 0·807	4·053 ± 0·807	<0·001
MMSE	52	121	−1·537 ± 0·390	−2·240 ± 0·390	0·204
	104	98	−3·653 ± 0·580	−4·408 ± 0·580	0·359
**MCI-AD**					
ADAS-cog_13_	52	49	−1.62 ± 1.08	5.39 ± 1.08	<0.001
	78	49	−1.28 ± 1.39	8.53 ± 1.39	<0.001
	104	38	2.88 ± 1.46	8.82 ± 1.46	0·005
WBV (cm^3^)	78	44	−11.34 ± 1.81	−24.81 ± 1.81	<0.001
	104	13	−7.50 ± 3.95	−32.89 ± 3.95	<0.001
CDR-SB	104	19	0.76 ± 0.81	4.05 ± 0.81	0.007
MMSE	52	52	−0.37 ± 0.55	−2.31 ± 0.55	0.014
	104	39	−1.85 ± 0.84	−3.77 ± 0.84	0.111
**AD**					
ADAS-cog_13_	52	63	2.74 ± 1.08	4.40 ± 1.08	0.280
	78	63	3.16 ± 1.27	5.27 ± 1.27	0.241
	104	55	3.69 ± 1.44	9.49 ± 1.44	0.005
WBV (cm^3^)	78	55	−18.24 ± 1.79	−22.31 ± 1.79	0.110
	104	17	−19.01 ± 3.17	−28.13 ± 3.17	0.050
CDR-SB	104	23	0.89 ± 0.76	3.20 ± 0.76	0.038
MMSE	52	69	−2.42 ± 0.54	−2.19 ± 0.54	0.760
	104	59	−4.85 ± 0.77	−4.83 ± 0.77	0.988

For participants receiving HMTM 16 mg/day, the odds ratio for progression to a more advanced CDR global stage was reduced to 0·31 (*p* = 0.005) overall and 0·15 (*p* = 0.006) in MCI/early AD relative to matched placebo (Supplementary Table 5). The treatment difference did not reach significance in mild/moderate AD. Overall, 71·4% (30/42) receiving HMTM 16 mg/day had the same or better CDR-global rating after 2 years, compared with 52·4% (22/42) for placebo.

#### 3.1.1 Sensitivity analyses – co-medication status with AChEIs or memantine

A sensitivity analysis taking account of non-use of AChEIs or memantine was undertaken to determine whether the results could be accounted for by differences in co-medication status. This reduced the number available for matching to 106 pairs (Supplementary Table 6). These treatments were used in 82% of CPAD subjects before matching and 50% after matching. The proportion for prior use in the HMTM 16 mg/day population was 34% before matching and 48% after matching. This restriction made little difference to change in ADAS-cog_13_ at 52, 78 or 104 weeks and change in WBV at 78 weeks (Supplementary Table 7). The results were similar in further sensitivity analyses in which participants were split according to baseline clinical diagnosis as MCI or mild-to-moderate AD. In participants with MCI, change in ADAS-cog_13_ was significant at 52, 78 and 104 weeks, as was change in WBV at 78 weeks. In participants with mild-to-moderate AD, change in ADAS-cog_13_ and WBV were significant only at 104 weeks.

#### 3.1.2 Sensitivity analyses – missing outcome data

There were 252 participants in TRx-237–039 randomised to HMTM 16 mg/day who received at least one post-baseline dose (ITT population). The extent of ADAS-cog_13_ data missing at baseline for measured covariates are summarised in Supplementary Table 1. *APO*ε*4* data were missing for participants receiving HMTM 16 mg/day in 27 cases (10.7%) overall and in 8 (7.6%) for participants with MCI. Post-secondary education status and baseline WBV were missing in 1 case overall (0.40% each), and none were missing in MCI. Baseline data missing from the CPAD database included ADAS-cog_13_ (51, 5.9%), WBV (28, 3.2%) and *APO*ε*4* (24, 2.8%). Data missing from the HMTM 16 mg/day overall and MCI populations at weeks 13, 26, 39, 52 and 78 are summarised in Supplementary Table 1 for ADAS-cog_13_ and at weeks 26, 39, 52 and 78 in for WBV. There were 75 (29.8%) cases overall and 29 (27.6%) with MCI who had ADAS-cog_13_ data missing at 78 weeks, and 89 (35.3%) overall and 34 (32.4%) with WBV data missing at 78 weeks. Standardised mean differences and logit propensity scores in measured covariates in the matched populations are provided for ADAS-cog_13_ and for WBV (Supplementary Table 2), for MCI matched populations (Supplementary Table 3) and for matched mild/moderate AD populations (Supplementary Table 4).

Weighted population comparisons are provided for each of the imputation approaches using CBPS, BART and LR under the MAR assumption in Supplementary Figure 2 for ADAS-cog_13_ in the whole population, and the NMAR assumption for the whole population for ADAS-cog_13_ (Supplementary Figure 3). The corresponding figures are provided for WBV under MAR in the whole population Supplementary Figure 4. For the NMAR assumption, the corresponding figures are Supplementary Figure 5 for the whole population. As can be seen from these comparisons, the weighting with best covariate balance relative to HMTM data was provided by CBPS. The BART and LR results are consistent with the CBPS results.

The first imputation approach (denoted copy-increments-in-reference, CIR) to account for missing data assumed that participants receiving HMTM retained treatment benefit up to the point of withdrawal and had their subsequent trajectory determined by the corresponding CPAD control. A second assumption (denoted-copy reference, CR) tested was that HMTM benefit up to the point of withdrawal was treated as though it had been achieved within the control arm, such that if the HMTM effect was above the control arm mean, the positive effect fed through to a degree determined by the correlation pattern in the control arm. The third assumption (denoted jump-to-reference, JR) tested was for withdrawn subjects to follow the CPAD trajectory from baseline onwards. These three assumptions were applied to both change in ADAS-cog_13_ and change in WBV under the CBPS methodology (Supplementary Table 8).

As can be seen in Supplementary Table 8, all estimated treatment effects for ADAS-cog_13_ were numerically lower than the MAR scenario (−5.24 units, 95% CI −7.33: −3.16, *p* < 0.0001), with CIR (−4.02 units, 95% CI −6.08: −1.95, *p* = 0.0001) and CR (−3.99 units, 95% CI −6.06:−1.93, *p* = 0.0002) producing comparable estimates, and the JR approach producing the smallest estimated treatment effect (−3.19 units, 95% CI −5.31:−1.07, *p* = 0.0032). All therefore differed significantly from the no-effect level. The same was true for WBV change (MAR: 6.61 cm^3^, 95% CI 4.05: 9.17, *p* < 0.0001; JR: 4.93 cm^3^, 95% CI 2.27:7.60, *p* = 0.0003). The estimated effects in MCI (Supplementary Table 8) were numerically larger for both ADAS-cog_13_ (MAR: −8.97 units, 95% CI −12.34: −5.60, *p* < 0.0001; CIR: −7.36 units, 95% CI −10.89: −3.83, *p* < 0.0001; CR-7.27 units, 95% CI −10.81: −3.73, *p* = 0.0001; JR: −5.95 units, 95% CI −9.77: −2.13, *p* = 0.0023) and WBV (MAR: 11.26 cm^3^, 95% CI 7.56: 14.95, *p* < 0.0001; JR: 8.07cm^3^, 95% CI 3.94:12.21, *p* = 0.0001). For WBV, only two imputations (JR and CR) were carried out.

To assess the influence of unmeasured confounders in the treatment effect estimation, we calculated the E-value (Supplementary Table 9) as described in Supplementary methods. We selected the treatment effect estimates from the MAR analysis, the more conservative CR imputation and the most conservative JR imputation. For each, we selected the least favourable treatment estimates based on their magnitudes from the three IPW methods and calculated the corresponding E-values. For ADAS-cog_13_ (Supplementary Table 9), we used a residual standard deviation of 9.002 (based on linear regression with all baseline covariates). An unmeasured confounder would need to be approximately twice as common in the HMTM arm compared to the CPAD cohort and independently associated with a two-fold increase in the risk of a worse outcome to fully explain away the observed treatment effect at least −3.192 ADAS-cog_13_ units. Similarly, using a standard deviation of 12.524, unmeasured confounders would need to be both twice as frequent in the HMTM arm and independently associated with a two-fold increase in the risk of a worse outcome to fully explain away the observed treatment difference on WBV change of at least 4.7 cm^3^. Similarly in MCI, assuming a residual standard deviation of 8.775 ADAS-cog_13_ units, unmeasured confounders would need to be approximately 3-fold more common in the HMTM cohort and be associated with a 3-fold risk of worse outcomes to explain a treatment effect of at least −5.946 units. For WBV change over 78 weeks in MCI, using a residual standard deviation of 12.124 explaining away a treatment effect of at least 7.922 cm^3^ would require the unmeasured confounders to be 3-time more likely in the HMTM arm independently associated with a three-fold increase in the risk of a worse outcome. In all cases, weaker unmeasured confounders would not be able to explain away the measured effects, and an association of such strength would have been known previously and would have been included as measured covariates.

### 3.2 ADNI comparison

**Fig. 3:**
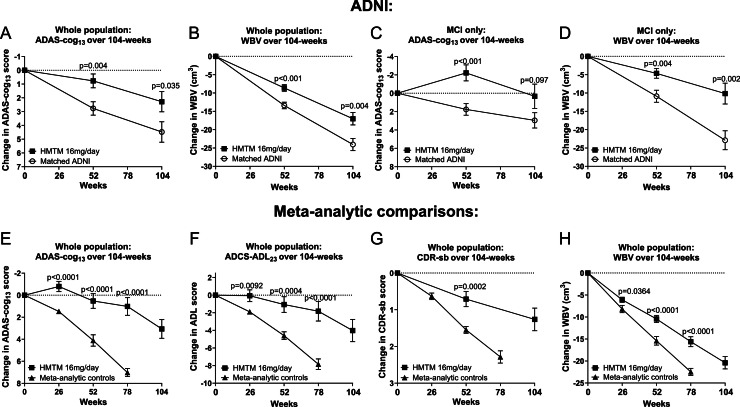
A-D: Comparisons of matched TRx-237–039 and ADNI Populations at 52 and 104-weeks in MCI & mild to moderate dementia due to AD and the MCI subpopulation. E-H: Comparison of HMTM 16 mg/day or MTC 4 mg twice weekly with meta-analysis of placebo arms from trials in comparable populations over 78-weeks. Error bars denote SEM.

A similar PS-matched analysis to that provided in TRx-237–080 was undertaken to compare with participants available from the ADNI database in 189 matched pairs. Baseline demographic and clinical characteristics before and after matching are provided in Supplementary Figure 1 and Supplementary Table 10. The differences for the SAP-specified outcomes (change in ADAS-cog_11_ and WBV) were statistically significant at 52 and 104 weeks (Supplementary Table 11; Fig. 3). Comparisons of the subpopulation receiving HMTM 16 mg/day were significant at 52 and 104-weeks for WBV, but only at 104-weeks for ADAS-cog_13._ Comparative analysis of grey matter atrophy for matched participants receiving HMTM (16 mg/day and 8 mg/day pooled) showed statistically significant reduction in hippocampus, parahippocampal gyrus, temporal lobes and parts of medial frontal lobe including cingulate gyrus (Supplementary Figure 6).

### 3.3 Meta-analytic comparisons

Since strict PSM matching necessarily restricted the number of participants who could be compared with external controls, meta-analytic comparisons were undertaken in which all participants receiving HMTM 16 mg/day were compared with placebo participants drawn from 20 to 32 external trial studies (totalling 5116–7914 subjects at 52 and 78 weeks) to permit greater generalisability. Despite the TRx-237–039 populations being significantly more impaired cognitively at baseline by 1.2–2.2 MMSE units (p-values 0·0223 to <0·0001 depending on data availability for each outcome), participants receiving HMTM 16 mg/day declined significantly less on clinical (ADAS-cog_11_ and ADCS-ADL_23_, *p* < 0.0001 for both) and whole brain volume loss (*p* < 0.0001) outcomes than has been reported in placebo arms from clinical trials in comparable populations (Fig. 3, Supplementary Table 12). A more stringent meta-analysis that considered only trials reporting identical endpoint versions yielded similarly significant advantages: statistically significant differences in decline on ADAS-cog_13_ (3397 subjects; 52 and 104 weeks; *p* = 0.0004 to <0.0001) and reduced brain atrophy (1133–1969 subjects) at 52 and 104 weeks (*p* = 0.38 to <0.0001; Fig. 3, Supplementary Table 13).

### 3.4 Comparative summary of external control arm results

**Fig. 4:**
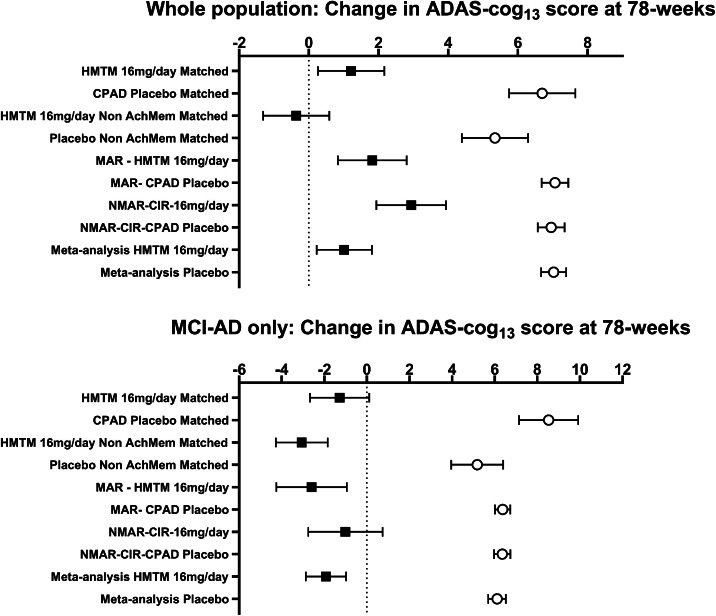
Forest plots of treatment effects in ADAS-cog_13_ for HMTM 16 mg/day versus external placebo controls at Week 78 in the whole study population and MCI-AD subpopulation.

Fig. 4 summarises ADAS-cog_11/13_ comparisons of HMTM 16 mg/day with external placebo arms trial controls overall including sensitivity analyses at 78 weeks in the whole population and in the MCI-AD subpopulation. Similar summary comparisons of whole brain volume loss (whole population and MCI-AD subpopulation) are shown in Supplementary Figure 7. As can be seen, MRI volume loss in placebo arms from the matched CPAD, unmatched CPAD (MAR and NMAR) and meta-analytic placebo populations are very similar. Comparisons with ADNI are not included because data were not available for 78 weeks, and the ADNI population represents natural history observational data rather than trial data. Overall, there is a high degree of concordance in the estimates of HMTM efficacy comparisons with treatment differences in the 5 – 6 ADAS-cog_11/13_ unit range and 6 – 9 cm^3^ in the range for WBV.

## 4 Discussion

For any trial with HMTM there is a trade-off between maintenance of blinding and tolerating the possibility of therapeutic activity in the control arm. In TRx-237–039, we elected to maintain blinding in compliance with strong regulatory recommendation by using the lowest dose of a compound able to mask urinary colouration, MTC 4 mg given twice weekly on a varying schedule. MTC is an oxidised form of methylthionine which can deliver HMT after redox conversion to the reduced form in the stomach. HMTM delivers HMT directly for absorption and distribution and is not subject to food interference [1]. Both MTC [6] and HMTM [7] have been shown to reverse scopolamine-induced cognitive deficits in a standard mouse model for symptomatic activity that does not have tau pathology. The mechanism for symptomatic activity of low dose MTC is therefore pharmacologically distinct from inhibition of tau aggregation. Clinical activity resulted from a combination of intrinsic symptomatic activity and atypical pharmacokinetics whereby plasma levels of HMT rose at 12 months to concentrations 50% above the lowest predicted human concentration at which symptomatic activity had been demonstrated in the scopolamine mouse model [6,7] The plasma levels in the MTC arm were too low to affect progression of neurodegeneration, grey matter atrophy and progression of tau pathology [8]. Although all of these biomarkers are known to be predictive of, and correlated with, clinical decline, there was a dissociation between the biological and clinical treatment effects of HMTM at 52 weeks. TRx-237–039 therefore demonstrates the infeasibility of conducting a standard “placebo-controlled” trial in which blinding is maintained without clinical activity in the control arm.

A complementary approach which offers a partial solution where a true placebo is not feasible without unblinding and resulting bias is to use an external control arm (ECA) consisting of true placebo subjects from external clinical trials who meet the same inclusion/exclusion criteria and are matched as closely as possible on important baseline characteristics. This can provide a trial emulation framework which permits a valid basis for evaluation of the clinical efficacy of the intervention which may be acceptable to regulators. In its recent Draft Guideline on the use of an ECA in circumstances where a conventional placebo-controlled comparison is infeasible, the MHRA states that if “data are sufficiently convincing then a positive decision can be reached even if alternative approaches may have ideally been preferred” [17]. According to the Draft Guideline, the ECA can use historical control data, may be identified after the clinical trial has finished and must be fully prespecified without risk of data selection being results driven. Caveats are similar to those stated by the FDA in its Guidance [18] which emphasises concerns about unmeasured confounding and data inconsistencies, and stresses the need for patient-level data, prespecification of inclusion criteria and statistical techniques that can take account of bias. According to the International Council for Harmonisation (ICH) guidelines [19] the disease should be well-documented with a highly predictable course and there should be a prior belief in the likely superiority of the intervention. We believe these conditions are met in the case of AD [20] and prior evidence of sustained cognitive benefit and significant treatment effects on underlying neurodegeneration in AD [8].

The only database available with granular subject-level data fit for this purpose is that provided by the Critical Path Institute (CPI). Of 9714 AD cases with placebo data in The Critical Path in AD (CPAD) database, 709 met the TRx-237–039 inclusion/exclusion criteria and had primary endpoint data to 78 weeks. Limitations of the CPAD database are that it does not provide data regarding intercurrent events or concurrent morbidities and the CPI does not permit disclosure of information which would permit identification of specific trials or geographies. Since the ECA data used required a positive amyloid β-PET scan as an inclusion criterion, it can be inferred from the meta-analyses we report that the relevant trials must have been conducted between 2015 – 2023, which overlaps the conduct of TRx-237–039 (2017 – 2023). Therefore, potentially confounding issues such as change in clinical diagnostic practice, trial assessment tools or the effect of COVID are unlikely to be important factors. We were able to ascertain from the CPI that in fact 96% of the matched CPAD data came from a single trial, minimising between-trial heterogeneity that could undermine generalisability.

We report the results of ECA comparisons using subject-level matched placebo data from the CPAD database. The selection of subjects from both sources was fully prespecified in protocol and SAP TRx-237–080. Although the outcomes of TRx-237–039 were known, selection of the cases used for the analysis from either data source could not be known a priori since this was predefined algorithmically prior to access to the CPAD database. This excludes any possibility of selection bias arising from knowledge of outcomes. The results of the strictly matched comparisons indicate clinical treatment effects on the order of 5 – 6 ADAS-cog_13_ units, 2 – 3 CDR-SB units and <20cm^3^ reduction in progression of brain volume loss over 78 – 104 weeks.

As these comparisons represent completers and therefore do not reflect ITT-based outcome estimates, we have undertaken extensive sensitivity analyses assuming that outcome data are missing not-at-random, and therefore potentially treatment-related. New propensity scores to permit matching that takes account of missing data were calculated using inverse probability weighting combined with covariate balancing (CBPS), Bayesian additive trees (BART) or logistic regression (LR). The CBPS approach provided the best matching of CPAD to HMTM data. Three different imputation approaches were calculated for each of the propensity score estimates. Of these we consider the most realistic to be the assumptions that participants retain treatment benefit up to the point of withdrawal and decline according to the CPAD trajectory thereafter. A conservative assumption is that there is no treatment benefit from baseline in any participant who withdrew after baseline. All estimated treatment effects were greater than 3 ADAS-cog_13_ units and greater than 4.9 cm^3^ for whole brain volume change. All were statistically significant at an alpha level of 0.0002 or less. The estimates in MCI with larger, at least 5.7 ADAS-cog_13_ units and 10 cm^3^ with p-values less than 0.004 and 0.0001 respectively. Therefore, the treatment effects were all statistically significant and clinically meaningful, even after taking account of increased uncertainty due to missing data.

The greatest potential vulnerability of comparisons using external control arms is the possibility that the differences can be explained by unmeasured confounders. This is circumvented in a standard clinical trial design by using random allocation of participants to active treatment or placebo. In the case of an ECA it is necessary to estimate the magnitude of potential confounding factors needed to explain away the measured differences. We show that it would be necessary to assume both that these presumed confounders are approximately 2 – 3 times more frequent in the HMTM arm than in the CPAD arm, and that they are independently associated with a two/three-fold increase in the risk of a worse outcome. Another approach suggested in the MHRA Draft Guideline (paragraph 49) is to replicate the results in two or more separate control arms. If the effect is explained by confounders, these would have to be simultaneously present in other controls as well, which is highly unlikely. For this purpose, we have used propensity score matching to compare with natural history ADNI data, and comparison with meta-analytic controls derived from placebo data from published clinical trials in comparable populations. Both of these provide confirmation of statistically significant treatment differences with respect to populations not receiving any form of HMT. The decline observed in the ADNI controls is smaller than in clinical trial populations and therefore the estimated treatment effects are numerically smaller. This is most likely explained by the ADNI database containing too few amyloid-PET positive participants and by exclusion of those with an MMSE score less than 20. By contrast, the comparisons with amyloid-PET positive participants, whether using CPAD data or meta-analytic controls, are comparable, despite the use of linear scaling of different versions of ADAS-cog to maximise number of trials compared and permitting analysis of the ADCS-ADL outcome. Several of the more recent studies did not rely on ADCS-ADL_23_ but on modified versions more suitable for the earlier stages of dementia due to AD.

The gold standard for assessment of risk-benefit for a new therapeutic intervention is an adequate and well-controlled trial which can support robust inferences by demonstrating statistically significant benefit whilst taking adequate account of missing data. This is usually a contemporaneous placebo-controlled trial in which participants are randomly allocated to the intervention or to an inert placebo. We demonstrated the efficacy of MTC 138 mg/day on clinical and neuroimaging endpoints in such a design using a true placebo [21]. Although information regarding urine colouration was withheld from efficacy raters, this approach was criticised by regulatory authorities because of the possibility of participant-bias in reporting of clinical outcomes on subjective scales. A further issue which emerged from the Phase 2 study was bias in retention of participants after 24-weeks [21].

The question at the heart of this report is whether an assessment of the benefit of an intervention based on external control comparisons can be valid. There are numerous possible criticisms of this approach. The most important is selection bias of participants included in the analysis. Another concern is the confounding effect of unknown factors that cannot be accounted for in the selection criteria for included participants. A further concern is that the populations compared are not cotemporaneous and are therefore subject to differences in methods of data collection, trial conduct, geography, trial sites, differences in application of clinical scales by assessors and differences in timing of trials. Another concern is methodological in that reasons for withdrawal are unknown in external data, precluding valid comparison of reasons for withdrawal. Further concerns can be raised specifically in connection with each of the comparisons presented. The selection of participants was conducted in the knowledge of the outcome of TRx-237–039 at 52-weeks, making the analysis post-hoc; it is worth emphasising however that the CPAD data was not accessed prior to finalising TRx-237–080 protocol and SAP. For the ADNI comparison, although fully specified prior to final database lock, the data available were observational and not obtained in trial-like conditions.

We have gone some way towards mitigating these concerns, and the consistency of results based on multiple different external comparison approaches is striking. The totality evidence already available presented in the accompanying paper [8] even without TRx-237–080 is statistically incompatible with the null hypothesis that hydromethylthionine 16 mg/day has no treatment effect. TRx-237–080 provides the closest true placebo data, where clinical decline over 78–104 weeks was 6–8 ADAS-cog_13_ units, 3–4 CDR-SB units, and 20–30 cm^3^ of whole brain volume loss. Meta-analyses of placebo arms showed similar decline: about 7 ADAS-cog_13_ units, 2–3 CDR-SB units, 7 ADCS-ADL_23_ units and roughly 20 cm³ of brain-volume loss. The differences in definitions of MCI used in TRx-237–039 and in the literature do not appear to be important. Despite wide variation in geography, study period, assessment methods, MRI protocols and concomitant AChEI/memantine use, these comparable trial populations show consistent rates of clinical decline and progression of whole brain volume loss. This is consistent with a meta-analytic study of placebo decline in 140 trials, which found a cognitive decline rate of 5 – 6 ADAS-cog_11_ units per annum in trials published before and after 2008 and between trials using an add-on design and those that did not use an add-on design after controlling for age and baseline cognitive function [20]. Clinical decline in patients treated with HMTM 16 mg/day was consistently, and highly statistically significantly, less than in all external population metrics, by 5–6 ADAS-cog_13_ units, 6 ADCS-ADL_23_ units and 2–3 CDR-SB units. Such differences are well above the minimum considered clinically meaningful in both MCI/early AD and mild/moderate AD [22], [23].

In summary, HMTM is a novel, orally administered, dual-action drug that targets both tau aggregation pathology and also has symptomatic activity. The conduct of a placebo-controlled trial design requires a trade-off between blinding and lack of confounding therapeutic activity. Comparisons with external placebo data using PSM offers the best available alternative which permit valid efficacy conclusions to be drawn if correctly performed and in accordance with regulatory guidelines. The consistency of benefit demonstrated on multiple clinical and biological endpoints makes the null hypothesis that HMTM lacks therapeutic efficacy implausible. The results we report show clinically meaningful differences of 5–6 ADAS-cog_13_ units and 2–3 CDR-SB units relative to placebo data from external trial populations. HMTM has the potential to provide an accessible oral treatment option with a benign safety profile which could be delivered with minimal patient/physician burden.

## Funding

The study was financed entirely by TauRx Therapeutics Ltd.

## Availability of data and materials

The datasets and analyses used during the current study are available from the corresponding author on reasonable request.

## Declaration of generative AI and AI-assisted technologies in the writing process

Generative AI and AI-assisted technologies were not used during the preparation of this manuscript.

## CRediT authorship contribution statement

**Bjoern O Schelter:** Writing – review & editing, Writing – original draft, Methodology, Conceptualization. **Helen Shiells:** Writing – review & editing, Writing – original draft, Methodology, Conceptualization. **Serena Lo:** Writing – review & editing, Writing – original draft, Methodology. **Nafeesa Nazlee:** Writing – review & editing, Writing – original draft. **Emily Evans:** Writing – review & editing, Writing – original draft, Methodology, Conceptualization. **Peter Bentham:** Writing – review & editing, Writing – original draft. **Serge Gauthier:** Writing – review & editing, Writing – original draft. **Henrik Zetterberg:** Writing – review & editing, Writing – original draft. **Gordon K Wilcock:** Writing – review & editing, Writing – original draft. **Lutz Froelich:** Writing – review & editing, Writing – original draft. **Alistair Burns:** Writing – review & editing, Writing – original draft. **Emer MacSweeney:** Writing – review & editing, Writing – original draft. **Clive Ballard:** Writing – review & editing, Writing – original draft. **Jin-Tai Yu:** Writing – review & editing, Writing – original draft. **Tay Siew Choon:** Writing – review & editing, Writing – original draft, Methodology. **Vahe Asvatourian:** Writing – review & editing, Methodology, Formal analysis, Conceptualization. **Natalia Muehlemann:** Writing – review & editing, Methodology, Formal analysis, Conceptualization. **Jan Priel:** Writing – review & editing, Methodology, Formal analysis, Conceptualization. **Karin Kook:** Writing – review & editing, Writing – original draft. **Tenecia Sullivan:** Writing – review & editing, Writing – original draft. **Diane Downie:** Writing – review & editing, Writing – original draft. **Sonya Miller:** Writing – review & editing, Writing – original draft. **Carol Pringle:** Writing – review & editing, Writing – original draft. **John M․D Storey:** Writing – review & editing, Writing – original draft. **Tom Baddeley:** Writing – review & editing, Writing – original draft. **Charles R Harrington:** Writing – review & editing, Writing – original draft. **Roger Staff:** Writing – review & editing, Writing – original draft, Methodology, Formal analysis. **Anca-Larisa Sandu:** Writing – review & editing, Methodology, Formal analysis. **Claire Hull:** Writing – review & editing, Writing – original draft. **Richard Stefanacci:** Writing – review & editing, Writing – original draft. **Claude M Wischik:** Writing – review & editing, Writing – original draft, Methodology, Conceptualization.

## Declaration of competing interest

The authors declare the following financial interests/personal relationships which may be considered as potential competing interests:

Bjoern Schelter reports a relationship with GT Diagnostics that includes: employment. Serge Gauthier reports a relationship with MBI-C that includes: equity or stocks. Serge Gauthier, Henrik Zetterberg reports a relationship with AbbVie Inc that includes: consulting or advisory. Serge Gauthier reports a relationship with ADvantage that includes: consulting or advisory. Serge Gauthier reports a relationship with AmyriAD that includes: consulting or advisory. Serge Gauthier, Lutz Froelich, Alistair Burns reports a relationship with Eisai Inc that includes: consulting or advisory. Serge Gauthier reports a relationship with ENIGMA that includes: consulting or advisory. Serge Gauthier, Lutz Froelich, Clive Ballard, Alistair Burns reports a relationship with Eli Lilly that includes: consulting or advisory. Serge Gauthier reports a relationship with Otsuka Pharmaceutical Co Ltd that includes: consulting or advisory. Serge Gauthier, Lutz Froelich, Clive Ballard, Henrik Zetterberg reports a relationship with Novo Nordisk Inc that includes: consulting or advisory. Lutz Froelich, Clive Ballard reports a relationship with Biogen Inc that includes:. Lutz Froelich reports a relationship with Hoffmann-LaRoche that includes: funding grants. Lutz Froelich reports a relationship with Hector II Foundation that includes: funding grants. Lutz Froelich reports a relationship with Dietmar Hopp Foundation that includes: funding grants. Lutz Froelich reports a relationship with BioVie that includes: consulting or advisory. Lutz Froelich reports a relationship with GE Healthcare that includes: consulting or advisory. Lutz Froelich reports a relationship with Grifols Inc that includes: consulting or advisory. Lutz Froelich reports a relationship with Janssen Pharmaceuticals Inc that includes: consulting or advisory. Lutz Froelich reports a relationship with Neurimmune AG that includes: consulting or advisory. Lutz Froelich reports a relationship with Noselab that includes: consulting or advisory. Lutz Froelich, Clive Ballard, Henrik Zetterberg reports a relationship with Roche that includes: consulting or advisory. Lutz Froelich reports a relationship with Schwabe that includes: consulting or advisory. Clive Ballard reports a relationship with Novartis that includes: funding grants. Clive Ballard reports a relationship with Johnson and Johnson that includes: consulting or advisory and funding grants. Clive Ballard reports a relationship with ReMynd that includes: funding grants. Clive Ballard reports a relationship with Acadia Pharmaceuticals Inc that includes: funding grants. Clive Ballard reports a relationship with Acadia Pharmaceuticals Inc that includes: consulting or advisory. Clive Ballard reports a relationship with AARP that includes: consulting or advisory. Clive Ballard reports a relationship with BMS Pharmaceutical Ltd that includes: consulting or advisory. Clive Ballard reports a relationship with Janssen Pharmaceuticals that includes: consulting or advisory. Clive Ballard reports a relationship with Orion Corp that includes: consulting or advisory. Clive Ballard reports a relationship with Exciva that includes: consulting or advisory. Clive Ballard reports a relationship with Sumitomo Pharma America Inc that includes: consulting or advisory. Clive Ballard reports a relationship with Suven Pharmaceuticals Limited that includes: consulting or advisory. Henrik Zetterberg reports a relationship with Swedish Research Council that includes: funding grants. Henrik Zetterberg reports a relationship with Horizon Europe that includes: funding grants. Henrik Zetterberg reports a relationship with Swedish State Support for Clinical Research that includes: funding grants. Henrik Zetterberg reports a relationship with Alzheimer’s Drug Discovery Foundation that includes: funding grants. Henrik Zetterberg reports a relationship with AD Strategic Fund that includes: funding grants. Henrik Zetterberg reports a relationship with Alzheimer’s Association that includes: funding grants. Henrik Zetterberg reports a relationship with Bluefield Project that includes: funding grants. Henrik Zetterberg reports a relationship with Cure Alzheimer’s Fund that includes: funding grants. Henrik Zetterberg reports a relationship with Olav Thon Foundation that includes: funding grants. Henrik Zetterberg reports a relationship with Erling Persson Family Foundation that includes: funding grants. Henrik Zetterberg reports a relationship with Stiftelsen för Gamla Tjänarinnor that includes: funding grants. Henrik Zetterberg reports a relationship with Hjärnfonden that includes: funding grants. Henrik Zetterberg reports a relationship with European Union Joint Programme that includes: funding grants. Henrik Zetterberg reports a relationship with National Institute for Health and Care Research University College London Hospitals Biomedical Research Centre that includes: funding grants. Henrik Zetterberg reports a relationship with UK Dementia Research Institute that includes: funding grants. Henrik Zetterberg reports a relationship with Acumen Pharmaceuticals Inc that includes: consulting or advisory. Henrik Zetterberg reports a relationship with Alector Inc that includes: consulting or advisory. Henrik Zetterberg reports a relationship with Alzinova AB that includes: consulting or advisory. Henrik Zetterberg reports a relationship with ALZpath Inc that includes: consulting or advisory. Henrik Zetterberg reports a relationship with Amylyx Pharmaceuticals Inc that includes: consulting or advisory. Henrik Zetterberg reports a relationship with Annexon Biosciences that includes: consulting or advisory. Henrik Zetterberg reports a relationship with Apellis Pharmaceuticals, Inc that includes: consulting or advisory. Henrik Zetterberg reports a relationship with Artery Therapeutics Inc that includes: consulting or advisory. Henrik Zetterberg reports a relationship with AZTherapies that includes: consulting or advisory. Henrik Zetterberg reports a relationship with Cognito Therapeutics Inc that includes: consulting or advisory. Henrik Zetterberg reports a relationship with CogRx that includes: consulting or advisory. Henrik Zetterberg reports a relationship with Denali Therapeutics Inc that includes: consulting or advisory. Henrik Zetterberg reports a relationship with LABCORP that includes: consulting or advisory. Henrik Zetterberg reports a relationship with Merry Life that includes: consulting or advisory. Henrik Zetterberg reports a relationship with NervGen Pharma Corp that includes: consulting or advisory. Henrik Zetterberg reports a relationship with Optoceutics that includes: consulting or advisory. Henrik Zetterberg reports a relationship with Passage Bio Inc that includes: consulting or advisory. Henrik Zetterberg reports a relationship with Pinteon Therapeutics Inc that includes: consulting or advisory. Henrik Zetterberg reports a relationship with Prothena that includes: consulting or advisory. Henrik Zetterberg reports a relationship with Quanterix Corp that includes: consulting or advisory. Henrik Zetterberg reports a relationship with Red Abbey Labs that includes: consulting or advisory. Henrik Zetterberg reports a relationship with reMYND that includes: consulting or advisory. Henrik Zetterberg reports a relationship with Samumed that includes: consulting or advisory. Henrik Zetterberg reports a relationship with Siemens Healthineers that includes: consulting or advisory. Henrik Zetterberg reports a relationship with Triplet Therapeutics Inc that includes: consulting or advisory. Henrik Zetterberg reports a relationship with Wave that includes: consulting or advisory. Henrik Zetterberg reports a relationship with Brain Biomarker Solutions that includes: equity or stocks. Claude M Wischik has patent issued to WisTa Laboratories Ltd. John M.D Storey, Charles R Harrington has patent issued to WisTa Laboratories Ltd. If there are other authors, they declare that they have no known competing financial interests or personal relationships that could have appeared to influence the work reported in this paper.

## Appendix Supplementary materials

Image, application 1

Image, application 2
