# Supplementary material for: Assessment of clinical and neuroimaging efficacy of treatment targeting tau pathology in mild cognitive impairment and mild to moderate Alzheimer’s disease with hydromethylthionine mesylate using external control data
Source: J Prev Alzheimers Dis. 2026 Apr 17;13(6):100560. doi: 10.1016/j.tjpad.2026.100560 (PMC13098421; doi:10.1016/j.tjpad.2026.100560)
Supplement: Supplementary file 1 [file mmc1.docx]

**Supplementary Appendix:**

TITLE: Assessment of clinical and neuroimaging efficacy of treatment targeting tau pathology in mild cognitive impairment and mild to moderate Alzheimer’s disease with hydromethylthionine mesylate using external control data

Authors: Bjoern O Schelter^1,2^, Helen Shiells^1^, Serena Lo^1,2^, Nafeesa Nazlee^1^, Emily Evans^1^, Peter Bentham^1,3^, Serge Gauthier^4^, Henrik Zetterberg^5,6,7,8,9,10^, Gordon K Wilcock^11^, Lutz Froelich^12^, Alistair Burns^13^, Emer MacSweeney^14^, Clive Ballard^15^, Jin-Tai Yu^16^, Tay Siew Choon^1^, Vahe Asvatourian^17^, Natalia Muehlemann^17^, Jan Priel^17^, Karin Kook^18^, Tenecia Sullivan^18^, Diane Downie^1^, Sonya Miller^1^, Carol Pringle^1^, John M.D Storey^1,19^, Tom Baddeley^1,19^, Charles R Harrington^1,20^, Roger Staff^21^, Anca-Larisa Sandu^22^, Claire Hull^1^, Richard Stefanacci^1,23^, Alzheimer's Disease Neuroimaging Initiative*** and Claude M Wischik^1,20^*

**Supplementary Methods**

**Statistical parametric mapping.** ADNI subjects to be compared with TRx-237-039 in this SPM analysis were selected via propensity score matching. A one-to-one nearest neighbour matching without replacement, with a caliper of 0.16 was adopted as a propensity score matching method. Both cohorts were matched using these covariates in the propensity score model: *APOε4* genotype, age, baseline MMSE, smoking history, and education. Additionally, only ADNI subjects that were not taking AChEIs and/or memantine were included in the matching.

T1 whole brain MRI images were processed according to a standard volumetric brain morphology pipeline using the Computational Anatomy Toolbox (CAT12) longitudinal integrated pipeline for voxel brain morphometry (VBM). The number of participants in each group who provided suitable images was 126 for HMTM 16 mg/day, 31 for 8 mg/day and 170 were available from ADNI. Data were compared using the longitudinal flexible factorial design. We constructed a design that had three groups (HMTM 16mg/day, 8mg/day and propensity-score matched ADNI) and two time points (Baseline and 52 weeks). Locations were assessed within the SPM interface where grey matter changes in subjects receiving HMTM (8- and 16-mg/day groups pooled) were smaller than reductions in the ADNI arm. We tested subjects with data at baseline as well as at 52 and 104 weeks for both the treatment and the ADNI groups.

**Meta-analysis study selection.** A systematic literature review (SLR) has been conducted and eligible publications identified from the SLR provided for analysis.

PubMed and the Cochrane Library were searched for relevant publications with search terms provided below. Records in clinicaltrials.gov were also hand-searched in case of omission. The inclusion criteria for the meta-analyses were as follows: 1) full-text publications written in English; 2) double-blind, placebo-controlled, randomized clinical trial; 3) patients with probable AD of mild, mild to moderate severity or MCI due to AD; 4) includes treatment duration for at least 52 weeks; and 5) a measure for ADAS-cog, ADL or brain atrophy (whole brain). Studies were excluded for the following reasons: 1) not randomized controlled trials, such as case reports, reviews, or meta-analysis; 2) publications without sufficient information or unable to extract valid outcome data.

The following publication information and patient characteristics were extracted from all studies included: author's name, publication year, sample size, baseline MMSE and diagnostic criteria for AD (amyloid positivity). The following outcome data were extracted where available: change from baseline in whole brain volume, ADAS-cog_11_, ADAS-cog_13_, and Alzheimer’s Disease Cooperative Study – Activities of Daily Living (23-item) (ADCS-ADL_23_).

**Statistical analysis**

Data from the HMTM 16 mg/day and MTC 8 mg/week arms of TRx-237-039 (E-MITT population) were compared against a meta-analysis-pooled placebo data from published studies, in terms of change from baseline of ADAS-cog_11_, ADAS-cog_13_, ADCS-ADL_23_ and whole brain volume at 6 months, 12 months and 18 months. Comparisons were performed using a two-sided independent samples t-test with a significance level of 5%.

Inverse Propensity Score Weighting

To adjust for baseline differences in measured confounders between the treated trial cohort and the external control arm, we used inverse propensity score weighting (IPW) targeting the average treatment effect (ATE) [1, 2, 3]. Let $A_{i}$ denote the treatment indicator and $X_{i}$ a vector of baseline covariates of patient $i$. The propensity score (PS) of patient $i$ is $\pi\left( X_{i} \right)=\Pr\left( A_{i}=1 \mid X_{i} \right)$. Weights targeting ATE based on the PS are defined as,

$$w_{i}=\left\{ \begin{matrix} 1/\pi(X_{i}), & A_{i}=1 \\ 1/\{1-\pi(X_{i})\}, & A_{i}=0 \end{matrix} \right.$$

Stabilized weights are defined as:

$$w_{i}=\left\{ \begin{matrix} \Pr(A=1)/\pi(X_{i}), & A_{i}=1 \\ \Pr(A=0)/\{1-\pi(X_{i})\}, & A_{i}=0 \end{matrix} \right.$$

where $Pr(A=1)$ and $Pr(A=0)$ are the marginal probabilities of treatment and control in the overall sample. Stabilization is used to reduce variability and increase the effective sample size [4].

1. Austin, P.C., An Introduction to Propensity Score Methods for Reducing the Effects of Confounding in Observational Studies. Multivariate Behav Res, 2011. 46(3): p. 399-424.
2. Robins JM, Hernán MA, Brumback B. Marginal structural models and causal inference in epidemiology. Epidemiology. 2000;11(5):550-560. doi:10.1097/00001648-200009000-00011. PMID:10955408.
3. Hernán MA, Robins JM. Causal Inference: What If. Boca Raton, FL: Chapman & Hall/CRC; 2020. (Online; continuously updated.) https://miguelhernan.org/whatifbook
4. Austin PC, Stuart EA. Moving towards best practice when using inverse probability of treatment weighting (IPTW) using the propensity score to estimate causal treatment effects in observational studies. Stat Med. 2015 Dec 10;34(28):3661-79. doi: 10.1002/sim.6607. Epub 2015 Aug 3. PMID: 26238958; PMCID: PMC4626409.

**E-values**

E-values were computed for a difference of means and for its 95% confidence interval limit closest to the null with the R package Evalue [7]. For continuous outcomes, the package uses the effect-size conversions [5, 6] to convert the mean difference between the exposed versus unexposed cohorts to the odds ratio that would arise from dichotomizing the continuous outcome.

1. Chinn, S (2000). A simple method for converting an odds ratio to effect size for use in meta-analysis. Statistics in Medicine, 19(22), 3127-3131.
2. VanderWeele, TJ (2017). On a square-root transformation of the odds ratio for a common outcome. Epidemiology, 28(6), e58.
3. Mathur MB, Ding P, Riddell CA, VanderWeele TJ. Web Site and R Package for Computing E-values. Epidemiology. 2018 Sep;29(5):e45-e47. doi: 10.1097/EDE.0000000000000864. PMID: 29912013; PMCID: PMC6066405.

**Search strategy for PubMed and Cochrane database**

| Database | Search strategy |
| --- | --- |
| PubMed | ((((((((((((((((((Alzheimer Diseases)) OR (Alzheimers Diseases)) OR (Alzheimer Disease)) OR (Alzheimer's Disease)) OR (mild dementia)) OR (Alzheimer Diseases)) OR (Dementia Alzheimer Type)) OR (Alzheimer Type Dementia)) OR (Alzheimer Dementia)) OR (Mild Cognitive Impairment)) OR (mild cognitive impairment)) OR (mild Alzheimer's Disease)) OR (Alzheimer Disease)) OR (Alzheimer Dementias)) OR (Dementia Alzheimer)) OR (Early Alzheimer's Disease)) OR (MCI)) AND ((((((clinical trial) OR (randomized controlled trial)) OR (randomised controlled trial)) OR (RCT)) OR (double-blind)) OR (double blind)) AND (((placebo controlled) OR (placebo)) OR (placebo-controlled )) AND (((((((((((((((((ADAS-Cog) OR (ADAS-cog11)) OR (ADAS-cog12)) OR (ADAS-cog14)) OR (ADAS-cog13)) OR (Alzheimer's Disease Assessment Scale)) OR (ADAS)) OR (ADCS-ADL)) OR (ADCS-MCI-ADL)) OR (Whole brain volume)) OR (Daily Living Scale)) OR (WBV)) OR (Activities of Daily Living Scale)) OR (MRI)) OR (volumetric MRI)) OR (magnetic resonance imaging)) OR (ADL)) OR (Alzheimer's Disease Cooperative Study-Activities of Daily Living Scale)) |
| Cochrane Library | Search term: ‘Alzheimer’s Disease’ or ‘MCI’ or ‘ADAS’  Filter applied: ‘clinical trials’ |

**Studies included in Meta-analysis**

| **Citation**  **(Sponsor)** | **Study Design (Region)**  **Acronym** | **Population (as Described in Publication)** | **Endpoints Rated** | **Meta-analysis** | |  |
| --- | --- | --- | --- | --- | --- | --- |
|  |  |  |  | **Cytel** | **TauRx** | **TauRx**  **Anti-amyloid** |
| Aisen et al., 2003  (NIA) [1] | 1-year, placebo-controlled study (vs rofecoxib or naproxen) (US) | Mild-Mod AD (MMSE 13-26) | ADAS-cog_11_  ADCS-ADL | -- | × | -- |
| Aisen et al., 2008  (NIA) [2] | 18-month, placebo-controlled study (vs high-dose vitamin B (US) | Mild-Mod AD (MMSE 14-26) | ADAS-cog_11_  ADCS-ADL | -- | × | -- |
| Budd Haeberlein et al., 2022 (Biogen, Inc) [3] | 18-month, placebo-controlled study  (vs aducanumab) (Multinational)  ENGAGE, EMERGE | MCI-AD  Mild AD  (MMSE 24-30,  CDR 0.5) | ADAS-cog_13_,  ADCS-ADL-MCI  WBV | × | × | -- |
| Burns et al., 1999  (Eisai Inc) [4] | 30-week, placebo-controlled study (vs donepezil) | Mild-severe AD  (MMSE 10–26) | ADAS-cog | -- | × | -- |
| Burstein et al., 2014  (Pfizer, Inc., NIA) [5] | 18-month, placebo-controlled study (vs TTP488) (US) | Mild-Mod AD  (MMSE 14-26) | ADAS-cog_11_,  ADCS-ADL | -- | × | -- |
| Coric et al., 2015  (Bristol-Myers Squibb) [6] | 104-week, placebo-controlled study (vs avagacestat) (Multinational) | MCI-AD  (MMSE 24-30,  CDR 0.5) | WBV | × | × | -- |
| De Jong et al., 2008  (Academic) [7] | 1-year, placebo-controlled study (vs indomethacin) (Netherlands) | Moderate AD  (MMSE 10-26) | ADAS-cog_11_ | -- | × | -- |
| Doody et al., 2008 [8] | 26-week, placebo-controlled study (vs Dimebon) | Mild-Mod AD  (MMSE 10-24) | ADAS-cog,  ADCS-ADL | -- | × | -- |
| Doody et al., 2013  (Eli Lilly and Company) [9] | 76-week, placebo-controlled study (vs semagacestat); stopped early due to safety (Multinational) | Mild-Mod AD (MMSE 16-26) | ADCS-ADL | -- | × | -- |
| Doody et al., 2014  (Eli Lilly and Company, ACDS) [10] | 18-month, placebo-controlled study (vs solanezumab) (US)  EXPEDITION1 | Mild-Mod AD (MMSE 14-26) | ADAS-cog_11_  ADCS-ADL  WBV | -- | × | -- |
|  | EXPEDITION2 |  | ADAS-cog_14_  ADCS-ADL  WBV | -- | × | -- |
| Egan et al., 2018  (Merck) [11] | 78-week, placebo-controlled study (vs verubecestat) (Multinational) | Mild-Mod AD  (MMSE 15-26) | ADAS-cog_11_,  ADCS-ADL | -- | × | -- |
| Egan et al., 2019  (Merck) [12] | 104-week, placebo-controlled study (vs verubecestat); closed for futility  (Multinational) | Prodromal AD  (RBANS ≤85) | ADCS-ADL-MCI | -- | × | -- |
| Feldman et al., 2010  (Pfizer, Inc.) [13] | 72-week, placebo-controlled study (vs atorvastatin) (Multinational)  LEADEe | Mild-Mod AD (MMSE 13-25) | ADAS-cog_11_ | -- | × | -- |
| Gold et al., 2010  (GlaxoSmithKline) [14] | 24-week, placebo-controlled study (vs Rosiglitazone) | Mild-Mod AD (MMSE 10-23) | ADAS-cog | -- | × | -- |
| Green et al., 2009  (Myriad Pharmaceuticals) [15] | 18-month, placebo-controlled study (vs Tarenflurbil) (US) | Mild AD  (MMSE 20-26) | ADCS-ADL | -- | × | -- |
| Harrington et al., 2011  (Glaxosmith Kline) [16] | 48-week, placebo-controlled study (vs rosiglitazone) (multinational) | Mild-Mod AD  (MMSE 10-26) | ADAS-cog_11_ | -- | × | -- |
|  | 48-week, placebo-controlled study (vs rosiglitazone) (multinational) | Mild-Mod AD  (MMSE 10-26) | ADAS-cog_11_ | -- | × | -- |
| Hoing et al., 2018  (Eli Lilly) [17] | 76-week, placebo-controlled study (vs Solanezumab) (Multinational) | Mild AD  (MMSE 20-26) | ADCS-iADL  WBV | -- | × | -- |
| Imbimbo et al., 1998  (Pharmacia & Upjohn) [18] | 25-week, placebo-controlled study (vs Eptastigmine) | Mild-Mod AD  (MMSE 10-26) | ADAS-cog,  ADCS-ADL | -- | × | -- |
| Karaman et al., 2005  (Academic) [19] | 12-month, placebo-controlled study (vs rivastigmine) (Turkey) | Mild-Mod AD  (MMSE 14-30) | ADCS-ADL | -- | × | -- |
| Kehoe et al., 2021  (Academic) [20] | 12-months, placebo-controlled study (vs losartan) (UK) | Mild-Mod AD  (MMSE 15-28) | WBV | -- | × | -- |
| Knapp et al., 1994 [21] | 30-week, placebo-controlled study (vs Tacrine) | Mild-Mod AD  (MMSE 10-26) | ADAS-cog | -- | × | -- |
| Lawlor et al., 2018  (Academic) [22] | 78-week, placebo-controlled study (vs nilvadipine) (Europe) | Mild-Mod AD (MMSE 12-26) | ADAS-cog_12_ | -- | × | -- |
| Mintun et al., 2021  (Eli Lilly and Company)  [23] | 72-week, placebo-controlled study (vs donanemab) (Canada, US)  (TRAILBLAZER-ALZ) | MCI-AD – Mild AD  (MMSE 20-28) | ADAS-cog_13_,  ACDS-iADL  WBV | × | × | × |
| Moreno Moreno et al., 2003 (Italfarmaco SpA) [24] | 6-month, placebo-controlled study (vs Choline Alfoscerate) | Mild-Mod AD  (MMSE 12-26) | ADAS-cog | -- | × | -- |
| Nave et al., 2017  (F. Hoffmann-La Roche Ltd) [25] | 52-week, placebo-controlled study (vs Sembragiline) (Multinational) | Moderate AD  (MMSE 13-20) | ADCS-ADL | -- | × | -- |
| Novak et al., 2020 (Janssen Research & Development, LLC) [26] | 12-months, placebo-controlled study (vs atabecestat) | MCI-AD–Mild AD  (CDR global≤0.5) | WBV | -- | × | -- |
| Ostrowitzski et al., 2022  (AC Immune SA, Genentech, Hoffmann-La Roche) [27] | 105-week, placebo-controlled study (vs crenezumab) (Multinational)  CREAD | Prodromal AD or mild AD  (MMSE ≥22) | ADCS-ADL | -- | × | -- |
|  | 105-week, placebo-controlled study (vs crenezumab) (Multinational)  CREAD2 | Prodromal AD or mild AD  (MMSE ≥22) | ADCS-ADL | -- | × | -- |
| Panisset et al., 2002  (EBEWE Arzneimittel GmbH) [28] | 6-month, placebo-controlled study (vs Cerebrolysin) | Mild-Mod AD  (MMSE 10-26) | ADAS-cog | -- | × | -- |
| Peskind et al., 2006  (Forest Research Institute) [29] | 24-week, placebo-controlled study (vs memantine) | Mild-Mod AD  (MMSE 10-22) | ADCS-ADL | -- | × | -- |
| Raskind et al., 2000  (Janssen Research Foundation) [30] | 6-month, placebo-controlled study (vs Galantamine) | Mild-Mod AD  (MMSE 11-24) | ADAS-cog | -- | × | -- |
| Relkin et al., 2017  (Baxalta & NIA) [31] | 18-month, placebo-controlled study (vs immunoglobulin) (US & Canada) | Mild-Mod AD  (MMSE 16-26) | ADAS-cog_11_  ADCS-ADL | -- | × | -- |
| Rockwood et al., 1997  (Merck & Co., Inc) [32] | 6-month, placebo-controlled study (vs Linopirdine) | Mild-Mod AD  (MMSE 10-23) | ADAS-cog,  ADCS-ADL | -- | × | -- |
| Rogers et al., 1998  (Eisai Inc. and Pfizer Inc) [33] | 24-week, placebo-controlled study (vs Donepezil) | Mild-Mod AD  (MMSE 10-26) | ADAS-cog | -- | × | -- |
| Salloway et al., 2014  (Genentech, Inc.) [34] | 78-week, placebo-controlled study, one in APOE carriers and one in noncarriers (vs bapineuzumab)  (Multinational) | Mild-Mod AD  (MMSE 16-26) | ADAS-cog_11_  WBV | -- | × | -- |
| Salloway et al., 2018  Janssen Alzheimer Immunotherapy R&D and Pfizer Inc. [35] | 73-week, placebo-controlled study (vs crenezumab) (France, Spain, US)  BLAZE | Mild-Mod AD  (MMSE 18-26, CDR-sb ≥0.5) | ADAS-cog_12_ | -- | × | -- |
|  | 73-week, placebo-controlled study (vs crenezumab) (France, Spain, US)  BLAZE | Mild-Mod AD  (MMSE 18-26, CDR-sb ≥0.5) | ADAS-cog_12_ | -- | × | -- |
| Sano et al., 2011  (ADCS) [36] | 18-month, placebo-controlled study (vs simvastatin) (US) | Mild-Mod AD  (MMSE 12-26) | ADAS-cog_11_  ADCS-ADL | -- | × | -- |
| Schneider et al., 2005  (Willmar Schwabe GmbH & Co. KG) [37] | 26-week, placebo-controlled study (vs Ginkgo biloba) | Mild-Mod AD  (MMSE 10–24) | ADAS-cog | -- | × | -- |
| Shulman et al., 2023  (Biogen, Inc.) [38] | 78-week, placebo-controlled study (vs gosuranemab) (Multinational)  TANGO | MCI-AD – Mild AD  (MMSE 22-30, CDR 0.5 or 1) | ADAS-cog_13_  ADCS-ADL  WBV | -- | × | -- |
| Sims et al., 2023  (Eli Lilly and Company) [39] | 76-week, placebo-controlled study (vs donanemab) (Multinational)  TRAILBLAZER-ALZ 2 | MCI-AD – Mild AD  (MMSE 20-28) | ADAS-cog_13_  ACDS-iADL  WBV | × | × | × |
| Soininen et al., 2007  (Pfizer Inc) [40] | 52-week, placebo-controlled study (vs Celecoxib) (Multinational) | Mild-Mod AD (MMSE 12-26) | ADAS-cog_11_ | -- | × | -- |
| Sparks et al., 2005  (Pfizer Inc) [41] | 12-months, placebo-controlled study (vs atorvastatin) (US) | Mild-Mod AD  (MMSE 12-28) | ADAS-cog_11_ | -- | × | -- |
| Sperling et al., 2021  (Janssen Research & Development, LLC) [42] | 24-month, placebo-controlled study (vs Atabecestat) | Preclinical AD  (CDR global=0) | WBV | -- | × | -- |
| Sur et al., 2020  (Merck Sharp & Dohme Corp) [43] | 78-week, placebo-controlled study (vs verubecestat) (Multinational) | Mild-Mod AD  (MMSE 15-26) | WBV | -- | × | -- |
| Swanson et al., 2021  (Eisai Inc) [44] | 18-month, placebo-controlled, response-adaptive Bayesian design (vs lecanemab) (Multinational) | MCI-AD, Mild AD  (MMSE ≥22) | ADAS-cog_14_  WBV | -- | × | × |
| Tariot et al., 2000  (Janssen Research Foundation) [45] | 5-month placebo-controlled study (vs Galantamine) | Mild-Mod AD  (MMSE 10-22) | ADAS-cog,  ADCS-ADL | -- | × | -- |
| Teng et al., 2022  (Genentech, Inc) [46] | 73-week, placebo-controlled study (vs semorinemab) (Multinational) | Prodromal AD or mild AD  (MMSE 20-30) | ADCS-ADL | -- | × | -- |
| Thal et al., 1996  (Sigma tau pharmaceuticals Inc) [47] | 12-month, placebo-controlled study (vs resveratrol) (US) | Mild-Mod AD  (MMSE 14-26) | ADAS-cog_11_  ADCS-ADL | -- | × | -- |
| Thal et al.,1999  (Forest Laboratories) [48] | 24-week, placebo-controlled study (vs Physostigmine) | Mild-Mod AD  (MMSE 12-24) | ADAS-cog | -- | × | -- |
| Thal et al., 2003  (NIA & Takeda America, Inc) [49] | 1-year, placebo-controlled study (vs idebenone) (US) | Mild-Mod AD  (MMSE 12-25) | ADAS-cog_11_  ADCS-ADL | -- | × | -- |
| Thal et al., 2000  (Academic) [50] | 6-month, placebo-controlled study (vs Lu25-109) (US) | Mild-Mod AD  (MMSE 10-26) | ADAS-cog_11_  ADCS-ADL | -- | × | -- |
| Thal et al., 2000  (Sigma-Tau Pharmaceuticals, Inc) [51] | 12-month, placebo-controlled study (vs Acetyl-L-Carnitine 3) | Mild-Mod AD  (MMSE 12-26) | ADAS-cog,  ADCS-ADL | -- | × | -- |
| Turner et al., 2015  (Academic) [52] | 52-week, placebo-controlled study (vs resveratrol) (US) | Mild-Mod AD  (MMSE 14-26) | WBV | -- | × | -- |
| Van Dyck et al., 2016  (Pfizer Inc.) [53] | 24-month, placebo-controlled study (vs ACC-001) | Prodromal AD or MCI-AD  (MMSE ≥25) | ADAS-cog  WBV | -- | × | -- |
| Van Dyck et al., 2022  (Eisai & Biogen, Inc) [54] | 18-month, placebo-controlled study (vs lecanemab) (Multinational) | MCI-AD – Mild AD  (MMSE ≥22) | ADCS-ADL-MCI  WBV | -- | × | × |
| vTv therapeutics [55] | 18-month, placebo-controlled study (vs azeliragon) (Multinational) | Mild AD  (MMSE 21-26) | ADCS-ADL | -- | × | -- |
| Vandenberghe et al., 2016  (Pfizer Inc. and Janssen Alzheimer Immunotherapy R&D) [56] | 18-month, placebo-controlled study, one in APOE e4 carriers and one in noncarriers (vs bapineuzumab)  (Multinational) | Mild-Mod AD  (MMSE 16-26) | ADAS-cog_11_  WBV | -- | × | -- |
| Wessels et al., 2020  (Eli Lilly and Company and AstraZenica) [57] | 104-week, placebo-controlled study (vs lanabecestat) (Multinational)  (AMARANTH) | MCI-AD, Mild AD  (MMSE 20-30) | ADAS-cog_13_  ACDS-iADL | × | × | -- |
|  | 78-week, placebo-controlled study (vs lanabecestat) (Multinational)  (DAYBREAK-ALZ) | Mild AD  (MMSE 20-26) | ADAS-cog_13_  ACDS-iADL | × | × | -- |
| Wilcock et al., 2008  (Myriad Pharmaceuticals) [58] | 12-month, placebo-controlled study (vs tarenﬂurbil) (Multinational) | Mild-Mod AD  (MMSE 15-26) | ADAS-cog_11_ | -- | × | -- |
| Wilcock et al., 2000  (Janssen Research Foundation) [59] | 6-month, placebo-controlled study (vs galantamine) | Mild-Mod AD  (MMSE 11-24) | ADAS-cog | -- | × | -- |

**Meta-analysis Reference list**

[1] Aisen PS, Schafer KA, Grundman M, Pfeiffer E, Sano M, Davis KL, et al. Effects of rofecoxib or naproxen vs placebo on Alzheimer disease progression: A randomized controlled trial. JAMA 2003;289:2819–26. https://doi.org/10.1001/jama.289.21.2819.

[2] Aisen PS, Schneider LS, Sano M, Diaz-Arrastia R, van Dyck CH, Weiner MF, et al. High-dose B vitamin supplementation and cognitive decline in Alzheimer disease: a  randomized controlled trial. JAMA 2008;300:1774–83. https://doi.org/10.1001/jama.300.15.1774.

[3] Budd Haeberlein S, Aisen PS, Barkhof F, Chalkias S, Chen T, Cohen S, et al. Two randomized phase 3 studies of aducanumab in early Alzheimer’s disease. J Prev Alzheimers Dis 2022;9:197–210. https://doi.org/10.14283/jpad.2022.30.

[4] Burstein AH, Grimes I, Galasko DR, Aisen PS, Sabbagh M, Mjalli AM. Effect of TTP488 in patients with mild to moderate Alzheimer’s disease. BMC Neurol 2014;14:12. https://doi.org/10.1186/1471-2377-14-12.

[5] Coric V, Salloway S, Van Dyck CH, Dubois B, Andreasen N, Brody M, et al. Targeting prodromal Alzheimer disease with avagacestat: A randomized clinical trial. JAMA Neurol 2015;72:1324–33. https://doi.org/10.1001/jamaneurol.2015.0607.

[6] de Jong D, Jansen R, Hoefnagels W, Jellesma-Eggenkamp M, Verbeek M, Borm G, et al. No effect of one-year treatment with indomethacin on Alzheimer’s disease progression: A randomized controlled trial. PLoS One 2008;3:e1475–e1475. https://doi.org/10.1371/journal.pone.0001475.

[7] Doody RS, Gavrilova SI, Sano M, Thomas RG, Aisen PS, Bachurin SO, et al. Effect of dimebon on cognition, activities of daily living, behaviour, and global function in patients with mild-to-moderate Alzheimer’s disease: a randomised, double-blind, placebo-controlled study. The Lancet 2008;372:207–15. https://doi.org/10.1016/S0140-6736(08)61074-0.

[8] Doody RS, Raman R, Farlow M, Iwatsubo T, Vellas B, Joffe S, et al. A Phase 3 Trial of Semagacestat for Treatment of Alzheimer’s Disease. New England Journal of Medicine 2013;369:341–50. https://doi.org/10.1056/nejmoa1210951.

[9] Egan MF, Kost J, Tariot PN, Aisen PS, Cummings JL, Vellas B, et al. Randomized Trial of Verubecestat for Mild-to-Moderate Alzheimer’s Disease. New England Journal of Medicine 2018;378:1691–703. https://doi.org/10.1056/nejmoa1706441.

[10] Egan MF, Kost J, Voss T, Mukai Y, Aisen PS, Cummings JL, et al. Randomized Trial of Verubecestat for Prodromal Alzheimer’s Disease. New England Journal of Medicine 2019;380:1408–20. https://doi.org/10.1056/nejmoa1812840.

[11] Feldman HH, Doody RS, Kivipelto M, Sparks DL, Waters DD, Jones RW, et al. Randomized controlled trial of atorvastatin in mild to moderate Alzheimer disease: LEADe. Neurology 2010;74:956–64. https://doi.org/10.1212/WNL.0b013e3181d6476a.

[12] Gold M, Alderton C, Zvartau-Hind M, Egginton S, Saunders AM, Irizarry M, et al. Rosiglitazone monotherapy in mild-to-moderate alzheimer’s disease: Results from a randomized, double-blind, placebo-controlled phase III study. Dement Geriatr Cogn Disord 2010;30:131–46. https://doi.org/10.1159/000318845.

[13] Green RC, Schneider LS, Amato DA, Beelen AP, Wilcock G, Swabb EA, et al. Effect of tarenflurbil on cognitive decline and activities of daily living in patients with mild Alzheimer disease: A randomized controlled trial. JAMA 2009;302:2557–64. https://doi.org/10.1001/jama.2009.1866.

[14] Harrington C, Sawchak S, Chiang C, Davies J, Donovan C, M. Saunders A, et al. Rosiglitazone Does Not Improve Cognition or Global Function when Used as Adjunctive Therapy to AChE Inhibitors in Mild-to-Moderate Alzheimers Disease: Two Phase 3 Studies. Curr Alzheimer Res 2011;8:592–606. https://doi.org/10.2174/156720511796391935.

[15] Honig LS, Vellas B, Woodward M, Boada M, Bullock R, Borrie M, et al. Trial of Solanezumab for Mild Dementia Due to Alzheimer’s Disease. New England Journal of Medicine 2018;378:321–30. https://doi.org/10.1056/nejmoa1705971.

[16] Imbimbo BP, Lucca U, Lucchelli F, Alberoni M, Thal LJ. A 25-week placebo-controlled study of eptastigmine in patients with Alzheimer disease. Alzheimer Dis Assoc Disord 1998;12:313–22. https://doi.org/10.1097/00002093-199812000-00011.

[17] Karaman Y, Erdoǧan F, Köseoǧlu E, Turan T, Ersoy AÖ. A 12-month study of the efficacy of rivastigmine in patients with advanced moderate alzheimer’s disease. Dement Geriatr Cogn Disord 2005;19:51–6. https://doi.org/10.1159/000080972.

[18] Kehoe PG, Turner N, Howden B, Jarutyte L, Clegg SL, Malone IB, et al. Safety and efficacy of losartan for the reduction of brain atrophy in clinically diagnosed Alzheimer’s disease (the RADAR trial): a double-blind, randomised, placebo-controlled, phase 2 trial. Lancet Neurol 2021;20:895–906. https://doi.org/10.1016/S1474-4422(21)00263-5.

[19] Knapp MJ, Knopman DS, Solomon PR, Pendlebury WW, Davis CS, Gracon SI. A 30-Week Randomized Controlled Trial of High-Dose Tacrine in Patients With Alzheimer’s Disease. JAMA: The Journal of the American Medical Association 1994;271:985–91. https://doi.org/10.1001/jama.1994.03510370037029.

[20] Lawlor B, Segurado R, Kennelly S, Olde Rikkert MGM, Howard R, Pasquier F, et al. Nilvadipine in mild to moderate Alzheimer disease: A randomised controlled trial. PLoS Med 2018;15:1–20. https://doi.org/10.1371/journal.pmed.1002660.

[21] Mintun MA, Lo AC, Duggan Evans C, Wessels AM, Ardayfio PA, Andersen SW, et al. Donanemab in early Alzheimer’s disease. New England Journal of Medicine 2021;384:1691–704. https://doi.org/10.1056/NEJMoa2100708.

[22] Moreno Moreno MDJ. Cognitive improvement in mild to moderate Alzheimer’s dementia after treatment with the acetylcholine precursor choline alfoscerate: A multicenter, double-blind, randomized, placebo-controlled trial. Clin Ther 2003;25:178–93. https://doi.org/10.1016/S0149-2918(03)90023-3.

[23] Nave S, Doody RS, Boada M, Grimmer T, Savola JM, Delmar P, et al. Sembragiline in Moderate Alzheimer’s Disease: Results of a Randomized, Double-Blind, Placebo-Controlled Phase II Trial (MAyflOwer RoAD). Journal of Alzheimer’s Disease 2017;58:1217–28. https://doi.org/10.3233/JAD-161309.

[24] Novak G, Streffer JR, Timmers M, Henley D, Brashear HR, Bogert J, et al. Long-term safety and tolerability of atabecestat (JNJ-54861911), an oral BACE1 inhibitor, in early Alzheimer’s disease spectrum patients: A randomized, double-blind, placebo-controlled study and a two-period extension study. Alzheimers Res Ther 2020;12:58. https://doi.org/10.1186/s13195-020-00614-5.

[25] Ostrowitzki S, Bittner T, Sink KM, Mackey H, Rabe C, Honig LS, et al. Evaluating the Safety and Efficacy of Crenezumab vs Placebo in Adults With Early Alzheimer Disease. JAMA Neurol 2022;79:1113. https://doi.org/10.1001/jamaneurol.2022.2909.

[26] Panisset M, Gauthier S, Moessler H, Windisch M. Cerebrolysin in Alzheimer’s disease: A randomized, double-blind, placebo-controlled trial with a neurotrophic agent. J Neural Transm 2002;109:1089–104. https://doi.org/10.1007/s007020200092.

[27] Peskind ER, Potkin SG, Pomara N, Ott BR, Graham SM, Olin JT, et al. Memantine treatment in mild to moderate Alzheimer disease: A 24-week randomized, controlled trial. American Journal of Geriatric Psychiatry 2006;14:704–15. https://doi.org/10.1097/01.JGP.0000224350.82719.83.

[28] Raskind MA, Peskind ER, Wessel T, Yuan W. Galantamine in AD: A 6-month randomized, placebo-controlled trial with a 6-month extension. Neurology 2000;54:2261–8. https://doi.org/10.1212/WNL.54.12.2261.

[29] Relkin NR, Thomas RG, Rissman RA, Brewer JB, Rafii MS, Van Dyck CH, et al. A phase 3 trial of IV immunoglobulin for Alzheimer disease. Neurology 2017;88:1768–75. https://doi.org/10.1212/WNL.0000000000003904.

[30] Rockwood K, Beattie BL, Eastwood MR, Feldman H, Mohr E, Pryse-Phillips W, et al. A randomized, controlled trial of linopirdine in the treatment of Alzheimer’s disease. Canadian Journal of Neurological Sciences 1997;24:140–5. https://doi.org/10.1017/S031716710002148X.

[31] Rogers SL, Farlow MR, Doody RS, Mohs R, Friedhoff LT. A 24-week, double-blind, placebo-controlled trial of donepezil in patients with Alzheimer’s disease. Neurology 1998;50:136–45. https://doi.org/10.1212/WNL.50.1.136.

[32] Salloway S, Sperling R, Fox NC, Blennow K, Klunk W, Raskind M, et al. Two phase 3 trials of bapineuzumab in mild-to-moderate Alzheimer’s disease. New England Journal of Medicine 2014;370:322–33. https://doi.org/10.1056/nejmoa1304839.

[33] Salloway S, Honigberg LA, Cho W, Ward M, Friesenhahn M, Brunstein F, et al. Amyloid positron emission tomography and cerebrospinal fluid results from a crenezumab anti-amyloid-beta antibody double-blind, placebo-controlled, randomized phase II study in mild-to-moderate Alzheimer’s disease (BLAZE). Alzheimers Res Ther 2018;10:96. https://doi.org/10.1186/s13195-018-0424-5.

[34] Sano M, Bell KL, Galasko D, Galvin JE, Thomas RG, Van Dyck CH, et al. A randomized, double-blind, placebo-controlled trial of simvastatin to treat Alzheimer disease. Neurology 2011;77:556–63. https://doi.org/10.1212/WNL.0b013e318228bf11.

[35] Schneider L, DeKosky S, Farlow M, Tariot P, Hoerr R, Kieser M. A Randomized, Double-Blind, Placebo-Controlled Trial of Two Doses of Ginkgo Biloba Extract in Dementia of the Alzheimers Type. Curr Alzheimer Res 2005;2:541–51. https://doi.org/10.2174/156720505774932287.

[36] Shulman M, Kong J, O’Gorman J, Ratti E, Rajagovindan R, Viollet L, et al. TANGO: a placebo-controlled randomized phase 2 study of efficacy and safety of the anti-tau monoclonal antibody gosuranemab in early Alzheimer’s disease. Nat Aging 2023;3:1591–601. https://doi.org/10.1038/s43587-023-00523-w.

[37] Sims JR, Zimmer JA, Evans CD, Lu M, Ardayfio P, Sparks J, et al. Donanemab in Early Symptomatic Alzheimer Disease. JAMA 2023;330:512. https://doi.org/10.1001/jama.2023.13239.

[38] Soininen H, West C, Robbins J, Niculescu L. Long-term efficacy and safety of celecoxib in Alzheimer’s disease. Dement Geriatr Cogn Disord 2006;23:8–21. https://doi.org/10.1159/000096588.

[39] Sparks DL, Sabbagh MN, Connor DJ, Lopez J, Launer LJ, Browne P, et al. Atorvastatin for the treatment of mild to moderate Alzheimer disease: preliminary  results. Arch Neurol 2005;62:753–7. https://doi.org/10.1001/archneur.62.5.753.

[40] Sperling R, Henley D, Aisen PS, Raman R, Donohue MC, Ernstrom K, et al. Findings of Efficacy, Safety, and Biomarker Outcomes of Atabecestat in Preclinical Alzheimer Disease: A Truncated Randomized Phase 2b/3 Clinical Trial. JAMA Neurol 2021;78:293–301. https://doi.org/10.1001/jamaneurol.2020.4857.

[41] Sur C, Kost J, Scott D, Adamczuk K, Fox NC, Cummings JL, et al. BACE inhibition causes rapid, regional, and non-progressive volume reduction in Alzheimer’s disease brain. Brain 2020;143:3816–26. https://doi.org/10.1093/brain/awaa332.

[42] Swanson CJ, Zhang Y, Dhadda S, Wang J, Kaplow J, Lai RYK, et al. A randomized, double-blind, phase 2b proof-of-concept clinical trial in early  Alzheimer’s disease with lecanemab, an anti-Aβ protofibril antibody. Alzheimers Res Ther 2021;13:80. https://doi.org/10.1186/s13195-021-00813-8.

[43] Tariot PN, Solomon PR, Morris JC, Kershaw P, Lilienfeld S, Ding C. A 5-month, randomized, placebo-controlled trial of galantamine in AD. Neurology 2000;54:2269–76. https://doi.org/10.1212/WNL.54.12.2269.

[44] Teng E, Manser PT, Pickthorn K, Brunstein F, Blendstrup M, Sanabria Bohorquez S, et al. Safety and Efficacy of Semorinemab in Individuals With Prodromal to Mild Alzheimer Disease. JAMA Neurol 2022;79:758–67. https://doi.org/10.1001/jamaneurol.2022.1375.

[45] Thal LJ, Carta A, Clarke WR, Ferris SH, Friedland RP, Petersen RC, et al. A 1-year multicenter placebo-controlled study of acetyl-L-carnitine in patients with Alzheimer’s disease. Neurology 1996;47:705–11. https://doi.org/10.1212/wnl.47.3.705.

[46] Thal LJ, Ferguson JM, Mintzer J, Raskin A, Targum SD. A 24-week randomized trial of controlled-release physostigmine in patients with Alzheimer’s disease. Neurology 1999;52:1146–52. https://doi.org/10.1212/wnl.52.6.1146.

[47] Thal LJ, Grundman M, Berg J, Ernstrom K, Margolin R, Pfeiffer E, et al. Idebenone treatment fails to slow cognitive decline in Alzheimer’s disease. Neurology 2003;61:1498–502. https://doi.org/10.1212/01.WNL.0000096376.03678.C1.

[48] Thal LJ, Forrest M, Loft H, Mengel H. Lu 25-109, a muscarinic agonist, fails to improve cognition in Alzheimer’s disease. Neurology 2000;54:421–6. https://doi.org/10.1212/wnl.54.2.421.

[49] Thal LJ, Calvani M, Amato A, Carta A. A 1-year controlled trial of acetyl-L-carnitine in early-onset AD. Neurology 2000;55:805–10. https://doi.org/10.1212/WNL.55.6.805.

[50] Turner RS, Thomas RG, Craft S, Van Dyck CH, Mintzer J, Reynolds BA, et al. A randomized, double-blind, placebo-controlled trial of resveratrol for Alzheimer disease. Neurology 2015;85:1383–91. https://doi.org/10.1212/WNL.0000000000002035.

[51] van Dyck CH, Sadowsky C, Le Prince Leterme G, Booth K, Peng Y, Marek K, et al. Vanutide Cridificar (Acc-001) and Qs-21 Adjuvant in Individuals With Early Alzheimer’S Disease: Amyloid Imaging Positron Emission Tomography and Safety Results From a Phase 2 Study. J Prev Alzheimers Dis 2016;3:1–10. https://doi.org/10.14283/jpad.2016.91.

[52] van Dyck CH, Swanson CJ, Aisen P, Bateman RJ, Chen C, Gee M, et al. Lecanemab in early Alzheimer’s disease. N Engl J Med 2022;388:9–21. https://doi.org/10.1056/NEJMoa2212948.

[53] vTv Therapeutics. Evaluation of the Efficacy and Safety of Azeliragon (TTP488) in Patients With Mild Alzheimer’s Disease (STEADFAST). ClinicalTrialsGov n.d.

[54] Vandenberghe R, Rinne JO, Boada M, Katayama S, Scheltens P, Vellas B, et al. Bapineuzumab for mild to moderate Alzheimer’s disease in two global, randomized, phase 3 trials. Alzheimers Res Ther 2016;8:18. https://doi.org/10.1186/s13195-016-0189-7.

[55] Wessels AM, Tariot PN, Zimmer JA, Selzler KJ, Bragg SM, Andersen SW, et al. Efficacy and Safety of Lanabecestat for Treatment of Early and Mild Alzheimer Disease: The AMARANTH and DAYBREAK-ALZ Randomized Clinical Trials. JAMA Neurol 2020;77:199–209. https://doi.org/10.1001/jamaneurol.2019.3988.

[56] Wilcock GK, Black SE, Hendrix SB, Zavitz KH, Swabb EA, Laughlin MA. Efficacy and safety of tarenflurbil in mild to moderate Alzheimer’s disease: a randomised phase II trial. Lancet Neurol 2008;7:483–93. https://doi.org/10.1016/S1474-4422(08)70090-5.

[57] Wilcock GK, Lilienfeld S, Gaens E. Efficacy and safety of galantamine in patients with mild to moderate Alzheimer’s disease: Multicentre randomised controlled trial. Br Med J 2000;321:1445–9. https://doi.org/10.1136/bmj.321.7274.1445.

**Supplementary Table 1**. Missing data for baseline covariates and longitudinal outcomes (ADAS-Cog_13_ and Whole Brain Volume) through Week 78 in HMTM (ITT and MCI subpopulation) and CPAD

|  | **CPAD Placebo (N = 866)** | | **HMTM** | | | |
| --- | --- | --- | --- | --- | --- | --- |
|  |  |  | **ITT (N = 252)** | | **MCI Subgroup (N = 105)** | |
| **Variable** | **N missing** | **% missing** | **N missing** | **% missing** | **N missing** | **% missing** |
| **Baseline ADAS-Cog_13_** | 51 | 5.9 | 0 | 0 | 0 | 0 |
| **Baseline WBV** | 28 | 3.2 | 1 | 0.4 | 0 | 0 |
| **ApoE4** | 24 | 2.8 | 27 | 10.7 | 8 | 7.6 |
| **AGE** | 0 | 0 | 0 | 0 | 0 | 0 |
| **SEX** | 0 | 0 | 0 | 0 | 0 | 0 |
| **Post Secondary Education** | 0 | 0 | 1 | 0.4 | 0 | 0 |
| **Smoking** | 0 | 0 | 0 | 0 | 0 | 0 |
| **Baseline CDR** | 0 | 0 | 0 | 0 | 0 | 0 |
| **Baseline MMSE** | 0 | 0 | 0 | 0 | 0 | 0 |
| **Change in ADAS-cog_13_ Week 78** | 209 | 24.1 | 75 | 29.8 | 29 | 27.6 |
| **Change in ADAS-cog_13_ Week 52** | 121 | 14.0 | 54 | 21.4 | 21 | 20.0 |
| **Change in ADAS-cog_13_ Week 39** | - | - | 40 | 15.9 | 14 | 13.3 |
| **Change in ADAS-cog_13_ Week 26** | 117 | 13.5 | 25 | 9.9 | 9 | 8.6 |
| **Change in ADAS-cog_13_ Week 13** | - | - | 11 | 4.4 | 4 | 3.8 |
| **Change in WBV Week 78** | 262 | 30.3 | 89 | 35.3 | 34 | 32.4 |
| **Change in WBV Week 52** | 825 | 95.3 | 82 | 32.5 | 31 | 29.5 |
| **Change in WBV Week 39** | - | - | 49 | 19.4 | 16 | 15.2 |
| **Change in WBV Week 26** | 855 | 98.7 | 36 | 14.3 | 15 | 14.3 |

**Supplementary Table 2**. Demographic and baseline clinical characteristics for the Week 78 outcome-specific analysis subsets (ADAS-Cog_13_, n=112; WBV, n=99), including standardised mean differences (SMDs).

|  | **ADAS-cog_13_** | | | |  | **WBV** | | |
| --- | --- | --- | --- | --- | --- | --- | --- | --- |
|  | **HMTM 16mg/day**  **N=112** | | **Placebo**  **N=112** | **SMD** |  | **HMTM 16mg/day**  **N=99** | **Placebo**  **N=99** | **SMD** |
| **Age (years)** | 71.8 (8.5) | | 71.5 (8.0) | 0.036 |  | 71.0 (8.7) | 71.3 (7.7) | -0.044 |
| **Sex** |  | |  |  |  |  |  |  |
| F | 77 (69%) | | 77 (69%) | 0 |  | 71 (72%) | 66 (67%) | 0.051 |
| M | 35 (31%) | | 35 (31%) | 0 |  | 28 (28%) | 33 (33%) | -0.051 |
| **Post-secondary Education** | 57 (51%) | | 53 (47%) | 0.036 |  | 46 (46%) | 43 (43%) | 0.03 |
| **Smoking** | 27 (24%) | | 24 (21%) | 0.027 |  | 24 (24%) | 23 (23%) | 0.01 |
| **ApoE4** | 60 (54%) | | 48 (43%) | 0.107 |  | 50 (51%) | 41 (41%) | 0.091 |
| **CDR** |  | |  |  |  |  |  |  |
| Questionable impairment (0.5) | 63 (56%) | | 62 (55%) | 0.009 |  | 58 (59%) | 58 (59%) | 0 |
| Mild impairment (1) | 45 (40%) | | 47 (42%) | -0.018 |  | 37 (37%) | 40 (40%) | -0.03 |
| Moderate impairment (2) | 4 (3.6%) | | 3 (2.7%) | 0.009 |  | 4 (4.0%) | 1 (1.0%) | 0.03 |
| **MMSE** |  | |  |  |  |  |  |  |
| Very mild | 21 (19%) | | 21 (19%) | 0 |  | 18 (18%) | 21 (21%) | -0.03 |
| Mild | 57 (51%) | | 66 (59%) | -0.08 |  | 48 (48%) | 53 (54%) | -0.051 |
| Moderate | 34 (30%) | | 25 (22%) | 0.08 |  | 33 (33%) | 25 (25%) | 0.081 |
| **ADAS-Cog_13_** | 27.9 (9.8) | | 29.9 (7.7) | -0.227 |  | - | - | - |
| **Whole Brain Volume (cm³)** | | - | - | - |  | 978.1 (103.8) | 967.8 (101.0) | 0.101 |
| Unknown | - | | - | - |  | 0 | 1 |  |
| **Logit Propensity Score** | -1.2 (1.2) | | -1.2 (1.2) | 0.01 |  | -1.1 (1.2) | -1.1 (1.2) | 0.01 |

**Supplementary Table 3.** Demographic and baseline clinical characteristics for the Week 78 outcome-specific analysis subsets (ADAS-Cog_13_, n=49; WBV, n=44), including standardised mean differences (SMDs) in MCI-AD subpopulation.

|  | **ADAS-cog_13_** | | | |  | **WBV** | | |
| --- | --- | --- | --- | --- | --- | --- | --- | --- |
|  | **HMTM 16mg/day**  **N=49** | | **Placebo**  **N=49** | **SMD** |  | **HMTM 16mg/day**  **N=44** | **Placebo**  **N=44** | **SMD** |
| **Age (years)** | 70.7 (7.2) | | 71.6 (7.1) | -0.137 |  | 70.1 (7.5) | 71.2 (7.4) | -0.153 |
| **Sex** |  | |  |  |  |  |  |  |
| F | 34 (69%) | | 32 (65%) | 0.041 |  | 32 (73%) | 29 (66%) | 0.068 |
| M | 15 (31%) | | 17 (35%) | -0.041 |  | 12 (27%) | 15 (34%) | -0.068 |
| **Post-secondary Education** | 27 (55%) | | 23 (47%) | 0.082 |  | 23 (52%) | 18 (41%) | 0.114 |
| **Smoking** | 13 (27%) | | 8 (16%) | 0.102 |  | 10 (23%) | 6 (14%) | 0.091 |
| **ApoE4** | 29 (59%) | | 23 (47%) | 0.122 |  | 23 (52%) | 19 (43%) | 0.091 |
| **CDR** |  | |  |  |  |  |  |  |
| Questionable impairment (0.5) | 43 (88%) | | 26 (53%) | 0.347 |  | 40 (91%) | 24 (55%) | -0.364 |
| Mild impairment (1) | 6 (12%) | | 22 (45%) | -0.327 |  | 4 (9.1%) | 20 (45%) | 0.136 |
| Moderate impairment (2) | 0 (0%) | | 1 (2.0%) | -0.02 |  | 0 (0%) | 0 (0%) | -0.023 |
| **MMSE** |  | |  |  |  |  |  |  |
| Very mild | 20 (41%) | | 13 (27%) | 0.143 |  | 18 (41%) | 12 (27%) | -0.114 |
| Mild | 24 (49%) | | 25 (51%) | -0.02 |  | 21 (48%) | 22 (50%) | 0.252 |
| Moderate | 5 (10%) | | 11 (22%) | -0.122 |  | 5 (11%) | 10 (23%) | 0.009 |
| **ADAS-Cog_13_** | 23.2 (8.6) | | 29.2 (8.0) | -0.724 |  | - | - | - |
| **Whole Brain Volume (cm³)** | | - | - | - |  | 999.6 (92.9) | 975.0 (102.2) | NA |
| **Logit Propensity Score** | -1.3 (1.2) | | -1.3 (1.2) | 0.01 |  | -1.3 (1.1) | -1.3 (1.1) | NA |

**Supplementary Table 4**. Demographic and baseline clinical characteristics for the Week 78 outcome-specific analysis subsets (ADAS-Cog_13_, n=63; WBV, n=55), including standardised mean differences (SMDs) in AD subpopulation.

|  | **ADAS-cog_13_** | | | |  | **WBV** | | |
| --- | --- | --- | --- | --- | --- | --- | --- | --- |
|  | **HMTM 16mg/day**  **N=63** | | **Placebo**  **N=63** | **SMD** |  | **HMTM 16mg/day**  **N=55** | **Placebo**  **N=55** | **SMD** |
| **Age (years)** | 72.6 (9.4) | | 71.3 (8.7) | 0.142 |  | 71.7 (9.5) | 71.4 (8.1) | 0.029 |
| **Sex** |  | |  |  |  |  |  |  |
| F | 43 (68%) | | 45 (71%) | -0.032 |  | 39 (71%) | 37 (67%) | 0.036 |
| M | 20 (32%) | | 18 (29%) | 0.032 |  | 16 (29%) | 18 (33%) | -0.036 |
| **Post-secondary Education** | 30 (48%) | | 30 (48%) | 0 |  | 23 (42%) | 25 (45%) | -0.036 |
| **Smoking** | 14 (22%) | | 16 (25%) | -0.032 |  | 14 (25%) | 17 (31%) | -0.055 |
| **ApoE4** | 31 (49%) | | 25 (40%) | 0.095 |  | 27 (49%) | 22 (40%) | 0.091 |
| **CDR** |  | |  |  |  |  |  |  |
| Questionable impairment (0.5) | 20 (32%) | | 36 (57%) | -0.254 |  | 18 (33%) | 34 (62%) | -0.291 |
| Mild impairment (1) | 39 (62%) | | 25 (40%) | 0.222 |  | 33 (60%) | 20 (36%) | 0.236 |
| Moderate impairment (2) | 4 (6.3%) | | 2 (3.2%) | 0.032 |  | 4 (7.3%) | 1 (1.8%) | 0.055 |
| **MMSE** |  | |  |  |  |  |  |  |
| Very mild | 1 (1.6%) | | 8 (13%) | -0.111 |  | 0 (0%) | 9 (16%) | -0.164 |
| Mild | 33 (52%) | | 41 (65%) | -0.127 |  | 27 (49%) | 31 (56%) | -0.073 |
| Moderate | 29 (46%) | | 14 (22%) | 0.238 |  | 28 (51%) | 15 (27%) | 0.236 |
| **ADAS-Cog_13_** | 31.6 (9.2) | | 30.5 (7.5) | 0.131 |  | - | - | - |
| **Whole Brain Volume (cm³)** | | - | - | - |  | 960.9 (109.6) | 961.9 (100.6) | -0.009 |
| Unknown | - | | - | - |  | 0 | 1 |  |
| **Logit Propensity Score** | -1.1 (1.2) | | -1.1 (1.2) | 0.009 |  | -0.9 (1.3) | -0.9 (1.3) | 0.011 |

**Supplementary Table 5** CPAD analysis: Odds-ratio of progression on the CDR-global scale at 104 weeks

| **Populations** | **Time Point (weeks)** | **No. of Participants Evaluated** |  | **TRx-237-039 HMTM 16mg/day vs Matched Placebo** | | |
| --- | --- | --- | --- | --- | --- | --- |
|  |  |  |  | **Odds Ratio (95% CI)** |  | **p-value (2-sided)** |
| **Whole population** | 104 | 42 |  | 0·31 (0·137, 0·707) |  | 0·005 |
| **MCI only** | 104 | 19 |  | 0·15 (0·039, 0·579) |  | 0·006 |
| **AD only** | 104 | 23 |  | 0·51 (0·177, 1·50) |  | 0·223 |

**Supplementary Table 6.** Demographic and Baseline Clinical Characteristics before and after Matching for Subjects Not Receiving Cholinesterase Inhibitors or Memantine in the CPAD database.

|  | **Pre-matching** | | **Post-matching** | |
| --- | --- | --- | --- | --- |
| **Characteristic** | **CPAD Placebo**  **N = 632** | **TRx-237-080 HMTM 16mg/day**  **N = 169** | **CPAD Placebo**  **N = 106** | **TRx-237-080 HMTM 16 mg/day**  **N = 106** |
| **Age (years)** | 74 (68, 78) | 73 (66, 78) | 73 (67, 78) | 73 (66, 78) |
| **Sex** |  |  |  |  |
| F | 384 (61%) | 109 (64%) | 72 (68%) | 73 (69%) |
| M | 248 (39%) | 60 (36%) | 34 (32%) | 33 (31%) |
| ***APOE4*** | 425 (67%) | 77 (46%) | 55 (52%) | 54 (51%) |
| **Post-secondary Education** | 376 (59%) | 73 (43%) | 52 (49%) | 53 (50%) |
| **Smoking** | 32 (5.1%) | 71 (42%) | 25 (24%) | 23 (22%) |
| **Baseline CDR** |  |  |  |  |
| Mild impairment (1) | 203 (32%) | 70 (41%) | 40 (38%) | 38 (36%) |
| Moderate impairment (2) | 10 (1.6%) | 8 (4.7%) | 5 (4.7%) | 6 (5.7%) |
| Questionable impairment (0.5) | 419 (66%) | 91 (54%) | 61 (58%) | 62 (58%) |
| **Baseline MMSE** |  |  |  |  |
| Mild | 434 (69%) | 79 (47%) | 56 (53%) | 57 (54%) |
| Moderate | 95 (15%) | 57 (34%) | 30 (28%) | 27 (25%) |
| Very mild | 103 (16%) | 33 (20%) | 20 (19%) | 22 (21%) |
| **Prior AChMem usage** | 521 (82%) | 57 (34%) | 53 (50%) | 51 (48%) |
| **Baseline MMSE** | 23 (20.75, 25) | 22 (19, 25) | 22 (19.25, 25) | 22 (19.25, 24.75) |
| **Baseline ADAS-Cog_13_** | 29 (24, 34) | 27 (20, 33) | 30 (25, 35) | 27 (20, 33) |
| **Baseline ADAS-Cog_11_** | 18 (14, 22) | 17 (13, 23) | 18 (15, 22) | 17 (14, 23) |
| **Baseline WBV (cm^3^)** | 964.44 (898.93, 1033.72) | 974.52 (912.14, 1048.68) | 983.94 (912.58, 1035.29) | 981.93 (917.42, 1044.96) |
| **Logit Propensity Score** |  |  | -0.86 (-1.42, -0.06) | -0.86 (-1.39, -0.03) |

**Supplementary Table 7.** Sensitivity Analysis Comparing HMTM 16 mg/day with Subjects not Receiving Cholinesterase Inhibitors or Memantine in the CPAD Database overall and split by Baseline Diagnosis as MCI or mild/moderate AD.

| **Outcomes** | **Time Point (weeks)** | **No. of Subjects Evaluated**  **N** | **TRx-237-080 HMTM 16mg/day**  **Mean ± SE** | **Matched Placebo**  **Mean ± SE** | **Difference**  **(95% CI)** | **TRx-237-080 HMTM 16mg/day vs Matched Placebo**  **p-value (2-sided)** |
| --- | --- | --- | --- | --- | --- | --- |
| **Whole population** | | | | | | |
| ADAS-cog_13_ | 52 | 97 | -0.432 ± 0.849 | 4.526 ± 0.849 | -4.958 (-7.327, -2.590) | <0.0001 |
|  | 78 | 94 | -0.361 ± 0.950 | 5.340 ± 0.950 | -5.702 (-8.351, -3.052) | <0.0001 |
|  | 104 | 82 | 1.285 ± 1.091 | 8.171 ± 1.091 | -6.886 (-9.934, -3.838) | <0.0001 |
| WBV (cm^3^) | 78 | 77 | -15.66 ± 1.50 | -22.09 ± 1.50 | 6.428 (2.229, 10.627) | 0.003 |
|  | 104 | 22 | -18.31 ± 3.24 | -27.06 ± 3.24 | 8.745 (-0.496, 17.986) | 0.063 |
| **MCI only** | | | | | | |
| ADAS-cog_13_ | 52 | 46 | -2.942 ± 7.845 | 4.196 ± 7.580 | -7.138 (-10.333, -3.943) | <0.0001 |
|  | 78 | 45 | -3.060 ± 1.217 | 5.178 ± 1.217 | -8.237 (-11.657, -4.818) | <0.0001 |
|  | 104 | 39 | 0.872 ± 1.499 | 8.308 ± 1.499 | -7.436 (-11.657, -3.215) | <0.001 |
| WBV (cm^3^) | 78 | 36 | -11.064 ± 1.963 | -22.057 ± 1.963 | 10.993 (5.456, 16.530) | <0.001 |
|  | 104 | 11 | -18.680 ± 4.587 | -21.707 ± 4.587 | 3.026 (-10.505, 16.558) | 0.646 |
| **Mild to Moderate AD only** | | | | | | |
| ADAS-cog_13_ | 52 | 51 | 1.831 ± 1.214 | 4.824 ± 1.214 | -2.992 (-6.398, 0.413) | 0.084 |
|  | 78 | 49 | 2.117 ± 1.404 | 5.490 ± 1.404 | -3.373 (-7.314, 0.568) | 0.093 |
|  | 104 | 43 | 1.659 ± 1.592 | 8.047 ± 1.592 | -6.387 (-10.864, -1.911) | 0.006 |
| WBV (cm^3^) | 78 | 41 | -19.704 ± 2.159 | -22.126 ± 2.159 | 2.421 (-3.656, 8.498) | 0.430 |
|  | 104 | 11 | -17.942 ± 4.485 | -32.407 ± 4.485 | 14.464 (1.233, 27.696) | 0.034 |

**Supplementary Table 8:** Estimates for ADAS-cog_13_ and WBV at Week 78 - IPW with PS estimated by CBPS, weights targeting average treatment effect (ATE)

|  | **Missing Outcome Values Imputation Method** | | **Estimate** | **CI (2.5%)** | **CI (97.5%)** | **SE** | **p-value**  **(two-sided)** |
| --- | --- | --- | --- | --- | --- | --- | --- |
| **MCI&AD** | |  |  |  |  |  |  |
| **ADAS-cog_13_** | MAR | | -5.24 | -7.33 | -3.16 | 1.064 | <0.0001 |
|  | Copy-Reference | | -3.99 | -6.06 | -1.93 | 1.053 | 0.0002 |
|  | Copy-Increment-Reference | | -4.02 | -6.08 | -1.95 | 1.054 | 0.0001 |
|  | Jump-to-Reference | | -3.19 | -5.31 | -1.07 | 1.082 | 0.0032 |
| **WBV (cm^3^)** | MAR | | 6.61 | 4.05 | 9.17 | 1.305 | <0.0001 |
|  | Copy-Reference | | 4.93 | 2.27 | 7.60 | 1.358 | 0.0003 |
|  | Jump-to-Reference | | 4.97 | 2.31 | 7.63 | 1.357 | 0.0003 |
| **MCI-only** | |  |  |  |  |  |  |
| **ADAS-cog_13_** | MAR | | -8.97 | -12.34 | -5.60 | 1.719 | <0.0001 |
|  | Copy-Reference | | -7.27 | -10.81 | -3.73 | 1.806 | <0.0001 |
|  | Copy-Increment-Reference | | -7.36 | -10.81 | -3.83 | 1.800 | <0.0001 |
|  | Jump-to-Reference | | -5.95 | -9.77 | -2.13 | 1.947 | 0.0023 |
| **WBV (cm^3^)** | MAR | | 11.26 | 7.56 | 14.95 | 1.880 | <0.0001 |
|  | Copy-Reference | | 7.92 | 3.77 | 12.08 | 2.115 | 0.0002 |
|  | Jump-to-Reference | | 8.07 | 3.94 | 12.21 | 2.105 | 0.0001 |

**Supplementary Table 9:** Analysis of unmeasured confounding in the most conservative treatment effect estimates from the three IPW methods

|  |  | **Analysis** | **Estimate** | **SE** | **Cohen’s d** | **E-value** |
| --- | --- | --- | --- | --- | --- | --- |
| **MCI&AD** |  |  |  |  |  |  |
| ADAS-cog_13_ |  | MAR Analysis | -5.242 | 1.064 | 0.582 | 2.788 |
|  |  | CR Imputation | -3.991 | 1.053 | 0.443 | 2.359 |
|  |  | JR Imputation | -3.192 | 1.082 | 0.355 | 2.106 |
| WBV |  | MAR Analysis | 6.610 | 1.305 | 0.528 | 2.615 |
|  |  | JR Imputation | 4.773 | 1.623 | 0.381 | 2.180 |
|  |  | CR Imputation | 4.738 | 1.625 | 0.378 | 2.172 |
| **MCI-only** |  |  |  |  |  |  |
| ADAS-cog_13_ |  | MAR Analysis | -8.967 | 1.744 | 1.017 | 4.484 |
|  |  | CR Imputation | -7.269 | 1.806 | 0.828 | 3.671 |
|  |  | JR Imputation | -5.946 | 1.947 | 0.678 | 3.110 |
| WBV |  | MAR Analysis | 11.258 | 1.880 | 0.929 | 4.086 |
|  |  | JR Imputation | 8.070 | 2.105 | 0.666 | 3.068 |
|  |  | CR Imputation | 7.922 | 2.115 | 0.653 | 3.026 |

**Supplementary Table 10**. Demographic and Baseline Clinical Characteristics before and after Matching with ADNI Database

| **Characteristic** | **Unmatched patient population** | | **Matched patient population** | |
| --- | --- | --- | --- | --- |
|  | **ADNI (N=322)** | **TRx-237-039 (N=431)** | **ADNI (N=189)** | **TRx-237-039 (N=189)** |
| Age – mean (SD) | 74.7 (7.2) | 71.8 (8.6) | 73.2 (7.3) | 72.7 (8.0) |
| Sex – n (%) |  |  |  |  |
| Female | 133 (41.3%) | 264 (61.3%) | 97 (51.3%) | 95 (50.3%) |
| Male | 189 (58.7%) | 167 (38.7%) | 92 (48.7%) | 94 (49.7%) |
| Current or past smoker – n (%) | 131 (40.7%) | 170 (39.4%) | 75 (39.7%) | 75 (39.7%) |
| High school education or less – n (%) | 69 (21.4%) | 232 (53.8%) | 62 (32.8%) | 55 (29.1%) |
| Clinical subgroup – n (%) |  |  |  |  |
| MCI-AD | 254 (78.9%) | 198 (45.9%) | 144 (76.2%) | 121 (64.0%) |
| AD | 68 (21.1%) | 233 (54.1%) | 45 (23.8%) | 68 (36.0%) |
| *APOε4* status – n (%) |  |  |  |  |
| Noncarrier | 131 (40.7%) | 216 (50.1%) | 79 (41.8%) | 86 (45.5%) |
| Carrier | 191 (59.3%) | 215 (49.9%) | 110 (58.2%) | 103 (54.5%) |
| MMSE score – n (%) |  |  |  |  |
| 16-19 | 1 (0.3%) | 152 (35.3%) | 1 (0.5%) | 1 (0.5%) |
| 20-25 | 147 (45.7%) | 201 (46.6%) | 110 (58.2%) | 115 (60.8%) |
| 26-27 | 174 (54.0%) | 78 (18.1%) | 78 (41.3%) | 73 (38.6%) |
| MMSE – mean (SD) | 25.2 (1.8) | 21.6 (3.3) | 24.9 (1.9) | 24.0 (2.4%) |
| CDR score – n (%) |  |  |  |  |
| 0.5 | 286 (88.8%) | 240 (55.7%) | 165 (87.3%) | 133 (70.4%) |
| 1 | 36 (11.2%) | 172 (39.9%) | 24 (12.7%) | 56 (29.6%) |
| 2 | 0 | 19 (4.4) | 0 | 0 |
| ADAS-cog_11_ – mean (SD) | 12.9 (5.3) | 18.4 (7.9) | 13.0 (5.2) | 15.3 (6.2) |
| ADAS-cog_13_ – mean (SD) | 20.8 (7.6) | 27.4 (10.4) | 20.9 (7.5) | 23.4 (8.5) |
| Whole brain volume (cm^3^) – mean (SD) | 1009.8 (117.6) | 978.4 (108.9) | 998.7 (124.0) | 1002.0 (109.6) |

**Supplementary Table 11.** Comparisons of Matched TRx-237-039 and ADNI Populations at 52 and 104-weeks in the whole population and MCI subpopulation

| **Outcomes** |  | **Time Point** | | |  | **No. of Participants Evaluated** |  | **TRx-237-039 HMTM 16mg/day** |  | **ADNI** |  | **Difference** |  | **p-value** |
| --- | --- | --- | --- | --- | --- | --- | --- | --- | --- | --- | --- | --- | --- | --- |
|  |  | **(weeks)** | | |  | **N** |  | **Mean ± SD** |  | **Mean ± SD** |  | **(95% CI)** |  |  |
| **Whole population** | | | |  | | | | | | | | | | |
| ADAS-cog_11_ |  | 52 | | |  | 184 |  | 0.77±6.74 |  | 2.77±6.53 |  | 2.00 (-3.36, -0.64) |  | 0.004 |
|  |  | 104 | | |  | 110 |  | 2.28±7.63 |  | 4.48±7.75 |  | -2.20 (-4.25, -0.16) |  | 0.035 |
| WBV (cm^3^) |  | 52 | | |  | 127 |  | -8.61±10.18 |  | -13.40±10.19 |  | 4.79 (2.27, 7.31) |  | <0.001 |
|  |  | 104 | | |  | 68 |  | -17.02±13.95 |  | -24.05±13.77 |  | 7.03 (2.33, 11.73) |  | 0.004 |
| **MCI only** | | |  | | | | | | | | | | | |
| ADAS-cog_13_ |  | 52 | | |  | 46 |  | -2.22±5.85 |  | 1.76±4.22 |  | -3.99 (-6.10, -1.87) |  | <0.001 |
|  |  | 104 | | |  | 34 |  | 0.33±7.69 |  | 2.95±4.82 |  | -2.62 (-5.72, 0.49) |  | 0.097 |
| WBV (cm^3^) |  | 52 | | |  | 35 |  | -4.65±7.85 |  | -10.88±9.77 |  | 6.23 (2.00, 10.45) |  | 0.004 |
|  |  | 104 | | |  | 22 |  | -10.15±13.34 |  | -22.88±12.11 |  | 12.73 (4.98, 20.48) |  | 0.002 |

**Supplementary Table 12.** Comparison of Participants Receiving HMTM 16 mg/day with Meta-analysis of Placebo Arms from Trials in Comparable Populations over 78-weeks.

| **Outcome** | **Time Point**  **(Weeks)** | **Number Subjects (Number Trials) for TauRx / Meta** | **TRx-237-039**  **HMTM 16mg/day**  **Mean ± SE** | **Meta-analytic control**  **Mean ± SE** | **Delta**  **(95% CI)** | **p-value**  **(2-sided)** |
| --- | --- | --- | --- | --- | --- | --- |
| ADAS-cog_13_ | 24 | 218 (1) / 8567 (36) | -0.788 ± 0.463 | 1.480 ± 0.153 | -2.3 (-3.224, -1.312) | 3.302x10^-6^ |
|  | 52 | 180 (1) / 7914 (32) | 0.524 ± 0.663 | 4.109 ± 0.513 | -3.6 (-5.228, -1.942) | 1.899x10^-5^ |
|  | 78 | 157 (1) / 5116 (20) | 1.017 ± 0.793 | 7.024 ± 0.360 | -6.0 (-7.714, -4.300) | 5.305x10^-12^ |
| ADCS-ADL_23_ | 24 | 220 (1) / 6657 (19) | -0.069 ± 0.666 | -1.901 ± 0.226 | 1.8 (0.454, 3.209) | 0.0092 |
|  | 52 | 193 (1) / 8484 (26) | -1.068 ± 0.897 | -4.605 ± 0.437 | 3.5 (1.582, 5.494) | 0.0004 |
|  | 78 | 172 (1) / 6348 (21) | -1.827 ± 1.117 | -7.834 ± 0.571 | 6.0 (3.547, 8.467) | 1.695x10^-6^ |
| CDR-SoB | 24 | -/ 1805 (9) | - | 0.633 ±0.086 | - | - |
|  | 52 | 193 (1) / 4729 (21) | 0.710 ± 0.204 | 1.548 ± 0.090 | -0.8 (-1.275, -0.401) | 0.0002 |
|  | 78 | - /7643 (24) | - | 2.285 ±0.164 | - | - |
| WBV | 24 | 209 (1) / 2643 (10) | -6.087 ± 0.587 | -8.197 ± 0.820 | 2.1 (0.134, 4.086) | 0.0364 |
|  | 52 | 176 (1) /3360 (15) | -10.450 ± 0.837 | -15.395 ± 0.945 | 4.9 (2.470, 7.421) | 9.01x10^-5^ |
|  | 78 | 157 (1) / 3944 (15) | -15.620 ± 1.141 | -22.454 ± 1.037 | 6.8 (3.809, 9.854) | 9.40x10^-6^ |

**Supplementary Table 13:** Meta-analysis Restricted to Randomized Controlled Trials Reporting Identical Endpoint Definitions: Comparison of HMTM 16 mg/day with Matched 78-Week Placebo Arms

| **Outcome** | **Time Point**  **(months)** | **Delta**  **(TauRx – Meta-analytic Control)** | **p-value**  **(2-sided)** |
| --- | --- | --- | --- |
| ADAS-cog_13_ | 12 | -2.6 | 0.0004 |
|  | 18 | -4.3 | <0.0001 |
| WBV | 12 | 1.8 | 0.38 |
|  | 18 | 6.6 | <0.0001 |

**Supplementary Figure 1**: TRx-237-039 and ADNI inclusion/exclusion criteria flowchart

**Supplementary Figure 2.** MAR analysis: ADAS-Cog_13_ at Week 78 in the ITT population, covariate balance and weight distributions using logistic regression, BART, and CBPS weights

**Supplementary Figure 3**. NMAR analysis: ADAS-Cog_13_ at Week 78 in the ITT population, covariate balance and weight distributions using logistic regression, BART, and CBPS weights

**Supplementary Figure 4.** MAR analysis: Whole Brain Volume at Week 78 in the ITT population, covariate balance and weight distributions using logistic regression, BART, and CBPS weights

**Supplementary Figure 5.** NMAR analysis: Whole Brain Volume at Week 78 in the ITT population, covariate balance and weight distributions using logistic regression, BART, and CBPS weights

**Supplementary Figure 6**: Statistical parametric mapping analysis comparing differences in change in GM volume over 52 and 104 weeks in subjects receiving HMTM (8mg/day or 16 mg/day) vs ADNI. The longitudinal comparison was done using the CAT 12 longitudinal software (https://neuro-jena.github.io) [1]. The processing and modelling accounted for any variation in total intracranial volume caused by machine drift over time.

|  | **52-Weeks** | **104-Weeks** |
| --- | --- | --- |
| **HMTM (16mg/day & 8mg/day) vs ADNI** | 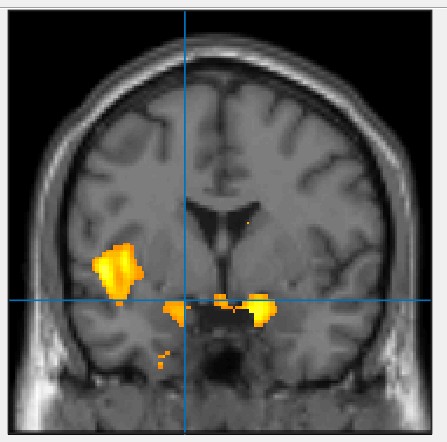 | 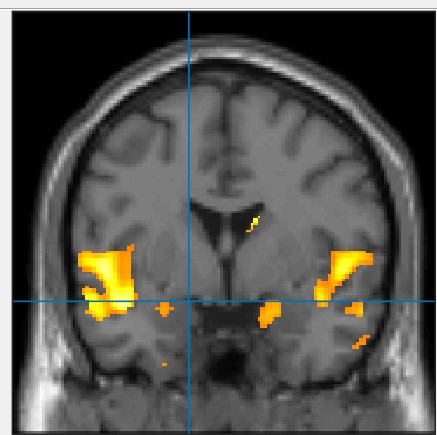 |

**Supplementary Figure 7:** Forest plots of treatment effects in WBV for HMTM 16 mg/day versus external placebo controls at Week 78 in the whole study population and MCI-AD subpopulation.

**References**

[1] Gaser C, Dahnke R, Thompson PM, Kurth F, Luders E, the Alzheimer’s Disease Neuroimaging Initiative. CAT: a computational anatomy toolbox for the analysis of structural MRI data. Gigascience 2024;13. https://doi.org/10.1093/gigascience/giae049.
