# Supplementary material for: Assessment of clinical and neuroimaging efficacy of treatment targeting tau pathology in mild cognitive impairment and mild to moderate Alzheimer’s disease with hydromethylthionine mesylate using external control data
Source: J Prev Alzheimers Dis. 2026 Apr 17;13(6):100560. doi: 10.1016/j.tjpad.2026.100560 (PMC13098421; doi:10.1016/j.tjpad.2026.100560)
Supplement: Supplementary file 2 [file mmc2.pdf]

CLINICAL STUDY PROTOCOL

The Comparative Effectiveness of Hydromethylthionine Mesylate (HMTM) Monotherapy in Subjects with Alzheimer's Disease versus a CPAD cohort from placebo arms of past clinical trials based on propensity score matching.

STUDY CODE: TRx-237-080

STUDY PHASE: Comparative analysis with external placebo trial data

VERSION 1.0 DATED 14 November 2024

TauRx Therapeutics Ltd.
3 Shenton Way, #21-04
Shenton House
Singapore 068805
Republic of Singapore

Operational Location:
395 King Street
Aberdeen AB24 5RP
Scotland, UK
Tel: +44 (0) 1224 440905

THIS PROTOCOL IS A CONFIDENTIAL DOCUMENT

This protocol is the property of TauRx Therapeutics Limited. The information within it is confidential and is provided to you, for review by you, your staff and applicable Ethics Committees and Institutional Review Boards. The protocol must be kept in a confidential manner and must be returned to TauRx upon request. No part of this document may be reproduced in any form without permission from TauRx. By accepting this document, you agree that the information contained therein will not be disclosed to a third party without written authorization from TauRx.

TABLE OF CONTENTS **PAGE**

1 TABLE OF CONTENTS 2

2 GCP COMPLIANCE STATEMENT 3

3 PROTOCOL APPROVAL 4

4 RESPONSIBLE PERSONNEL 5

5 SYNOPSIS 6

6 ABBREVIATIONS 10

7 BACKGROUND AND RATIONALE FOR THE STUDY 12

7.1 Background 13

7.1.1 Investigational Product 13

7.1.2 Clinical Data 14

7.2 Rationale 17

8 OBJECTIVES 19

8.1 Primary Objectives 19

9 STUDY DESIGN 20

9.1 General Description 20

9.2 Study Population 20

10 PARTICIPANT ENROLLMENT 21

10.1 Inclusion Criteria 21

10.2 Exclusion Criteria 21

11 STUDY ASSESSMENTS 23

11.1 Assessment of Clinical Efficacy 23

11.2 Imaging Assessments and Procedures 23

11.2.1 Imaging Methods for Efficacy 23

12 STATISTICAL ANALYSIS 24

12.1 Efficacy Endpoints 24

12.1.1 Primary And Secondary Efficacy Endpoints 24

12.2 Number of Participants and Sample Size Calculation 24

12.3 Analysis Populations 24

12.4 Clinical Efficacy and Imaging Analysis 25

12.4.1 Hypothesis 25

12.4.2 Propensity score matching 25

12.4.3 Handling of Missing and Incomplete Data 26

12.5 Demographic and Baseline Characteristics 26

13 CONFIDENTIALITY AND DATA PROTECTION 27

14 STANDARD PROCEDURES 28

15 REFERENCES 29

1 GCP COMPLIANCE STATEMENT

This study will be conducted in compliance with the protocol, and Guidelines for Good Clinical Practice (GCP) E6(R2) (or ICH E6(R1).

2 PROTOCOL APPROVAL

**Sponsor Signatory**<br>[redacted]	[redacted]
	Signature Date 19 NOV 2024
**TauRx Medical Oversight**<br>[redacted]	[redacted]
	Signature Date 19 NOV 2024
**Statistician**<br>[redacted]	[redacted]
	Signature Date 19-NOV-2024

3 RESPONSIBLE PERSONNEL

**Statistics & Data Management**<br>Cytel Inc<br>Geneva Branch<br>Route de Prebois 20<br>1215 Geneva<br>Switzerland<br>Tel: +41 791021971	

4 SYNOPSIS

Name of Sponsor / Company: TauRx Therapeutics Ltd (TauRx)
Name of Finished Product: HMTM (TRx0237) Film-coated Tablets, 4 mg
Name of Active Ingredient (Drug Substance): Hydromethylthionine Mesylate
**Number and Title of Study:** TRx-237-080: The Comparative Effectiveness of Hydromethylthionine Mesylate (HMTM) Monotherapy in Subjects with Alzheimer's Disease versus a CPAD cohort from placebo arms of past clinical trials based on propensity score matching.

**Study Duration:**<br>A comparative analysis of 52-week, 78-week and 104-week timepoints for various clinical and imaging endpoints with external placebo trial data will be performed.	Phase of Development:<br>Comparative analysis

Objectives

The primary and secondary objectives of the study pertain to comparative analysis of TRx-237-039, listed below. They focus on mild cognitive impairment due to Alzheimer's Disease (MCI-AD) and probable AD (mild/moderate AD) and comparisons with historic trial control data. The analysis is pre-defined in the sense that CPAD data has not been accessed or seen by the Statistics provider Cytel or the Sponsor.

Primary-analysis:

1. To compare the HMTM dose of 16 mg/day from completed study TRx-237-039 with a matched historic control group from placebo arms of trials in the CPAD dataset on the following endpoints at 78 weeks:
   a. Alzheimer's Disease Assessment Scale, 13-item version (ADAS-cog_{13}) in all participants at a two-sided alpha of 0.05;
   b. Whole brain atrophy in all participants at a two-sided alpha of 0.05.

Both co-primary endpoints need to reach statistical significance. If there are insufficient ADAS-Cog_{13} data available in the CPAD dataset, ADAS-Cog_{12} or ADAS-Cog_{11} will be used depending on availability. ADAS-Cog_{11} will in any case provide a sensitivity analysis. Similarly, if WBV cannot be analyzed because of insufficient data, other brain volumes will be investigated instead, preference being given to temporo-parietal brain volume.

Additional secondary and exploratory analyses are described in the SAP. These include analyses of:

1. MMSE
2. Clinical Dementia Rating (CDR) and CDR sum of boxes
3. Neurofilament light chain (NfL) if available and if possible to linearly scale to make comparable

Study Design

Data analysis of HMTM 16mg/day arm data from TRx-237-039 using propensity score matched data from a CPAD cohort as an external control. The analysis is pre-specified in the sense that neither the Statistics provider Cytel nor the study Sponsor had access to the CPAD data.

Name of Sponsor / Company: TauRx Therapeutics Ltd (TauRx)

Name of Finished Product: HMTM (TRx0237) Film-coated Tablets, 4 mg

Name of Active Ingredient (Drug Substance): Hydromethylthionine Mesylate

Sample Size

The sample size is dictated by the availability of data from TRx-237-039 and CPAD. The SAP attached to this protocol states the criteria which are applied should the resulting data set be too small for the desired comparisons.

Participant Population

Inclusion Criteria:

The inclusion and exclusion criteria for TRx-237-039 are available in the protocol for this study. TRx-237-080 will use similar inclusion and exclusion criteria.

To create a group of patients from CPAD - who are similar to the patients in the HMTM 16 mg/day arm of TRx-237-080, the following inclusion and exclusion criteria from TRx-237-039 trial will be applied to "enroll" patients from CPAD database prior to propensity score matching:

Inclusion criteria:

1) Clinical diagnosis of probable AD or MCI due to AD (MCI-AD; MCI-AD is referred to as MCI in the sections below)
2) PET scan positive for amyloid in MCI-AD; PET scan positivity for amyloid in the CPAD population is not needed in mild/moderate AD as this severity level is sufficient to replace the need for a PET scan in identifying patients who are anticipated to decline clinically and is not generally used trials in mild/moderate AD. If data availability for PET scan in the CPAD cohort is limited, the main analysis will be conducted without this criterion, but a sensitivity analysis including this criterion will be performed
3) MMSE score of 16 – 27 at screening
4) Global CDR score of 0.5 to 2 at screening (if 0.5, including a score of > 0 in one of the functional domains: Community Affairs, Home and Hobbies, or Personal Care). If data availability for CDR in the CPAD cohort is limited, this criterion will be dropped
5) Outpatients below 90 years at screening (patients residing in hospitals or moderate to high dependency continuous care facilities are excluded)

Exclusion criteria:

1. Significant central nervous system (CNS) disorder other than probable AD or MCI-AD, *e.g.,* Lewy body dementia, Parkinson's disease, multiple sclerosis, progressive supranuclear palsy, hydrocephalus, Huntington's disease, any condition directly or indirectly caused by Transmissible Spongiform, Encephalopathy (TSE), Creutzfeldt-Jakob Disease (CJD), variant Creutzfeldt-Jakob Disease (vCJD), or new variant Creutzfeldt-Jakob Disease (nvCJD).

2. Significant neuroimaging findings e.g. significant intracranial focal or vascular pathology seen on brain MRI including but not limited to:
   - Large confluent white matter hyperintense lesions (*i.e.*, Fazekas score of 3)
   - Other focal brain lesions judged clinically relevant by the investigator
   - Evidence of a prior or current macrohemorrhage.
3. Mental Disorders such as
   - Current major depressive disorder (MDD)

Name of Sponsor / Company: TauRx Therapeutics Ltd (TauRx)
Name of Finished Product: HMTM (TRx0237) Film-coated Tablets, 4 mg
Name of Active Ingredient (Drug Substance): Hydromethylthionine Mesylate

- History of schizophrenia
- Other psychotic disease, bipolar disorder within past 5 years from the baseline

  Substance (including alcohol) related disorders within past 5 years from the baseline

4. History of any conditions that may be impacting cognitive function:
   - Cerebrovascular accident
   - Transient ischemic attack
   - Significant head injury, for example, associated loss of consciousness, skull fracture or persisting cognitive impairment
   - Other unexplained or recurrent loss of consciousness
5. Diagnosed epilepsy (a single prior seizure >6 months prior to Screening is considered acceptable)
6. Treatment currently or within 30 days before the baseline with:
   - Acetylcholinesterase inhibitor (AChEI) and/or memantine
   - Clozapine (other antipsychotics are allowed if they have not been initiated within 90 days before baseline, provided these data are available); if there is no info available this criterion will be dropped.
   - Carbamazepine, primidone, valproate
   - Drugs for which there is a warning or precaution in the labeling about methemoglobinemia at approved doses (e.g., dapsone, local anesthetics such as benzocaine used chronically, primaquine and related antimalarials)

However, if applying this criterion results in small cohort, it will be removed, and this will be acknowledged as a study limitation**.**

Dose/Route/Regimen

For the duration of the study Trx-237-039, all participants received tablets orally at a dose of 16 mg/day.

Patients selected from CPAD database will be considered as the control group; CPAD only contains placebo data from clinical trials.

Methodology

This study includes only data from the HMTM 16mg/day arm of study TRx-237-039. This was a two-phase outpatient study of HMTM administered as monotherapy in approximately 500 subjects (450 under protocol version 5.0 or higher) with early to mild/moderate AD. It comprised a randomized, double-blind, controlled, 52-week treatment phase followed by a 52-week open-label treatment phase that represents a modified delayed start of treatment. Subjects for whom legally acceptable informed consent was obtained and who were found eligible on the basis of screening evaluations, were randomly assigned at baseline to receive either HMTM 16 mg/day, HMTM 8 mg/day, or control (i.e., MTC) (4:1:4, at the study level); the drug supplies for the control group included tablets containing a urinary colorant (MTC), 4 mg, dosed at an average frequency of one tablet twice weekly.

In the current analysis, outcome data collected during the 52-week double-blind treatment phase, as well as during the 104-week period (double-blind + open-label) for HMTM 16mg/day will be used. Week 78 is considered the primary endpoint as it is assumed to provide the optimal balance between data availability in CPAD and from TRx-237-039.

Assessments

Name of Sponsor / Company: TauRx Therapeutics Ltd (TauRx)
Name of Finished Product: HMTM (TRx0237) Film-coated Tablets, 4 mg
Name of Active Ingredient (Drug Substance): Hydromethylthionine Mesylate
**Efficacy:**<br>The clinical efficacy scale ADAS-Cog_{13} will serve as the primary efficacy scale, unless there is insufficient data, in which case the analysis will be changed as per SAP specification. The co-primary endpoint is WBV (unless there are insufficient data, see SAP).
**Statistical Analyses**<br>Participants from the CPAD cohort will be matched to TRx-237-039 participants who were receiving HMTM 16mg/day based on propensity score to make their baseline demographics and clinical characteristic comparable. These include age, sex, education, diagnosis (MCI/AD), smoking history, ApoE Genotype, MMSE and CDR, and amyloid positivity at baseline; final selection will depend on the availability of parameters in the CPAD database. The variables are selected as covariates in the propensity score matching. If any of these covariates leads to an overall reduction in sample size of 50% or more, covariates with highest reduction in sample size will be removed from the analysis.<br><br>All participant characteristics and outcome variables are summarized descriptively for unmatched and matched cohorts to show adequacy of matching and highlight any differences identified. Tabulations are produced for appropriate baseline characteristics and outcome variables. For categorical variables, summary tabulations of the number and percentage within each category (with a category for missing data) are presented. For continuous variables, mean with standard deviation (SD), median interquartile ranges (IQR), minimum and maximum values are presented. The difference between cohorts are assessed by t-test for continuous variables and a comparison of proportions or chi-squared test for categorical variables. No adjustment for multiplicity is planned in this analysis.<br><br>All primary and secondary outcomes are analyzed using a linear model, including changes from baseline at 78 weeks as the dependent variable, and treatment group as the independent variable. Since the propensity score matching results are in paired samples, an additional mixed linear model with random intercept on propensity score-matched pairs will also be used. For secondary endpoints data from week 52 will be used.

5 ABBREVIATIONS

Abbreviations	Definitions
AChEI	acetylcholinesterase inhibitor
AD	Alzheimer’s Disease
ADAS-cog$_{11}$	Alzheimer's Disease Assessment Scale – Cognitive Subscale (11-item)
ADAS-cog$_{12}$	Alzheimer's Disease Assessment Scale – Cognitive Subscale (12-item)
ADAS-cog$_{13}$	Alzheimer's Disease Assessment Scale – Cognitive Subscale (13-item)
ADCS-ADL$_{23}$	Alzheimer's Disease Cooperative Study – Activities of Daily Living (23-item)
ADCS-CGIC	Alzheimer’s Disease Cooperative Study-Clinical Global Impression of Change
ADL	Activities of daily living
ADR	Adverse Drug Reaction
AE	adverse event
ApoE	Apolipoprotein E gene
ARIA	Amyloid-related Imaging Abnormalities
bvFTD	behavioral variant frontotemporal dementia
CDR	Clinical Dementia Rating
CJD	Creutzfeldt-Jakob Disease
CNS	central nervous system
CPAD	Critical Path for Alzheimer’s Disease
CSF	Cerebrospinal fluid
ECG	Electrocardiogram
EEG	Electroencephalogram
E-MITT	Efficacy Modified Intent-to-Treat
EU	European Union
FDA	Food and Drug Administration (United States)
^{18}F-FDG-PET	^{18}F-fluorodeoxyglucose positron emission tomography
g, kg, mg, ng, pg	gram, kilogram, milligram, nanogram, picogram
G6PD	glucose-6-phosphate dehydrogenase
GCP	Good Clinical Practice
HMT	hydromethylthionine mesylate
HMTM	hydromethylthionine mesylate, leuco-methylthioninium bis(hydromethanesulfonate)
ICH	International Council for Harmonisation of Technical Requirements for Pharmaceuticals for Human Use
IQR	Interquartile range
ITT	Intent-To-Treat
L, mL, dL	liter, milliliter, deciliter
LMTB	leuco-methylthioninium bis(hydrobromide)
LSM	Least Squares Mean
LVV	lateral ventricular volume
MAO	monoamine oxidase
MCI-AD	mild cognitive impairment due to AD
MD	Doctor of Medicine
MedDRA	Medical Dictionary for Regulatory Activities
MI-MITT	MRI Imaging Intent-to-Treat

Abbreviations	Definitions
MMSE	Mini-Mental State Examination
MRI	magnetic resonance imaging
MT	Methylthioninium
MTC	methylthioninium chloride
NIA	National Institute on Aging
NfL	Neurofilament light chain
NMDA	N-methyl-D-aspartate
nvCJD	new variant Creutzfeldt-Jakob Disease
PET	Positron Emission Tomography
PHF	paired helical filament
PK	Pharmacokinetic
Ptau	Phospho-tau
SAP	Statistical Analysis Plan
SD	Standard deviation
SE	Standard error
SOP	Standard Operating Procedure
SPM	statistical parametric mapping
tid	three times daily
TSE	Transmissible Spongiform Encephalopathy
UK	United Kingdom
ULN	Upper limit of normal
USAN	United States Abbreviated Name
vCJD	variant Creutzfeldt-Jakob Disease
WBV	whole brain volume

6 BACKGROUND AND RATIONALE FOR THE STUDY

TauRx Therapeutics Management Ltd. (hereby referred to as TauRx) is developing Hydromethylthionine mesylate (HMTM) for treatment of tauopathies and other protein misfolding disorders. Tauopathies are a class of neurodegenerative diseases associated with pathological aggregation of tau protein in the human brain. The tauopathies include diseases that primarily affect cognition such as Alzheimer’s disease (AD). AD is an irreversible neurodegenerative disease that results in progressive loss of cognitive function and ability to manage daily living, and ultimately complete loss of mental faculties. It represents the most common type of dementia accounting for an estimated 60% to 80% of cases (Alzheimer’s Association, 2022). Fifty-five million people have been estimated to be affected by dementia worldwide, including 10 million in Europe (Prince et al., 2015) and an estimated 6.5 million in the United States alone (Alzheimer’s Association, 2023).

In AD, the microtubule associated protein tau is redistributed exponentially into paired helical filaments (PHF) forming neurofibrillary tangles that correlate with pyramidal cell destruction (Wischik et al., 1997). There is a robust clinico-pathological correlation between tau pathology, tau aggregation, and clinical measures of dementia (Bierer et al., 1995; Mukaetova-Ladinska et al., 2000). These relationships are maintained from the earliest detectable stages of dementia and progress in parallel with clinical deterioration and are also seen by imaging using recently developed tau ligands (Lockhart et al., 2016; Pontecorvo et al., 2017; Xia et al., 2017).

The repeat-domain tau fragment originally identified biochemically as a structural constituent of the tangle PHF core (Wischik et al., 1988a, 1988b) has recently been confirmed using cryo-electron microscopy (Fitzpatrick et al., 2017). This fragment assembles spontaneously *in vitro* to form PHF-like filaments (Al-Hilaly et al., 2017). The active moiety required to block filament assembly is the reduced HMT form of MT (Al-Hilaly et al., 2018).

As of 2024, drugs currently available to treat AD, such as acetylcholinesterase inhibitors (AChEIs) or memantine, are symptomatic treatments that address certain central neuronal dysfunctions associated with AD, but are not known to directly affect the neurofibrillary tangles in the brain that represent a core pathological component of AD. Controlled studies with the AChEIs donepezil, rivastigmine, and galantamine have demonstrated small improvements in cognitive tests and global measures of change in selected subjects with mild to moderate AD over 3 to 12 months (Birks and Harvey, 2003; Olin and Schneider, 2002). However, improvements in function and behavior have been demonstrated less reliably with AChEIs. Furthermore, although these medications provide benefits for some subjects, their effectiveness is often limited in duration and they do not affect the rate of progression of the disease (Courtney et al., 2004).

Memantine is a noncompetitive, low-affinity, N-methyl-D-aspartate (NMDA) receptor antagonist that might prevent calcium-mediated glutamate excitatory toxicity in AD. Studies with memantine over 6 months in subjects with moderate to severe AD have shown small benefits on cognition, global measures, daily living activities, and behavior (Reisberg et al., 2003), but evidence for a benefit is lacking in mild AD (Schneider, 2011).

Aduhelm™ (aducanumab), an amyloid beta-directed antibody, was granted accelerated approval by FDA in June 2021 for early AD based on a biomarker (reduction of amyloid plaque). However, manufacturing was discontinued in 2024. In the U.S.A Leqembi (lecanemab) received similar approval in July 2023 and Kisunla (donanemab) was approved July 2024. One particularly significant limitation in the widespread use of such drugs is the requirement for monthly (Kisunla) or bi-weekly (Leqembi) intravenous infusions of the monoclonal antibody preparations that target aggregated forms of amyloid. This has a substantial impact on the costs of production, distribution, and administration of such treatments. Based on the clinical trials to date, there are uncertainties regarding the effectiveness of monoclonal antibody therapies on clinical outcomes. In addition, there is a significant risk of triggering amyloid-related imaging abnormalities (ARIAs), requiring regular monitoring by magnetic resonance imaging (MRI) scans (summarized in the Aduhelm FDA Drug Approval Package, June 2021 and the Leqembi FDA Drug Approval Package, July 2023). These factors may have contributed to the withdrawal from the US market of aducanemab in January 2024.

Therefore, an unmet need exists to develop new medications for AD that modify the underlying disease pathology more directly and offer longer-term and greater efficacy. HMTM, the investigational product, is believed to have the potential to confer benefits over existing treatments for AD due to its ability to affect the process of tau aggregation responsible for the underlying neurofibrillary pathology of AD. Available nonclinical and clinical evidence supports the clinical evaluation of HMTM in AD.

The objective of this particular comparison is to allow a direct comparison of HMTM 16 mg/day with a control group of actual placebo subjects that are closely matched thereby overcoming weaknesses of meta-analytical comparisons and using actual data from a clinical trial population thereby overcoming the weakness of using natural history data.

6.1 Background

6.1.1 Investigational Product

The investigational product is HMTM (USAN hydromethylthionine mesylate, also referred to as leuco-methylthioninium bis(hydromethanesulfonate), hydromethylthionine bis(hydromesylate), leucomethylene blue dimesylate, and its code name, TRx0237). As a dihydromethanesulfonate salt (also known as mesylate), HMTM stabilizes the reduced crystalline form of the MT moiety in the solid state.

HMTM is provided as 4-mg, immediate-release tablets, to be taken twice daily; the pharmacokinetics (PK) of HMTM 4 mg as a single dose and given twice daily at steady state (8 mg/day) have been characterised. Following dissociation of the counter ions, the uncharged (reduced) HMT form, *i.e.*, the active moiety, is absorbed passively. The charged (MT^+) form does not have the conformation necessary to block filament assembly (Al-Hilaly et al., 2018). When dosed as the charged (MT^+) form (as in methylthioninium chloride [MTC], also known as methylene blue), MT^+ requires an additional reduction step (to HMT) to be distributed to the brain (Baddeley et al., 2015). Within cells, MT exists in equilibrium between the reduced (HMT) and oxidized (MT^+) forms, the predominant form present depending on the cellular milieu, *i.e.*, pH and reductive capacity within that cell, with the HMT form predominating intracellularly (May et al., 2004).

6.1.2 Clinical Data

6.1.2.1 Efficacy

In a double-blind, placebo-controlled Phase 2 study (TRx-014-001) of male and female subjects with mild or moderate AD (AChEIs and memantine excluded), MTC was administered orally at doses of 30 mg three times daily (tid), 60 mg tid, and 100 mg tid (total doses of 69 mg/day, 138 mg/day, and 228 mg/day MT base equivalents, respectively). MT 138 mg MT/day slowed the clinical rate of decline on the Alzheimer's Disease Assessment Scale – cognitive subscale (ADAS-cog) and the Mini-Mental State Examination (MMSE) measured over 1 year. These effects and the benefit on the ADAS-cog, Alzheimer's Disease Cooperative Study-Clinical Global Impression of Change (ADCS-CGIC), and MMSE scales were already evident in subjects with moderate disease at Baseline within the first 6 months in an analysis of the entire population with severity as an interaction term. The 6-month analysis and methodology were pre-specified as part of the primary analysis. The result in subjects with moderate disease severity at 6 months remained significant after correction for multiple comparisons. Longer term efficacy was confirmed in mixed mild/moderate subjects by *post hoc* analyses at 50 weeks and 102 weeks, and efficacy was also confirmed in analyses of a variety of secondary endpoints (Wischik et al., 2015). The lower dose (69 mg MT/day) was below the minimally effective dose. The capsule formulation used limited the absorption of MT at the 228 mg MT/day dose due to a combination of dose-dependent delay in dissolution of the 100-mg MTC capsules used in the study and a formulation-independent limitation in the ability to absorb MT at the highest dose in the presence of food when administered as MTC. When both of these factors were taken into account, the clinically effective dose available for release within 60 minutes in subjects receiving a nominal dose of 228 mg MT/day was equivalent to approximately 69 mg MT/day (Baddeley et al., 2015), and the total available dose was equivalent to 109 mg MT/day (Wischik et al., 2015).

Evidence of clinical efficacy was supported by neuroimaging in 138 mild and moderate AD subjects imaged at Baseline and at 24 weeks. Despite lack of decline over this time detected by neuropsychological measures such as ADAS-cog in subjects with mild disease at Baseline, statistically significant decline in neuronal function as measured by regional cerebral blood flow was detected using hexamethylpropylamine oxime-single photon emission computed tomography in all predefined lobes in the region-of-interest analysis and was particularly marked in the inferior medial temporal and temporo-parietal regions of the neocortex on the statistical parametric mapping (SPM) analysis, areas characteristically affected by the tau aggregation pathology of AD and the regions primarily affected in the Braak staging system (Braak and Braak, 1991). MTC at a dose of 138 mg MT/day was found to eliminate this decline entirely (Wischik et al., 2015). A similar effect was seen in a smaller group of 20 subjects imaged by ^{18}F-fluorodeoxyglucose positron emission tomography (^{18}F-FDG-PET) to measure neuronal function by glucose uptake. The effect on functional neuroimaging markers at 24 weeks in subjects with mild AD was found to be predictive of a clinical response at 50 weeks (Wischik et al., 2015). The reduced efficacy of the 100-mg capsule dose (228 mg MT/day) relative to the 60-mg capsule dose (138 mg MT/day) was also confirmed by neuroimaging.

Two Phase 3 studies of HMTM in AD subjects have been completed (Gauthier et al., 2016; Wilcock et al., 2018) and reported. These were a 15-month study in subjects with mild to moderate AD comparing doses of 150 mg/day and 250 mg/day with a control arm of

8 mg/day (TRx-237-015), and an 18-month study in subjects with mild AD comparing a dose of 200 mg/day and the 8-mg/day control (TRx-237-005). AChEIs and/or memantine were used concomitantly by approximately 80% to 85% of the subjects depending on study. Briefly, there was no statistically significant difference between HMTM in the higher doses of 150 to 250 mg/day in either of the two co-primary clinical efficacy endpoints or lateral ventricular volume (LVV) when compared with the dose of HMTM 8 mg/day, intended as a control. Based on the results of the Phase 2 study, it was assumed that a dose of 8 mg/day of HMTM would be without activity and could thus be used to control for urinary coloration because of concerns about blinding. Further analyses of results from both studies demonstrated statistically significant exposure-dependent effects of HMTM 8 mg/day given as monotherapy or as add-on to standard symptomatic treatments for AD on the 11-item version of the ADAS-cog (ADAS-cog$_{11}$) and also on imaging measures of progression of brain atrophy. There was no greater benefit at the high doses (150 and 250 mg/day approximately ≥20-fold higher doses) relative to the 65% with therapeutic levels of exposure at the 8 mg/day dose. The treatment differences relative to the low-exposure proxy placebo group were reduced by half at all doses when HMTM was given as add-on to standard symptomatic treatments for AD. In Study TRx-237-005, the differences between monotherapy and add-on therapy were confirmed as modified primary outcomes. The reduction in treatment effects of HMTM by treatment with an AChEI or memantine has been reproduced in both wild-type and tau transgenic mouse models and appears to reflect a generalized homeostatic downregulation that is induced in multiple brain systems to compensate for the chronic activating effects of symptomatic treatments (Riedel et al., 2020).

6.1.2.1.1 TRx-237-039

A monotherapy phase 3 study (TRx-237-039), which concluded in April 2023 and has been reported recently, was conducted in subjects with mild to moderate AD and MCI-AD and included 12-Month double-blind period followed by a 12-Month Open-Label Treatment. This study compared doses of 8 mg/day and 16 mg/day with a control group (containing MTC 4 mg dosed twice per week on a varying schedule as a urinary colorant to preserve study blind). The lowest dose needed to maintain blinding was found to have clinically relevant symptomatic activity over the initial 12 months. This resulted from a combination of the atypical PK of the HMT moiety and the dual mechanisms of action seen in preclinical studies, namely inhibition of pathological aggregation of tau and symptomatic activity at clinically relevant levels of exposure. It is thought that the symptomatic activity results from increased levels of acetylcholine in the hippocampus demonstrated in preclinical studies. This confounded the ability to detect a difference between subjects receiving HMTM 16 mg/day and the intended control as both had clinically active exposure to HMT. Despite these challenges it has been possible to provide five sources of evidence in this study to support the efficacy of HMTM at 16 mg/day. The first is the demonstration of a dose-dependent treatment effect on change in NfL, an endpoint that reflects disease progression. The comparison of HMTM 16 mg/day relative to control was as-randomized, and was not confounded by symptomatic activity in the control arm. The second is a directionally supportive difference in the MCI-AD subpopulation with CDR 0.5 of a significant difference in the conversion rate from CDR 0.5 to CDR ≥1 over 12 months between subjects randomized to HMTM 16 mg/day and MTC 8 mg/week. The third is the statistically significant observed-case difference in the MCI-AD subpopulation between subjects originally randomized to HMTM 16 mg/day or the MTC 8 mg/week control over 18 and 24 months. The fourth is the statistically significant observed-case improvements in cognitive function over Baseline that were measurable from 6 months through to 18 months in subjects

with MCI-AD. The fifth is the demonstration that subjects treated with HMTM do not follow the typical natural history of AD in terms of global disease progression, clinical decline, or loss of WBV using external sources of data. The totality of evidence available as described above is consistent and supports the conclusion that HMTM 16 mg/day is effective in reducing clinical decline and loss of WBV relative to the expected disease trajectory in AD populations.

6.1.2.2 Safety

Overall, 2856 subjects have received at least one dose of HMTM to date in one of 18 completed clinical studies. Of these, 2206 subjects were diagnosed with MCI-AD or mild to moderate AD and 211 with behavioral variant frontotemporal dementia (bvFTD), the majority receiving doses of 150 mg/day or higher. A total of 1997 subjects were treated with HMTM for at least 6 months or longer, and 1616 were treated with HMTM for at least 1 year. In addition to studies of HMTM, the safety database also includes six studies in which an additional 505 subjects were exposed to closely related salts in TauRx-sponsored studies. The numbers of subjects exposed and the duration of treatment greatly exceeds expectations as outlined in the International Council for Harmonisation of Technical Requirements for Pharmaceuticals for Human Use (ICH) E1A The Extent of Population Exposure to Assess Clinical Safety: For Drugs Intended for Long-term Treatment of Non-Life-Threatening Conditions (March 1995).

Standard safety assessments were implemented in the completed studies. These are widely used and generally recognized as reliable, accurate, and relevant and include adverse event (AE) recording/review, clinical laboratory tests of blood, vital sign and body weight measurements, and physical examinations. These were to be undertaken at each clinic visit. Selected safety assessments were completed in a subset of the studies based on the evolving safety profile of HMTM. Electrocardiograms (ECGs) and laboratory tests of urine were undertaken in earlier adequate and well-controlled Phase 2 and 3 studies of HMTM. Additional targeted monitoring was also performed consistent with the known pharmacology of HMTM, including pulse co-oximetry to assess for methemoglobinemia and neurologic examinations for potential serotonin toxicity; questioning for suicidality, because the drug has central nervous system activity; and amyloid-related imaging abnormalities [ARIA] because of the indication under study. Given the lack of adverse findings in these targeted monitoring assessments, they were no longer undertaken routinely in the most recent Phase 3 study. Ophthalmological examinations were added as a targeted monitoring assessment following an observation of the potential for coloration of implanted lenses.

The primary toxicity of HMTM is hematological, manifesting as dose-dependent anemia, not clinically significant, and potentially a regenerative hemolytic anemia. Six percent (6%) of subjects randomized to HMTM 16 mg/day had descriptive terms consistent with anemia (i.e., all MedDRA terms reflective of anemia such as hemoglobin decreased, etc.). Changes in mean hemoglobin values over time are small (approximately 2 g/L on average) and similar to control. If reductive capacity in red cells needed to maintain HMT in the reduced form is exceeded, hemolytic anemia may ensue as a result of conversion of HMT to the MT^+ form which can oxidise the heme iron of haemglobin. There has only been one case at a relevant dose (8 mg/day); the remaining cases have been at higher doses (≥200 mg/day). The risk of progressing to hemolytic anemia is managed by contraindicating use in subjects with known G6PD deficiency and preexisting hemolytic anemia as well as warning about use in subjects

who might be at risk either by medical condition or concomitant drug use. Routine monitoring is advised in such cases.

The other most common ADRs potentially result in intolerability, but do not commonly require interruption or discontinuation of treatment at the 16-mg/day dose. These are diarrhea (2%) and headache (1%). Only rarely have these been of clinical consequence or required an action with study drug, and do not require special expertise or procedures for monitoring.

With respect to non-hematological laboratory abnormalities, the overall assessment is that HMTM may be associated with mild hepatocellular injury, without compelling evidence of impaired hepatic function. Dose-related, isolated increases in transaminases without accompanying effects on bilirubin are observed, with approximately 1% of subjects experiencing values ≥3-fold ULN in one or both. HMTM does not appear to have a clear association with other clinical laboratory abnormalities.

The less common risks of HMTM (<0.1%, regardless of dose) are largely derived from its pharmacological properties and the relevance to the recommended daily dose of 16 mg/day is not clear. Most significant of these is the potential for serotonin syndrome (a potentially life-threatening drug reaction) given that the oxidized MT^+ moiety inhibits MAO in vitro (albeit at concentrations much higher than achievable clinically) and cases have been reported in the published literature for MTC, mostly following intravenous administration. There have been 5 possible cases on HMTM, none of which had a presentation or clinical course typical of serotonin syndrome. As a precaution, subjects using potent serotonergic drugs should be informed of the potential risk. Importantly, whilst caution is advised, there has been no report of hypertensive crisis with HMTM or in the published literature for MTC, thus coadministration with tyramine-rich foods or drinks is not likely a risk. Hypersensitivity is a potential risk, especially as anaphylactic reactions have been reported for MTC, confirmed by skin testing, and related dyes with evidence for cross-reactivity. One subject treated with HMTM necessitated treatment with epinephrine for a severe allergic reaction shortly after reinitiating treatment. Finally, given its light absorbing and photoreactive properties and distribution into the skin and eyes, photosensitivity is possible. No special precautions were advised in the clinical studies and there has only been one case reported. These less common risks will be monitored in routine safety-monitoring.

Additional details regarding clinical findings from studies are described in the Investigator's Brochure.

6.2 Rationale

HMTM 16 mg/day has shown evidence of efficacy in a pivotal, randomised, double-blind and well-controlled Phase 3 study in subjects with MCI-AD and mild to moderate AD (TRx-237-039). HMTM treatment effects are seen on multiple clinical and biological endpoints in three distinct trials in AD. The HMTM 16-mg/day dose, chosen to result in therapeutic concentrations in the large majority of subjects, HMTM has a clinically meaningful benefit to patients with MCI-AD and mild to moderate AD that is qualitatively distinct from currently available therapies. It is well tolerated and has a robust safety profile.

A major challenge encountered throughout the clinical development of HMTM has been the problem of maintaining blinding in clinical studies. A true placebo cannot be used, as HMTM causes slight blue-green urinary coloration, which has the potential to unblind the patient,

caregiver and physician. TauRx conducted extensive searches and analyses to identify an inactive, safe product that could be formulated in a placebo in order to produce similar urinary coloration in the study control arm. The only substances identified were variants of methylthionine (MTC or HMTM). When used at the lowest unit dose (4 mg) shown to produce equivalent perception of urinary coloration in volunteers, MTC given as 4 mg twice weekly was found to retain statistically significant symptomatic activity. It has therefore not been possible to manufacture a true inactive placebo that maintains appropriate blinding in clinical studies. The limitation was demonstrated in the earlier Phase 3 studies TRx-237-015 and TRx-237-005. In these trials doses in the range 150 – 250 mg/day were compared with 8 mg/day intended as an inactive urinary colorant on the basis of results of an earlier Phase 2 dose-finding study which found that a dose of 138 mg/day was found to be required to produce clinical and neuroimaging benefits.

In the Phase 2 dose-finding study, MTC was used to deliver hydromethylthionine, but was found subsequently to have impaired bioavailability, a limitation that HMTM was designed to overcome (Baddeley et al., 2015). MTC 4 mg was given every 2 to 4 days (twice weekly on average) in study TRx-237-039. The results of the study showed symptomatic therapeutic activity even at this low dose of MTC, partially confounding the interpretation of the true effect of HMTM as a disease-modifying treatment for AD within the double-blind phase of the study. The explanation for symptomatic activity at such a low dose is that hydromethylthionine, whether provided as MTC or HMTM, has an unusual pharmacokinetic clearance profile such that plasma levels increase over 12 months before returning to the 1-month level at 24 months. This increase over 12 months resulted in plasma levels reaching levels equivalent to MTC doses which had been shown to reverse learning deficits produced by scopolamine in standard mouse model of symptomatic activity (Deiana et al., 2009).

Therefore, in view of the challenge discussed, and in order to establish supplementary comparative data using an additional source of controls, it essential to use closely matched historical controls from placebo arms in clinical trials available in a US Alzheimer’s Disease database Critical Path for Alzheimer’s Disease (CPAD) in prespecified analyses.

7 OBJECTIVES

The primary objectives of the study pertain to comparative analysis of TRx-237-039 with a historical clinical trial control cohort obtained from the CPAD database (Sivakumaran et al., 2020). They will focus on mild cognitive impairment due to Alzheimer's Disease (MCI-AD) and probable AD (mild/moderate AD) compared with a matched control group of the CPAD dataset. The analysis is pre-defined in the sense that CPAD data has not been accessed or seen by the statistics provider Cytel or the Sponsor.

7.1 Primary Objectives

To compare the HMTM dose of 16 mg/day from completed study TRx-237-039 with a matched historic control group from the CPAD dataset on the following endpoints at 78-weeks:

a. Alzheimer's Disease Assessment Scale, 13-item version (ADAS-cog$_{13}$) in all participants at a two-sided alpha of 0.05;
b. Whole brain atrophy in all participants at a two-sided alpha of 0.05.

Both co-primary endpoints need to reach statistical significance. If there are insufficient ADAS-Cog$_{13}$ data available in the CPAD dataset, ADAS-Cog$_{12}$ or ADAS-Cog$_{11}$ will be used dependent on availability. ADAS-Cog$_{11}$ will in any case provide a sensitivity analysis. If there are insufficient WBV data from CPAD, other brain volumes captured in the volumetric MRI will be used with temporo-parietal being the key if available.

8 STUDY DESIGN

8.1 General Description

This is an analysis of HMTM 16 mg/day arm data from TRx-237-039 using a propensity score matched CPAD cohort as an external control. The analysis is pre-specified in the sense that neither the statistics provider Cytel nor the study Sponsor had access to the CPAD data.

TRx-237-039 was a two-phase outpatient study of HMTM administered as monotherapy in approximately 500 subjects (450 under protocol version 5.0 or higher) with early to mild-moderate AD: a randomized, double-blind, controlled, 52-week treatment phase followed by a 52-week open-label treatment phase that represents a modified delayed start of treatment. Subjects for whom legally acceptable informed consent was obtained and who were found eligible on the basis of screening evaluations, were randomly assigned at baseline to receive either HMTM 16 mg/day, HMTM 8 mg/day, or control (i.e., placebo/MTC) (4:1:4, at the study level); the drug supplies for the control group included tablets containing a urinary colorant (MTC), 4 mg, dosed at an average frequency of two tablets per week given at varying intervals between 2 to 4 days.

The primary treatment group comparison during the double-blind treatment phase was between HMTM 16 mg/day and control. Following completion of the 52-week treatment phase, all subjects (regardless of randomized treatment assignment or response) continued open-label treatment with HMTM 16 mg/day for a further 52 weeks.

In the current analysis, outcome data collected in subjects receiving HMTM 16 mg/day during the 52-week double-blind treatment phase, as well as during the 104-week period (double-blind + open-label) will be used.

TRx-237-039 data was collected and coded following the Clinical Data Interchange Standards Consortium (CDISC) standards (version 1.4; implementation guides v3.2).

The CPAD database includes patient-level data from 12,811 patients across 36 clinical trials of AD and MCI. The database contains, but is not limited to, demographic information, ApoE4 genotype, concomitant medications, and cognitive scales (MMSE and ADAS-Cog). Limited treatment-arm data and limited AD biomarker data (biofluids, tau or amyloid positron emission tomography (PET), EEG data) are available. Patients selected from CPAD database who had been in placebo arms of trials will be considered as control.

8.2 Study Population

Participants are to have a clinical diagnosis of probable AD or MCI-AD. A documented positive amyloid PET is required for the MCI-AD subgroup only. The allowable severity will be a MMSE score of 16 to 27 (inclusive) and Global CDR score of 0.5 to 2 (if 0.5, including a score of >0 in one of the functional domains). Inclusion and exclusion criteria are given in Section 9.

9 PARTICIPANT ENROLLMENT

Participants from TRx-237-039:

The inclusion and exclusion criteria for TRx-237-039 are available in the respective study protocol. The planned TRx-237-080 will use similar inclusion and exclusion. However, if applying a particular criterion results in a cohort that is too small for analysis, it will be removed, and this will be acknowledged as a study limitation.

9.1 Inclusion Criteria

To be eligible for enrollment in this study, a participant must meet all of the following inclusion criteria, including CPAD data:

1) Probable AD or MCI due to AD (MCI-AD; MCI-AD is referred to as MCI in the sections below)
2) PET scan positive for amyloid in the MCI-AD subgroup; a PET scan positive for amyloid is not needed in the mild/moderate AD subgroup as disease is progressed sufficiently for PET confirmation to be no longer needed. Amyloid PET scans have been used generally in trials in mild/moderate AD. If data availability for PET scan in the CPAD cohort is limited, the main analysis will be conducted without this criterion, but a sensitivity analysis including this criterion will be performed.
3) MMSE score of 16 – 27 at screening
4) Global CDR score of 0.5 to 2 at screening (if 0.5, including a score of > 0 in one of the functional domains: Community Affairs, Home and Hobbies, or Personal Care). If data availability for CDR in CPAD cohort is limited, this criterion will be dropped
5) Outpatients below 90 years at screening (patients residing in hospitals or moderate to high dependency continuous care facilities are excluded)

9.2 Exclusion Criteria

The exclusion criteria are:

7. Significant central nervous system (CNS) disorder other than probable AD or MCI-AD, *e.g.*, Lewy body dementia, Parkinson’s disease, multiple sclerosis, progressive supranuclear palsy, hydrocephalus, Huntington’s disease, any condition directly or indirectly caused by Transmissible Spongiform, Encephalopathy (TSE), Creutzfeldt-Jakob Disease (CJD), variant Creutzfeldt-Jakob Disease (vCJD), or new variant Creutzfeldt-Jakob Disease (nvCJD)

8. Significant neuroimaging findings e.g. significant intracranial focal or vascular pathology seen on brain MRI including but not limited to:
   - Large confluent white matter hyperintense lesions (*i.e.*, Fazekas score of 3)
   - Other focal brain lesions judged clinically relevant by the investigator
   - Evidence of a prior or current macrohemorrhage
9. Mental Disorders such as
   - Current major depressive disorder (MDD)

- History of schizophrenia
- Other psychotic disease, bipolar disorder within past 5 years from the baseline

  Substance (including alcohol) related disorders within past 5 years from the baseline

10. History of any conditions that may be impacting cognitive function:
    - Cerebrovascular accident
    - Transient ischemic attack
    - Significant head injury, for example, associated loss of consciousness, skull fracture or persisting cognitive impairment
    - Other unexplained or recurrent loss of consciousness
11. Diagnosed epilepsy (a single prior seizure >6 months prior to Screening is considered acceptable)
12. Treatment currently or within 30 days before the baseline with:
    - Acetylcholinesterase inhibitor (AChEI) and/or memantine
    - Clozapine (other antipsychotics are allowed if they have not been initiated within 90 days before baseline provided these data are available); if there is no info available this criterion will be dropped.
    - Carbamazepine, primidone, valproate
    - Drugs for which there is a warning or precaution in the labeling about methemoglobinemia at approved doses (e.g., dapsone, local anesthetics such as benzocaine used chronically, primaquine and related antimalarials).

10 STUDY ASSESSMENTS

10.1 Assessment of Clinical Efficacy

10.1.1.1 ADAS-cog

The ADAS was designed to evaluate the severity of cognitive and non-cognitive or behavioral symptoms of AD (Rosen et al., 1984). The ADAS-cog is the cognitive subscale of the ADAS, originally proposed with 11 items. The ADAS-cog_{11} consists of tasks that measure memory, orientation, language, and praxis resulting in scores that range from 0 to 70, with higher numbers indicating greater impairment. Two additional items have been added in the ADAS-cog_{13} to provide additional sensitivity to change in cognition at earlier stages of the disease (Mohs et al., 1997); these are Delayed Word Recall and Number Cancellation.

10.2 Imaging Assessments and Procedures

10.2.1 Imaging Methods for Efficacy

10.2.1.1 MRI

Imaging assessments were to be made by central readers as follows:

- Brain MRI will be evaluated for change in whole brain volume.

If there is insufficient WBV data from CPAD, other brain volumes captured in the volumetric MRI will be used with temporo-parietal being the key if available.

11 STATISTICAL ANALYSIS

The SAP is attached to this protocol and will provide details about the planned analysis. A brief overview of the plans for the primary analysis is given in the following sections.

The primary target of estimation is the difference in ADAS-cog_{13} over 78 weeks in participants randomized to the HMTM dose of 16 mg/day as compared to a matched historic placebo control group of the CPAD dataset.

11.1 Efficacy Endpoints

11.1.1 Primary And Secondary Efficacy Endpoints

To compare the HMTM dose of 16 mg/day from completed study TRx-237-039 with a matched historical placebo control group from the CPAD dataset on the following endpoints at 78 weeks:

- Alzheimer's Disease Assessment Scale, 13-item version (ADAS-cog_{13}) in all participants at a two-sided alpha of 0.05;
- Whole brain atrophy in all participants at a two-sided alpha of 0.05.

Corresponding secondary endpoints of CDR sum of boxes and MMSE as well as exploratory endpoints and sensitivity analysis are described in the SAP in section 3.2.

11.2 Number of Participants and Sample Size Calculation

The primary comparison compares change in ADAS-Cog_{13} with historical matched placebo controls from the CPAD dataset in MCI-AD&AD (all participants).

The sample size is dictated by the availability of data from TRx-237-039 and CPAD. The SAP attached to this protocol states the criteria which are applied should the resulting data set be too small for the desired comparisons.

11.3 Analysis Populations

The following participant populations will be used for the statistical analyses;

- Intent-to-Treat (ITT) population will include all enrolled subjects. Two subsets are defined as follows:
  - The Efficacy Modified Intent-to-Treat (E-MITT) population will include all randomized participants who have taken at least one dose of the study drug at 16 mg/day and have a baseline and at least one valid post-baseline efficacy assessment in the treatment period (prior to any potential follow-up assessment).
  - An MRI Imaging Modified Intent-to-Treat (MI-MITT) population will include all randomized participants who took at least one dose of study drug at a 16 mg/day and have at least one valid baseline and at least one valid post-baseline volumetric MRI.
- For the CPAD cohort, to maximize the resulting sample size, the matching process of the primary analysis will include all patients who meet the selection criteria described in the section 9.

11.4 Clinical Efficacy and Imaging Analysis

The various analyses planned in the study are outlined briefly below. The SAP provided in section 3.2 provides further detail on the planned, pre-defined analyses, and models to be utilized.

For E-ITT and MI-MITT analyses, only valid data will be included in the primary and secondary analyses. In participants who have initiated treatment with an AChEI and/or memantine, assessments made after initiation of such treatment are not considered valid as they could confound the interpretation of the results and will be excluded. Sensitivity analyses will be provided for the primary analyses including all data.

ITT and E-MITT population weighted least squares means (LSM), unless otherwise stated, and 95% confidence intervals will also be included; the population will be determined by the corresponding model, dependent on whether the endpoint is an imaging endpoint or a clinical efficacy endpoint. Where requested, annualized rates will be estimated based on the population-weighted LSM.

Data listings will include all observed data for the primary endpoints.

11.4.1 Hypothesis

Two efficacy outcomes, ADAS-cog$_{13}$ and WBV will be analyzed as primary endpoints in all participants at 78-weeks. The primary endpoint must reach significance based on the use of a two-sided test at the alpha = 0.05 level of significance for HMTM to be designated as superior to a matched historic control group of the CPAD dataset.

The global null hypotheses are as follows:

> H_{01}: There is no difference between the matched HMTM 16 mg/day MCI-AD and mild/moderate AD subpopulation and matched historic control group of the CPAD dataset in ADAS-cog$_{13}$ from baseline to Week 78.
>
> H_{02}: There is no difference between the matched HMTM 16 mg/day MCI-AD and mild/moderate AD subpopulation and matched historic control group of the CPAD dataset in WBV from baseline to Week 78.

11.4.2 Propensity score matching

Refer to the SAP (Attached to this document; Section 4.4) for details about the planned analysis. Briefly, to control for confounders in the comparison of treatments used in TRx-237-039 versus the CPAD cohort, the study populations will be matched on the basis of their baseline characteristics using the propensity score matching method. This approach aims to balance the two study cohorts on baseline demographics and clinical characteristics.

The propensity score will be defined as the probability of being treated in the investigational arm (TRx-237-039) or the control arm (CPAD cohort) based on a set of baseline characteristics. If, for example, two patients, one in TRx-237-039 and another in the CPAD cohort, had the same propensity score, they would both have the same probability of being treated in investigational arm.

Propensity scores are estimated by logistic regression analyses that incorporate potential treatment predictors as independent variables, and treatment group (pooled TRx-237-039/TRx-237-080, and the CPAD cohort) as the dependent variable. The following covariates will be considered in the logistic regression model: age, sex, ApoE genotype, baseline MMSE, amyloid positivity at baseline, baseline CDR, diagnosis (MCI/AD), smoking history, education, and previous AChEI/Mem usage; final selection will depend on the availability of parameters in the CPAD database.

Age, sex, ApoE genotype, and baseline MMSE / CDR (the model must include either the baseline MMSE, or the baseline CDR; if possible, both should be included) will always be kept in the regression model, while the other covariates may be dropped depending on resulting sample size.

The feasibility of propensity score matching will be evaluated based on available sample size and descriptive results. If the matching process results in less than 50% of patients in TRx-237-039 matched to the CPAD cohort, then the selection criteria for the CPAD cohort, the covariates included in propensity score estimation, and the caliper used will be adjusted before proceeding to outcome analysis.

The distribution of baseline characteristics will be presented before and after the matching process. For baseline covariates that are not sufficiently balanced after propensity score matching, the covariates may be included in an appropriate multivariate model to adjust for those differences.

11.4.3 Handling of Missing and Incomplete Data

For both TRx-237-039 and the CPAD cohort, in the propensity score matching process, if a patient has missing information on one or more matching covariates required in the model, or missing information on both primary outcomes (ADAS-cog$_{13}$ and/or whole brain volume), the patient will be excluded from the matching. If the number of patients with data for both primary outcomes is not sufficient, matching will be conducted separately for each endpoint.

If there are insufficient ADAS-Cog$_{13}$ data available in the CPAD dataset, ADAS-Cog$_{12}$ or ADAS-Cog$_{11}$ will be used dependent on availability. ADAS-Cog$_{11}$ will in any case provide a sensitivity analysis. If there are insufficient WBV data from CPAD, other brain volumes captured in the volumetric MRI will be used with temporoparietal being the key if available. In the analysis of outcomes, if a patient has missing information on the outcome analyzed, the patient will be excluded from the analysis.

11.5 Demographic and Baseline Characteristics

Demographic variables and baseline characteristics will be summarized in tables. The summary tabulations of the number and percentage within each category (with a category for missing data) will be presented. For continuous variables, mean with standard deviation (SD), median with interquartile range (IQR), minimum and maximum values will be presented.

All data, including study eligibility and screening data (including reason for exclusion), will be listed.

12 CONFIDENTIALITY AND DATA PROTECTION

All study-related documentation is confidential, whether obtained by the investigator or provided by TauRx or their representative.

Collecting, processing of, and / or transferring data outside the European Economic Area will meet the requirements of EU Directive 2016/679 (General Data Protection Regulation), with appropriate transparency notices included in informed consent documentation. In the United States, data will be protected consistent with Health Insurance Portability and Accountability Act.

For the avoidance of doubt, this protocol does not define or describe any data protection obligations.

13 STANDARD PROCEDURES

SOPs will be adhered to for all activities relevant to the quality of the study, including protocol compliance, data collection, quality control, and data analyses and reporting.

14 REFERENCES

Al-Hilaly, Y.K., Pollack, S.J., Rickard, J.E., Simpson, M., Raulin, A.-C., Baddeley, T., Schellenberger, P., Storey, J.M.D., Harrington, C.R., Wischik, C.M., Serpell, L.C., 2018. Cysteine-independent inhibition of Alzheimer's disease-like paired helical filament assembly by leuco-methylthioninium (LMT). J Mol Biol 430, 4119–4131.

Al-Hilaly, Y.K., Pollack, S.J., Vadukul, D.M., Citossi, F., Rickard, J.E., Simpson, M., Storey, J.M.D., Harrington, C.R., Wischik, C.M., Serpell, L.C., 2017. Alzheimer's disease-like paired helical filament assembly from truncated tau protein Is independent of disulfide crosslinking. J Mol Biol 429, 3650–3665.

Alzheimer's Association, 2022. 2022 Alzheimer's disease facts and figures. Alzheimer's & Dementia 18, 700–789.

Alzheimer's Association, 2023. 2023 Alzheimer's disease facts and figures. Alzheimer's & Dementia 19, 1598–1695.

Baddeley, T.C., McCaffrey, J., M. D. Storey, J., Cheung, J.K.S., Melis, V., Horsley, D., Harrington, C.R., Wischik, C.M., 2015. Complex disposition of methylthioninium redox forms determines efficacy in tau aggregation inhibitor therapy for Alzheimer's disease. Journal of Pharmacology and Experimental Therapeutics 352, 110–118.

Bierer, L.M., Hof, P.R., Purohit, D.P., Carlin, L., Schmeidler, J., Davis, K.L., Perl, D.P., 1995. Neocortical neurofibrillary tangles correlate with dementia severity in Alzheimer's disease. Arch Neurol 52, 81–88.

Birks, J., Harvey, R., 2003. Donepezil for dementia due to Alzheimer's disease. In: Birks, Jacqueline (Ed.), Cochrane Database of Systematic Reviews. John Wiley & Sons, Ltd, Chichester, UK.

Braak, H., Braak, E., 1991. Neuropathological stageing of Alzheimer-related changes. Acta Neuropathol 82, 239–259.

Courtney, C., Farrell, D., Gray, R., Hills, R., Lynch, L., Sellwood, E., Edwards, S., Hardyman, W., Raftery, J., Crome, P., Lendon, C., Shaw, H., Bentham, P., AD2000 Collaborative Group, 2004. Long-term donepezil treatment in 565 patients with Alzheimer's disease (AD2000): randomised double-blind trial. The Lancet 363, 2105–2115.

Deiana, S., Harrington, C.R., Wischik, C.M., Riedel, G., 2009. Methylthioninium chloride reverses cognitive deficits induced by scopolamine: comparison with rivastigmine. Psychopharmacology (Berl) 202, 53–65.

Fitzpatrick, A.W.P., Falcon, B., He, S., Murzin, A.G., Murshudov, G., Garringer, H.J., Crowther, R.A., Ghetti, B., Goedert, M., Scheres, S.H.W., 2017. Cryo-EM structures of tau filaments from Alzheimer's disease. Nature 547, 185–190.

Gauthier, S., Feldman, H.H., Schneider, L.S., Wilcock, G.K., Frisoni, G.B., Hardlund, J.H., Moebius, H.J., Bentham, P., Kook, K.A., Wischik, D.J., Schelter, B.O., Davis, C.S., Staff, R.T., Bracoud, L., Shamsi, K., Storey, J.M.D., Harrington, C.R., Wischik, C.M., 2016. Efficacy and safety of tau-aggregation inhibitor therapy in patients with mild or moderate Alzheimer's disease: a randomised, controlled, double-blind, parallel-arm, phase 3 trial. The Lancet 388, 2873–2884.

Horikawa, M., Kato, Y., Sugiyama, Y., 2002. Reduced gastrointestinal toxicity following inhibition of the biliary excretion of irinotecan and its metabolites by probenecid in rats. Pharm Res 19, 1345–1353.

Lockhart, S.N., Baker, S.L., Okamura, N., Furukawa, K., Ishiki, A., Furumoto, S., Tashiro, M., Yanai, K., Arai, H., Kudo, Y., Harada, R., Tomita, N., Hiraoka, K., Watanuki, S., Jagust, W.J., 2016. Dynamic PET Measures of Tau Accumulation in Cognitively

Normal Older Adults and Alzheimer's Disease Patients Measured Using [18F] THK-5351. PLoS One 11, e0158460–e0158460.
May, J.M., Qu, Z.C., Cobb, C.E., 2004. Reduction and uptake of methylene blue by human erythrocytes. Am J Physiol Cell Physiol 286, 1390–1398.
McLachlan, A.J., Pont, L.G., 2012. Drug Metabolism in Older People--A Key Consideration in Achieving Optimal Outcomes With Medicines. J Gerontol A Biol Sci Med Sci 67A, 175–180.
Mohs, R.C., Knopman, D., Petersen, R.C., Ferris, S.H., Ernesto, C., Grundman, M., Sano, M., Bieliauskas, L., Geldmacher, D., Clark, C., Thal, L.J., 1997. Development of cognitive instruments for use in clinical trials of antidementia drugs: additions to the Alzheimer's Disease Assessment Scale that broaden its scope. The Alzheimer's Disease Cooperative Study. Alzheimer Dis Assoc Disord 11 Suppl 2, S13-21.
Mukaetova-Ladinska, E.B., Garcia-Siera, F., Hurt, J., Gertz, H.J., Xuereb, J.H., Hills, R., Brayne, C., Huppert, F.A., Paykel, E.S., McGee, M., Jakes, R., G. Honer, W., Harrington, C.R., Wischik, C.M., 2000. Staging of cytoskeletal and β-amyloid changes in human isocortex reveals biphasic synaptic protein response during progression of Alzheimer's disease. Am J Pathol 157, 623–636.
Olin, J., Schneider, L., 2002. Galantamine for Alzheimer's disease. Cochrane Database Syst Rev CD001747–CD001747.
Pontecorvo, M.J., Devous, M.D., Navitsky, M., Lu, M., Salloway, S., Schaerf, F.W., Jennings, D., Arora, A.K., McGeehan, A., Lim, N.C., Xiong, H., Joshi, A.D., Siderowf, A., Mintun, M.A., 2017. Relationships between flortaucipir PET tau binding and amyloid burden, clinical diagnosis, age and cognition. Brain aww334.
Prince, M., Wimo, A., Guerchet, M., Gemma-Claire, A., Wu, Y.-T., Prina, M., 2015. World Alzheimer Report 2015, Alzheimer's Disease International.
Reisberg, B., Doody, R., Stöffler, A., Schmitt, F., Ferris, S., Möbius, H.J., 2003. Memantine in moderate-to-severe Alzheimer's disease. N Engl J Med 348, 1333–1341.
Riedel, G., Klein, J., Niewiadomska, G., Kondak, C., Schwab, K., Lauer, D., Magbagbeolu, M., Steczkowska, M., Zadrozny, M., Wydrych, M., Cranston, A., Melis, V., Santos, R.X., Theuring, F., Harrington, C.R., Wischik, C.M., 2020. Mechanisms of anticholinesterase interference with Tau aggregation inhibitor activity in a tau-transgenic mouse model. Curr Alzheimer Res 17, 285–296.
Rosen, W.G., Mohs, R.C., Davis, K.L., 1984. A new rating scale for Alzheimer's disease. American Journal of Psychiatry 141, 1356–1364.
Schelter, B.O., Shiells, H., Baddeley, T.C., Rubino, C.M., Ganesan, H., Hammel, J., Vuksanovic, V., Staff, R.T., Murray, A.D., Bracoud, L., Riedel, G., Gauthier, S., Jia, J., Bentham, P., Kook, K., Storey, J.M.D., Harrington, C.R., Wischik, C.M., 2019. Concentration-dependent activity of hydromethylthionine on cognitive decline and brain atrophy in mild to moderate Alzheimer's disease. Journal of Alzheimer's Disease 72, 931–946.
Schneider, L.S., 2011. Treatment With Cholinesterase Inhibitors and Memantine of Patients in the Alzheimer's Disease Neuroimaging Initiative. Arch Neurol 68, 58.
Sivakumaran, S., Romero, K., Hanan, N.J., Sinha, V., Haeberlein, S.B., Gold, M., 2020. The Critical Path for Alzheimer's Disease (CPAD): Pre-competitive data sharing and generation of innovative high-impact quantitative tools to support Alzheimer's disease drug development. Alzheimer's & Dementia 16.
Ueda, K., Kato, Y., Komatsu, K., Sugiyama, Y., 2001. Inhibition of biliary excretion of methotrexate by probenecid in rats: quantitative prediction of interaction from in vitro data. J Pharmacol Exp Ther 297, 1036–43.

Wilcock, G.K., Gauthier, S., Frisoni, G.B., Jia, J., Hardlund, J.H., Moebius, H.J., Bentham, P., Kook, K.A., Schelter, B.O., Wischik, D.J., Davis, C.S., Staff, R.T., Vuksanovic, V., Ahearn, T., Bracoud, L., Shamsi, K., Marek, K., Seibyl, J., Riedel, G., Storey, J.M.D., Harrington, C.R., Wischik, C.M., 2018. Potential of low dose leuco-methylthioninium bis(hydromethanesulphonate) (LMTM) monotherapy for treatment of mild Alzheimer's disease: cohort analysis as modified primary outcome in a Phase III clinical trial. Journal of Alzheimer's Disease 61, 435–457.

Wischik, C.M., Lai, R.Y.K., Harrington, C.R., 1997. Modelling prion-like processing of tau protein in Alzheimer's disease forpharmaceutical development In Brain Microtubule Associated Proteins: Modifications in Disease, Avila J, Brandt R, Kosik KS, eds. Harwood academic publishers, Amsterdam 185–241.

Wischik, C.M., Novak, M., Edwards, P.C., Klug, A., Tichelaar, W., Crowther, R.A., 1988a. Structural characterization of the core of the paired helical filament of Alzheimer disease. Proceedings of the National Academy of Sciences 85, 4884–4888.

Wischik, C.M., Novak, M., Thogersen, H.C., Edwards, P.C., Runswick, M.J., Jakes, R., Walker, J.E., Milstein, C., Roth, M., Klug, A., 1988b. Isolation of a fragment of tau derived from the core of the paired helical filament of Alzheimer disease. Proceedings of the National Academy of Sciences 85, 4506–4510.

Wischik, C.M., Staff, R.T., Wischik, D.J., Bentham, P., Murray, A.D., Storey, J.M.D., Kook, K.A., Harrington, C.R., 2015. Tau aggregation inhibitor therapy: An exploratory Phase 2 study in mild or moderate Alzheimer's disease. Journal of Alzheimer's Disease 44, 705–720.

Xia, C., Makaretz, S.J., Caso, C., McGinnis, S., Gomperts, S.N., Sepulcre, J., Gomez-Isla, T., Hyman, B.T., Schultz, A., Vasdev, N., Johnson, K.A., Dickerson, B.C., 2017. Association of in vivo [18F]AV-1451 tau PET imaging results with cortical atrophy and symptoms in typical and atypical Alzheimer disease. JAMA Neurol 74, 427–436.

Statistical Analysis Plan

The Comparative Effectiveness of Hydromethylthionine Mesylate (HMTM) Monotherapy in Subjects with Alzheimer's Disease versus CPAD cohort based on propensity score matching

**Protocol Number: (Version Date)**	**TRx-237-080**<br>TauRx data collected from study TRx-237-039 (7.1) 16-Jun-2023 (UK: 22-AUG-2023)
**Name of Test Drug:**	Hydromethylthionine Mesylate (HMTM) previously referred to as LMTM, TRx0237
**Methodology:**	Comparative retrospective analysis
**Sponsor:**	TauRx Therapeutics Ltd.<br>395 King Street, Aberdeen AB24 5RP<br>Scotland, UK<br>[redacted]<br>TauRx Therapeutics Ltd.<br>3 Shenton Way, #21-04 Shenton House<br>Singapore 068805, Republic of Singapore
**Sponsor Representative:**	[redacted]
**Sponsor Representative:**	
**Document Date:**	18.11.2024
**Document Version:**	V1.1

TauRx / Cytel Confidential: This is a controlled document and shall not be changed, saved, copied or reproduced by any unauthorized person.

SIGNATURE PAGE

SAP Title: The Comparative Effectiveness of Hydromethylthionine Mesylate (HMTM) Monotherapy in Subjects with Alzheimer's Disease versus CPAD cohort based on propensity score matching

Sponsor: TauRx Therapeutics Ltd.

Protocol Number: TRx-237-080

Document Date/Version: 18.11.2024 v1.1

[redacted]

Cytel, Inc. Author:
Natalia Muehlemann, MD
Cytel, Inc.
675 Massachusetts Avenue
Cambridge, MA 02139

[redacted]

Cytel, Inc. Author:
Jan Priel, PhD
Cytel, Inc.
675 Massachusetts Avenue
Cambridge, MA 02139

Signature:______________________________

Date: 21-Nov-2024

TauRx / Cytel Confidential: This is a controlled document and shall not be changed, saved, copied or reproduced by any unauthorized person.

Sponsor Approva

By signing this document, I acknowledge that I have read the document and approve of the planned statistical analyses described herein. I agree that the planned statistical analyses are appropriate for this study, are in accordance with the study objectives, and are consistent with the statistical methodology described in the protocol, clinical development plan, and all applicable regulatory guidance's and guidelines.

I have discussed any questions I have regarding the contents of this document with the biostatistical author.

I also understand that any subsequent changes to the planned statistical analyses, as described herein, may have a regulatory impact and/or result in timeline adjustments. All changes to the planned analyses will be described in the analysis report.

Sponsor Signatory:
Prof. Bjoern Schelter
Chief Analytics Officer

Signature: [redacted]

Date: 20-NOV-2024

Sponsor Signatory:
Kath Martin

Head of Data Science

Signature [redacted]

Date: 20-NOV-2024

Sponsor Signatory:
Prof. Claude Wischik

TauRx CEO

Signature [redacted]

Date: 20 NOV 2024

TauRx / Cytel Confidential: This is a controlled document and shall not be changed, saved, copied or reproduced by any unauthorized person.

MODIFICATION HISTORY

Current Version	Date	Amended by	Summary of Changes from previous version	Reason

TauRx / Cytel Confidential: This is a controlled document and shall not be changed, saved, copied or reproduced by any unauthorized person.

TABLE OF CONTENTS

TABLE OF CONTENTS 5

ABBREVIATIONS 6

1. INTRODUCTION AND OBJECTIVES 7

1.1 INTRODUCTION 7
1.2 OBJECTIVES 8

2. STUDY DESIGN AND DATA SOURCES 9

2.1 STUDY DESIGN 9
2.2 STUDY TRX-237-039 9
2.3 CPAD COHORT 10
2.4 SELECTION CRITERIA FOR CPAD AD COHORT 12

3. VARIABLES 14

3.1 TREATMENT GROUP 14
3.2 OUTCOMES 14
3.3 COVARIATES 15

4. STATISTICAL METHODS 18

4.1 COMPUTING ENVIRONMENT 18
4.2 DESCRIPTIVE ANALYSIS 18
4.3 MISSING DATA 18
4.4 PROPENSITY SCORE MATCHING 19
4.5 ANALYSIS OF OUTCOMES 20

5. LIMITATIONS OF THE RESEARCH METHODS 22

6. REFERENCES 23

TauRx / Cytel Confidential: This is a controlled document and shall not be changed, saved, copied or reproduced by any unauthorized person.

ABBREVIATIONS

Abbreviation	Definition
AChEI	Acetylcholinesterase inhibitor
AChMem	Acetylcholinesterase inhibitor / memantine
AD	Alzheimer's disease
ADAS-cog$_{11}$	Alzheimer's Disease Assessment Scale 11-item version
ADAS-cog$_{13}$	Alzheimer's Disease Assessment Scale 13-item version
ADCS-ADL$_{23}$	Alzheimer's Disease Cooperative Study – Activities of Daily Living (23-item)
ApoE	Apolipoprotein E
CDISC	Clinical Data Interchange Standards Consortium
CDR	Clinical Dementia Rating
CPAD	Critical Path for Alzheimer's Disease
CSR	Clinical study report
E-MITT	Efficacy modified intention-to-treat
HMTM	Hydromethylthionine mesylate
IQR	Interquartile range
ITT	Intention-to-treat
LDL	Low-density-lipoprotein
MCI	Mild cognitive impairment due to Alzheimer's disease
MDD	Major depressive disorder
MMSE	Mini-Mental Status Examination
MTC	Methylthioninium chloride
PET	Positron emission tomography
SAP	Statistical analysis plan
SD	Standard deviation
WBV	Whole Brain Volume

TauRx / Cytel Confidential: This is a controlled document and shall not be changed, saved, copied or reproduced by any unauthorized person.

1. INTRODUCTION AND OBJECTIVES

1.1 Introduction

An unmet need exists to develop new medications for Alzheimer's disease (AD) that more directly modify the underlying disease pathology and offer longer-term and greater efficacy. HMTM (hydromethylthionine mesylate), is believed to have the potential to confer benefits over existing treatments for AD due to its ability to affect the process of tau aggregation responsible for the underlying neurofibrillary pathology of AD. Available nonclinical and clinical evidence supports the clinical evaluation of HMTM in AD.

Results from two phase 3 studies of HMTM on AD subjects (TRx-237-005, TRx-237-015) suggest that HMTM 8 mg/day given as monotherapy may be effective in delaying progression of mild to moderate AD on co-primary clinical efficacy endpoints, ADAS-cog_{11} and ADCS-ADL_{23}, and also on magnetic resonance imaging (MRI) measures of progression of brain atrophy and ^{18}F-fluorodeoxyglucose positron emission tomography (^{18}F-FDG-PET) measures of impairment in neuronal metabolic function [1, 2].

Study TRx-237-039 is a randomized, double-blind, controlled, three-arm, 12-month, safety and efficacy study of HMTM monotherapy in subjects with AD followed by a 12-month open-label treatment [3]. In the 12-month double-blind period, eligible patients were randomly assigned either HMTM 16 mg/day, HMTM 8 mg/day, or control. In order to provide some degree of urinary coloration in what was originally a "true" placebo control group, a small amount of methylthioninium chloride (MTC), 4 mg, was introduced to placebo treatment kits (one tablet on two intermittent occasions twice per week, i.e., 8 mg/week). This amount of MTC had been anticipated to be without activity, consistent with results of a prior Phase 2 trial and estimated plasma half-life. Lack of "true" placebo arm in TRx-237-039 presents a limitation in providing evidence of the effectiveness of HMTM. Because of the atypical biphasic pharmacokinetics of the active moiety, hydromethylthionine (HMT) plasma levels increased over the first 12 months followed by return to the 1-month levels at 24 months whether HMT was delivered as MTC to HMTM [popPK report]. As a result of this, plasma levels at 12 months in subjects receiving MTC 4 mg twice weekly were within the range of doses that have been shown to have statistically significant symptomatic activity in a standard mouse model based on reversal of a scopolamine-induced learning deficit [4]. Therefore, study TRx-237-039 lacked a true untreated control arm and symptomatic activity partially confounded the interpretation of the true effect of HMTM as a disease-modifying treatment for AD. For this reason, this treatment arm is referred to as "control" rather than "placebo" in this trial.

The Critical Path Institute (C-Path) is a nonprofit organization operating as an independent, public-private partnership with the U.S. FDA, created under the auspices of the FDA's Critical Path Initiative program in 2005 [5]. Founded in 2008, the Critical Path for Alzheimer's Disease (CPAD) database contains, but is not limited to, demographic information, Apoε4 genotype, concomitant medications, and cognitive

TauRx / Cytel Confidential: This is a controlled document and shall not be changed, saved, copied or reproduced by any unauthorized person.

scales (MMSE and ADAS-Cog). Limited AD biomarker data (biofluids, tau or amyloid positron emission tomography (PET), EEG data) are also available.

Both CPAD database and TRx-237-039 reported key baseline demographic and clinical characteristics and efficacy outcomes, Alzheimer's Disease Assessment Scale, 13-item version (ADAS-cog13) and Whole Brain Volume (WBV). To address the lack of a true control with regards to study TRx-237-039, this analysis will use the CPAD cohort as an external control for experimental arm of TRx-237-039.

All data has been remapped to a common data standard (CDISC SDTM v3.1.2) such that all the data can be analyzed across all studies.

This document describes the statistical analysis of comparing the efficacy between the investigational study arm (HMTM 16mg/day) of TRx-237-039 trial and the CPAD cohort based on propensity score matching.

1.2 Objectives

The objective of this analysis is to evaluate the effect of HMTM 16mg/day from study TRx-237-039 on ADAS-cog13 (or a subscale if ADAS-cog13 is not available in sufficient quantities, see below) and WBV (or other brain imaging marker if WBV is not available in sufficient quantities, see below), compared to the propensity score-matched CPAD cohort.

This statistical analysis plan (SAP) is designed to outline the methods to be used in the analysis of TRx-237-039 and CPAD data to answer the study objective. Populations for analysis, variables to be used, and statistical methods are provided.

TauRx / Cytel Confidential: This is a controlled document and shall not be changed, saved, copied or reproduced by any unauthorized person.

2. STUDY DESIGN AND DATA SOURCES

2.1 Study design

This analysis is a retrospective analysis of the following:

- Retrospective analysis of patients in already completed TRx-237-039 trial
- Retrospective analysis of CPAD database

Participants from the CPAD cohort will be matched to the data from the HMTM 16mg/day arm of TRx-237-039 trial only based on propensity score matching (method outlined in Section 4.4).

This analysis plan was finalized prior to any access of the CPAD datasets.

2.2 Study TRx-237-039

TRx-237-039 is a two-phase outpatient study of HMTM administered as monotherapy in an intended 500 subjects (450 under protocol version 5.0 or higher; 598 were actually randomized under all versions of the protocol) with early to mild-moderate AD: a randomized, double-blind, controlled, 52-week treatment phase followed by a 52-week open-label treatment phase that represents a modified delayed start of treatment. Subjects for whom legally acceptable informed consent was obtained and who were found eligible on the basis of screening evaluations, were randomly assigned at baseline to receive either HMTM 16 mg/day, HMTM 8 mg/day, or control (i.e., placebo/MTC) (4:1:4, at the study level); the drug supplies for the control group included tablets containing a urinary colorant (MTC), 4 mg, dosed at an average frequency of two tablets per week.

The primary treatment group comparison during the double-blind treatment phase was between HMTM 16 mg/day and control. Following completion of the 52-week treatment phase, all subjects (regardless of randomized treatment assignment or response) continued open-label treatment with HMTM 16 mg/day for a further 52 weeks.

TauRx / Cytel Confidential: This is a controlled document and shall not be changed, saved, copied or reproduced by any unauthorized person.

Figure 1. Schematic of study design of TRx-237-039 (Protocol version 5.0+)

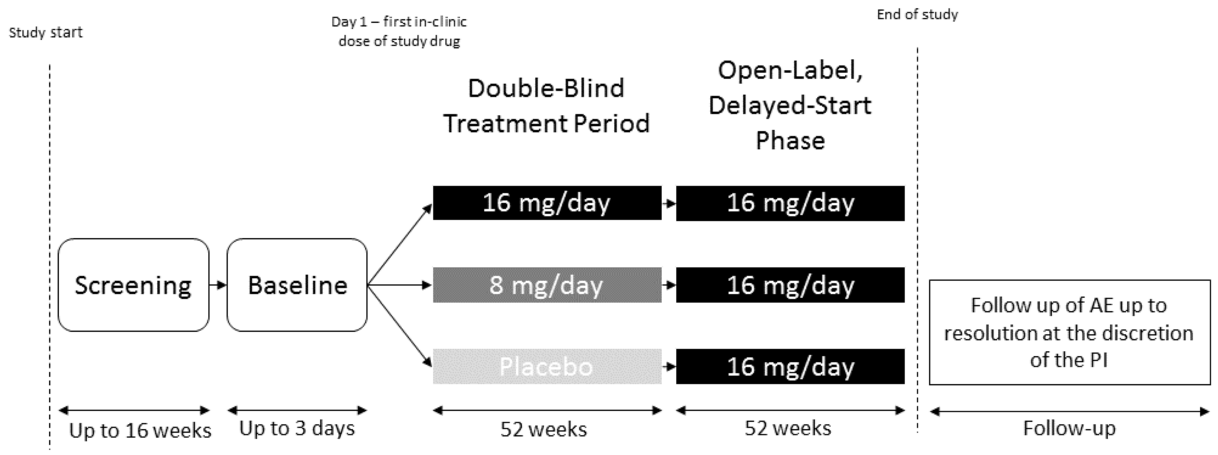

In the current analysis, outcome data collected during the 52-week double-blind treatment phase, as well as during the 104-week period (double-blind + open-label) will be used. This data will only consist of those randomized to 16mg/day at the start of the trial; these subjects were on the 16mg/day for the entire 104 weeks. The primary comparison is at 78 weeks, assuming sufficiently many subjects had data for 78 weeks in CPAD. If not the analysis will revert to 52 weeks.

TRx-237-039 data was collected and coded following the Clinical Data Interchange Standards Consortium (CDISC) standards (version 1.4; implementation guides v3.2). Coding details are described in the Data Management Plan of the trial.

2.3 CPAD Cohort

The CPAD database includes patient-level data from 12,811 patients across 36 clinical trials of AD and MCI. The database contains, but is not limited to, demographic information, ApoE4 genotype, concomitant medications, and cognitive scales (MMSE and ADAS-Cog). Limited treatment-arm data and limited AD biomarker data (biofluids, tau or amyloid positron emission tomography (PET), EEG data) is available. All data has been remapped

TauRx / Cytel Confidential: This is a controlled document and shall not be changed, saved, copied or reproduced by any unauthorized person.

to a common data standard (CDISC SDTM v3.1.2) such that all the data can be analyzed across all studies. All data are fully de-identified.

Table 1. Highlight of domains captured in CPAD AD database

CDISC Domain	Contents
DM	Age<br>Sex<br>Race<br>Ethnicity<br>Country
CM	**Acetylcholinesterase Inhibitors<br>**Memantine<br>**General Medications
AE	***Event<br>Severity<br>Duration
MH	General Medical History<br>AD\MCI Diagnosis
VS	SBP, DBP<br>Heart Rate<br>Temperature<br>Weight, Height<br>BMI<br>Respiratory Rate
QS	ADAS-Cog<br>MMSE<br>ADCS-ADL<br>NPI<br>CDR *(Limited Number of studies)*<br>Others as collected may be present
LB	All labs collected, mapped to SDTM. Fluid biomarkers *(limited data available)*
**PF**	*ApoE Genotype
**NV**	MRI *(limited data available)*<br>Amyloid and FDG – PET *(limited data available)*
**XD**	Information about Alzheimer's Disease and Symptom duration

TauRx / Cytel Confidential: This is a controlled document and shall not be changed, saved, copied or reproduced by any unauthorized person.

**ApoE = Apolipoprotein E; **Memantine and AChEi drug names are standardized in the CMDECOD field. All other drug names are provided in the verbatim terminology supplied to CPAD.*

2.4 Selection criteria for CPAD AD cohort

The inclusion and exclusion criteria for TRx-237-039 are available in the respective study protocol [3].

To create a group of patients from CPAD - who are similar to the patients in the HMTM 16 mg/day arm of TRx-237-039, the following inclusion and exclusion criteria from TRx-237-039 trial will be applied to “enroll” (select) patients from CPAD database prior to propensity score matching:

Inclusion criteria:

1) Probable AD or MCI due to AD (MCI-AD; MCI-AD is referred to as MCI in the sections below)
2) PET scan positive for amyloid in MCI-AD; PET scan positivity for amyloid in the CPAD population is not needed in mild/moderate AD as this severity level is sufficient to replace the need for a PET scan in identifying patients who are anticipated to decline clinically and is not generally used trials in mild/moderate AD. If data availability for PET scan in the CPAD cohort is limited, the main analysis will be conducted without this criterion, but a sensitivity analysis including this criterion will be performed.
3) MMSE score of 16 – 27 at screening
4) Global CDR score of 0.5 to 2 at screening (if 0.5, including a score of > 0 in one of the functional domains: Community Affairs, Home and Hobbies, or Personal Care). If data availability for CDR in CPAD cohort is limited, this criterion will be dropped.
5) Outpatients below 90 years at screening (patients residing in hospitals or moderate to high dependency continuous care facilities are excluded)

Exclusion criteria:

1) Significant central nervous system (CNS) disorder other than probable AD or MCI-AD, *e.g.*, Lewy body dementia, Parkinson’s disease, multiple sclerosis, progressive supranuclear palsy, hydrocephalus, Huntington’s disease, any condition directly or indirectly caused by Transmissible Spongiform, Encephalopathy (TSE), Creutzfeldt-Jakob Disease (CJD), variant Creutzfeldt-Jakob Disease (vCJD), or new variant Creutzfeldt-Jakob Disease (nvCJD)

TauRx / Cytel Confidential: This is a controlled document and shall not be changed, saved, copied or reproduced by any unauthorized person.

2) Significant neuroimaging findings e.g. significant intracranial focal or vascular pathology seen on brain MRI including but not limited to:
   a) Large confluent white matter hyperintense lesions (*i.e.*, Fazekas score of 3)
   b) Other focal brain lesions judged clinically relevant by the investigator
   c) Evidence of a prior or current macrohemorrhage
3) Mental Disorders such as
   a) Current major depressive disorder (MDD)
   b) History of schizophrenia
   c) Other psychotic disease, bipolar disorder within past 5 years from the baseline

      Substance (including alcohol) related disorders within past 5 years from the baseline
4) History of any conditions that may be impacting cognitive function:
   a) Cerebrovascular accident
   b) Transient ischemic attack
   c) Significant head injury, for example, associated loss of consciousness, skull fracture or persisting cognitive impairment
   d) Other unexplained or recurrent loss of consciousness
5) Diagnosed epilepsy (a single prior seizure >6 months prior to Screening is considered acceptable)
6) Treatment currently or within 30 days before the baseline with:
   a) Acetylcholinesterase inhibitor (AChEI) and/or memantine
   b) Clozapine (other antipsychotics are allowed if they have not been initiated within 90 days before baseline)
   c) Carbamazepine, primidone, valproate
   d) Drugs for which there is a warning or precaution in the labeling about methemoglobinemia at approved doses (e.g., dapsone, local anesthetics such as benzocaine used chronically, primaquine and related antimalarials)

   However, if applying this criterion results in small cohort, it will be removed, and this will be acknowledged as a study limitation.

TauRx / Cytel Confidential: This is a controlled document and shall not be changed, saved, copied or reproduced by any unauthorized person.

3. VARIABLES

3.1 Treatment group

Patients selected from CPAD database will be considered as control.

For TRx-237-039, only subjects randomized to HMTM 16mg/day arm at the start of the study will be analyzed.

3.2 Outcomes

Two efficacy outcomes, ADAS-cog_{13} and WBV (whole brain volume) will be analyzed as primary endpoints. As secondary outcomes, CDR sum of boxes as well as MMSE will be analysed at weeks 52 and 104. CDR global scale as well as neurofilament light chain (NfL) will be analyzed as exploratory, assuming a linear transformation of the NfL data can ensure comparability (NfL is impacted by the actual kits that are used to analyze, which is why a transformation will likely be needed).

The ADAS-cog was designed to evaluate the severity of cognitive of AD [6]. The ADAS-cog_{11} is the cognitive subscale of the ADAS, originally proposed with 11 items (domains: memory, praxis, orientation, and language), resulting in scores that range from 0 to 70, with higher numbers indicating greater impairment. For the ADAS-cog_{13}, two additional items have been added (in domain Memory: Delayed Word Recall, and as a new domain Attention: Number Cancellation) to provide additional sensitivity to change in cognition at earlier stages of the disease, resulting in a maximal score of 85.

In TRx-237-039, ADAS-cog_{13} was assessed at baseline, 13, 26, 39, 52, 78, and 104 weeks. ADAS-cog_{11} as well and ADAS-cog_{12} can be derived from the 13-item scale. ADAS-cog_{13} collected at similar timepoints in CPAD control will be used for the control arm. If there is insufficient ADAS-cog_{13} data from CPAD, ADAS-cog_{12} or ADAS-cog_{11} will be used instead to compare the two cohorts; dependent on which scale is available or can be derived and compared meaningfully with ADAS-cog_{12} being the preference. ADAS-cog_{11} will be a sensitivity in case it is not used as a primary. In TRx-237-039, change in brain volumes was analyzed using the brain boundary shift integral. In TRx-237-039, WBV was assessed at screening, 13, 26, 39, 52, 78, and 104 weeks, or early termination. WBV collected at similar timepoints in CPAD control will be used for the control arm. If there is insufficient WBV data from CPAD, other brain volumes captured in the volumetric MRI will be used with temporoparietal being the key if available. MMSE and CDR sum of boxes will be analyzed as secondary endpoints; CDR sum of boxes is combining cognitive as well as functional aspects.

TauRx / Cytel Confidential: This is a controlled document and shall not be changed, saved, copied or reproduced by any unauthorized person.

3.3 Covariates

The variables listed in Table 2 will be considered as covariates in the propensity score matching (Section 4.4). More details on these variables can be found in the SAP of TRx-237-039 [7] and CPAD.

Table 2. Covariates to be considered in propensity score matching

Variable	Type	Categories
Age	Continuous	-
Sex	Binary	Male, Female
Smoking history	Binary	Yes (Past or current smoker)<br>No (Non-smoker)
Education*	Binary	Yes (Had post-secondary education)<br>No (Did not have post-secondary education)
ApoE genotype	Binary	Yes (Presence of ε4 allele)<br>No (Absence of ε4 allele)
Baseline MMSE	Categorical	Moderate (16-19)<br>Mild (20-25)<br>Very mild (26-27)
Baseline CDR	Categorical	Questionable impairment (0.5)<br>Mild impairment (1)<br>Moderate impairment (2)<br>Severe impairment (3) (exclusionary in TRx-237-039 and therefore not used)
Previous AchMem usage	Binary	Yes, No

* In TRx-237-039, education is coded from 1-6, with 1-3 being no post-secondary education, and 4-6 being having post-secondary education

Abbreviations: ApoE, Apolipoprotein E; AchMem, acetylcholinesterase inhibitor / memantine; CDR, Clinical Dementia Rating; MMSE, Mini-Mental Status Examination; MCI-AD, Mild Cognitive Impairment due to Alzheimer's disease

TauRx / Cytel Confidential: This is a controlled document and shall not be changed, saved, copied or reproduced by any unauthorized person.

Rationale for selected covariates:

Age

Age is a known risk factor for the development of AD. The disease incidence is well documented to increase with advanced age [8, 9]. Riedel et al. 2016 asserted that the incidence rises exponentially after the sixth decade of life [10].

Sex

The incidence of AD is equal between males and females initially but with advanced age, the incidence is greater in females [11, 12]. Females are known to have a higher prevalence of AD, which may be partially attributable to their longevity [9,10]. Based on neuroimaging, it has been suggested that the preclinical AD phase in females coincides with the perimenopausal transition in middle age [13]. It has been hypothesized that estrogens may be protective against the mitochondrial toxicity of amyloid-beta so that the decline in estrogens associated with female aging removes that protection [14].

Baseline Mini-Mental Status Examination (MMSE)

While a meta-analysis to evaluate the MMSE for early detection of mild cognitive impairment did not show evidence that it has reliability as a stand-alone test, it is still one of the best known and most commonly utilized assessments [15]. In order to compare cohorts and measure change of time, it may appropriately be considered as part of the totality of the evidence.

Apolipoprotein E (ApoE) genotype

ApoE, a critical cholesterol transporting protein, has three isoforms in humans: ApoE2, ApoE3 and ApoE4. The last of these, ApoE4, is associated with elevated plasma cholesterol and low-density-lipoprotein (LDL); this isoform predisposes carriers to both cardiovascular disease and neurodegenerative disease [16-18]. ApoE4 is more commonly found in persons of African descent, but even though the isoform is less common in persons of European descent, within those populations, the risk of cognitive decline is greater when it is present [19]. ApoE4 is associated with dose-dependent increases in amyloid burden in persons of any race/ethnicity during prodromal stages of AD [10]. ApoE4 is associated with a reduction in the ability to clear amyloid beta, an increase in tau accumulation and an increase in the production of reactive oxygen species [10]. According to Riedel et al. 2016, ApoE4 has its highest effect on risk for persons as they are in the early part of the eight decades of life, but the risk reduces after age 85 years; "The ApoE4 gene dose effect on risk and age of AD onset indicates that ApoE4 dramatically increased risk of AD and an earlier age of onset (p.9)." Persons of either sex who have two copies of ApoE4 have a 25-fold increase in risk with earlier age of disease onset when compared with persons who carry the Apo2 or Apo3 alleles [10]. While carriers of the E2/E3 heterozygote alleles appear to be protected against AD, the E2 protection seems to extend to E2 homozygous females but not to E2 homozygous males [10].

TauRx / Cytel Confidential: This is a controlled document and shall not be changed, saved, copied or reproduced by any unauthorized person.

Smoking history

Studies have shown that there is a higher risk of AD in lifetime tobacco users, especially in those who do not have ApoE4 [9, 21-24]. Current tobacco use is associated with a pooled relative risk of 1.79 (95% CI, 1.43-2.23) [8].

Education

Education levels of high school or below have been associated with an increased risk of dementia and AD [8, 21, 24].

Baseline Clinical Dementia Rating (CDR)

CDR quantifies disease severity which is linked to cognitive/functional decline and disease progression.

While the gender effect was most pronounced in ApoE4 female carriers, all women diagnosed with mild cognitive impairment by baseline CDR had cognitive decline progress at faster rates than men.

Among heterozygotes, female E3/E4 carriers show faster age-related decline and greater deterioration of cognition than males with the same allele pattern [10]. Females who carry E3/E4 or E4/E4 and who are found to have MCI, have more rapid cognitive decline but a longer survival rate [10].

Previous acetylcholinesterase inhibitor / memantine (AchMem) usage

This covariate is being included to separate treatment-naïve patients from those who had clinical symptoms that were of sufficient severity that a physician thought it was appropriate to start treatment for cognitive impairment. AchMems are standard therapeutics for mild cognitive impairment and AD [25].

TauRx / Cytel Confidential: This is a controlled document and shall not be changed, saved, copied or reproduced by any unauthorized person.

4. STATISTICAL METHODS

4.1 Computing Environment

Data for TRx-237-039 and the CPAD cohort will be stored in a secure environment. Access to the data will be through a secure connection. Data will be checked for consistency against clinical study report (CSR) of TRx-237-039 and publications of the CPAD cohort where available. Data management and statistical analyses will be performed using SAS® statistical software (Version 9.4 or higher) and/or R, unless otherwise noted.

4.2 Descriptive analysis

All patient characteristics and outcome variables (Section 3) will be summarized descriptively for unmatched and matched cohorts.

Tabulations will be produced for appropriate baseline characteristics and outcome variables. For categorical variables, summary tabulations of the number and percentage within each category (with a category for missing data) will be presented. For continuous variables, mean with standard deviation (SD), median with interquartile range (IQR), minimum and maximum values will be presented. The difference between the cohorts will be assessed by t-test for continuous variables and chi-squared test for categorical variables. P value less than 0.05 will denote statistical significance.

4.3 Missing data

For both TRx-237-039 and the CPAD cohort, in the propensity score matching process, if a patient has missing information on one or more matching covariates required in the model, or missing information on both primary outcomes (ADAS-cog_{13} and/or whole brain volume), the patient will be excluded from the matching. If the number of patients with data for both primary outcomes is not sufficient, matching will be conducted separately for each endpoint.

If there are insufficient ADAS-Cog_{13} data available in the CPAD dataset, ADAS-Cog_{12} or ADAS-Cog_{11} will be used dependent on availability. ADAS-Cog_{11} will in any case provide a sensitivity analysis. If there are insufficient WBV data from CPAD, other brain volumes captured in the volumetric MRI will be used with temporoparietal being the key if available. In the analysis of outcomes, if a patient has missing information on the outcome analyzed, the patient will be excluded from the analysis.

TauRx / Cytel Confidential: This is a controlled document and shall not be changed, saved, copied or reproduced by any unauthorized person.

4.4 Propensity score matching

To control for confounders in the comparison of treatments used in TRx-237-039 versus the CPAD cohort, the study populations will be matched on their baseline characteristics using the propensity score matching method. This approach aims to balance the two study cohorts on baseline demographics and clinical characteristics.

For TRx-237-039, both intention-to-treat (ITT) population and efficacy modified intention-to-treat (E-MITT) population will be used in the matching, with the E-MITT population considered as the primary analysis population and the ITT population as sensitivity analysis population. For the CPAD cohort, to maximize the resulting sample size, the matching process of the primary analysis will include all patients who met the selection criteria described in Section 2.4 (unless otherwise specified).

Table 3. Populations to be used in propensity score matching

	TRx-237-039	CPAD
Primary analysis	E-MITT	Patients who met the selection criteria described in Section 2.4
Sensitivity analysis	ITT	Patients who met the selection criteria described in Section 2.4

Abbreviations: E-MITT, efficacy modified intention-to-treat; ITT, intention-to-treat

The propensity score will be defined as the probability of being treated in either investigational arm TRx-237-039 or the control arm (CPAD cohort) based on a set of baseline characteristics [26]. If, for example, two patients, one in TRx-237-039 and another in the CPAD cohort, had the same propensity score, they would both have the same probability of being treated in investigational arm.

Propensity scores are estimated by logistic regression analyses that incorporate potential treatment predictors as independent variables, and treatment group (TRx-237-039 and the CPAD cohort) as the dependent variable. The following covariates will be considered in the logistic regression model: age, sex, ApoE genotype, baseline MMSE, baseline CDR, smoking history, education, and previous AchMem usage (Section 3.3). Age, sex, ApoE genotype, and baseline MMSE / CDR (the model must include either the baseline MMSE, or the baseline CDR; if possible, both should be included) will always be kept in the regression model, while the other covariates may be dropped depending on resulting sample size. The optimal fixed ratio propensity score matching method with logit of the propensity score as distance will be performed. A caliper of 0.10 (increasing up to 0.20 depending on resulting sample sizes) of the standard deviation of the estimated logit will be used to select the matched samples. The fixed ratio is chosen to be 1:1.

TauRx / Cytel Confidential: This is a controlled document and shall not be changed, saved, copied or reproduced by any unauthorized person.

The feasibility of propensity score matching will be evaluated based on available sample size and descriptive results. If the matching process results in less than 50% of patients in TRx-237-039 matched to the CPAD cohort, then the selection criteria for CPAD cohort, the covariates included in propensity score estimation, and the caliper used will be adjusted before proceeding to outcome analysis.

The distribution of baseline characteristics will be presented before and after the matching process. Standardized differences in the variables between the treated and control groups after matching will be used to assess the quality of matching. For baseline covariates that are not sufficiently balanced after propensity score matching, the covariates may be included in an appropriate multivariate model to adjust for those differences.

4.5 Analysis of outcomes

All primary and secondary outcomes (Section 3.2) will be analyzed using a linear model, including changes from baseline to 52, 78, and 104 weeks as the dependent variable, and treatment group as the independent variable. Week 78 will be used for the primary, unless insufficient data in which case week 52 will be used.

Treatment difference between the treatment group and the untreated group will be presented as the coefficient for treatment group variable with 95% confidence intervals. Considering the potential differences in disease progression between AD and MCI, patients with AD and patients with MCI from TRx-237-039 will also be analyzed separately for the comparison between HMTM 16 mg/day in TRx-237-039 and the untreated group in CPAD (Table 44).

TauRx / Cytel Confidential: This is a controlled document and shall not be changed, saved, copied or reproduced by any unauthorized person.

Table 4. Summary of analyses to be conducted

Outcome	Time period	Population	Diagnosis	Population from TRx-237-039	Population from CPAD cohort
Change from baseline in ADAS-cog$_{13}$ / Whole brain volume / CDR sum of boxes and MMSE CDR global and NfL as exploratory only for E-MITT	52, 78 (primary analysis) and 104 weeks	Primary analysis: TRx-237-039 E-MITT population *propensity score-matched to* CPAD patients who met the selection criteria	AD or MCI diagnosis	Matched patients who were randomized to HMTM 16mg/day	All matched patients
			MCI diagnosis in TRx-237-039	Matched MCI patients who were randomized to HMTM 16 mg/day	Patients who are matched with those MCI patients receiving HMTM 16 mg/day in TRx-237-039
			AD diagnosis in TRx-237-039	Matched AD patients who were randomized to HMTM 16 mg/day	Patients who are matched with those AD patients receiving HMTM 16 mg/day in TRx-237-039
		Sensitivity analysis: TRx-237-039 ITT population *propensity score-matched to* CPAD patients who met the selection criteria	AD or MCI diagnosis	Matched patients who were randomized to HMTM 16mg/day	All matched patients
			MCI diagnosis in TRx-237-039	Matched MCI patients who were randomized to HMTM 16 mg/day	Patients who are matched with those MCI patients receiving HMTM 16 mg/day in TRx-237-039
			AD diagnosis in TRx-237-039	Matched AD patients who were randomized to HMTM 16 mg/day	Patients who are matched with those AD patients receiving HMTM 16 mg/day in TRx-237-039

From each of the matched patient samples, the following matched pairs of patients will be included for each analyses specified: first, matched patients from TRx-237-039 will be selected based on diagnosis (AD, MCI, or AD/MCI), then patients from CPAD who were matched to those selected patients in TRx-237-039 will be selected

Abbreviations: AD, Alzheimer's disease; ADAS-cog$_{13}$, Alzheimer's Disease Assessment Scale 13-item version; HMTM, hydromethylthionine mesylate; MCI, mild cognitive impairment due to AD; MTC, Methylthioninium Chloride; ITT, intention-to-treat; E-MITT, efficacy modified intention-to-treat

TauRx / Cytel Confidential: This is a controlled document and shall not be changed, saved, copied or reproduced by any unauthorized person.

5. LIMITATIONS OF THE RESEARCH METHODS

This study uses clinical trial data from different RCTs conducted over different time periods. In addition, study periods of TRx-237-039 overlapped with the COVID-19 pandemic, which may have an impact on treatment decision making and outcomes. CPAD database comprises several studies, and CDR data were available only from some studies. Amyloid and FDG – PET data in CPAD database are also limited.

TauRx / Cytel Confidential: This is a controlled document and shall not be changed, saved, copied or reproduced by any unauthorized person.

6. REFERENCES

1. Gauthier, S., et al., *Efficacy and safety of tau-aggregation inhibitor therapy in patients with mild or moderate Alzheimer's disease: a randomised, controlled, double-blind, parallel-arm, phase 3 trial.* Lancet, 2016. **388**(10062): p. 2873-2884.

2. Wilcock, G.K., et al., *Potential of Low Dose Leuco-Methylthioninium Bis(Hydromethanesulphonate) (LMTM) Monotherapy for Treatment of Mild Alzheimer's Disease: Cohort Analysis as Modified Primary Outcome in a Phase III Clinical Trial.* J Alzheimers Dis, 2018. **61**(1): p. 435-457.

3. TauRx Therapeutics Ltd., *CLINICAL STUDY PROTOCOL: Randomized, Double-Blind, Placebo-Controlled, Three-Arm, 12-Month, Safety and Efficacy Study of Hydromethylthionine Mesylate (LMTM) Monotherapy in Subjects with Alzheimer's Disease Followed by a 12-Month Open-Label Treatment*. 2021.

4. D*eiana, S., Harrington, C. R., Wischik, C. M., & Riedel, G. (2009). Methylthioninium chloride reverses cognitive deficits induced by scopolamine: comparison with rivastigmine. Psychopharmacology, 202(1), 53–65.*

5. Critical Path Initiative program in 2005. https://c-path.org/about/

6. Rosen, W.G., R.C. Mohs, and K.L. Davis, *A new rating scale for Alzheimer's disease.* Am J Psychiatry, 1984. **141**(11): p. 1356-64.

7. TauRx Therapeutics Ltd., *Statistical Analysis Plan: Randomized, Double-Blind, Placebo-Controlled, Three-Arm, 12-Month, Safety and Efficacy Study of Hydromethylthionine Mesylate (LMTM) Monotherapy in Subjects with Alzheimer's Disease Followed by a 12-Month Open-Label Treatment.* 2023.

8. Qiu, C., M. Kivipelto, and E. von Strauss, *Epidemiology of Alzheimer's disease: occurrence, determinants, and strategies toward intervention.* Dialogues Clin Neurosci, 2009. **11**(2): p. 111-28.

9. R, A.A., *Risk factors for Alzheimer's disease.* Folia Neuropathol, 2019. **57**(2): p. 87-105.

10. Riedel, B.C., P.M. Thompson, and R.D. Brinton, *Age, APOE and sex: Triad of risk of Alzheimer's disease.* J Steroid Biochem Mol Biol, 2016. **160**: p. 134-47.

11. Barnes, L.L., et al., *Gender, cognitive decline, and risk of AD in older persons.* Neurology, 2003. **60**(11): p. 1777-81.

TauRx / Cytel Confidential: This is a controlled document and shall not be changed, saved, copied or reproduced by any unauthorized person.

12. Ruitenberg, A., et al., *Incidence of dementia: does gender make a difference?* Neurobiol Aging, 2001. **22**(4): p. 575-80.

13. Mosconi, L., et al., *Sex differences in Alzheimer risk: Brain imaging of endocrine vs chronologic aging.* Neurology, 2017. **89**(13): p. 1382-1390.

14. Viña, J. and A. Lloret, *Why women have more Alzheimer's disease than men: gender and mitochondrial toxicity of amyloid-beta peptide.* J Alzheimers Dis, 2010. **20 Suppl 2**: p. S527-33.

15. Arevalo-Rodriguez, I., et al., *Mini-Mental State Examination (MMSE) for the early detection of dementia in people with mild cognitive impairment (MCI).* Cochrane Database Syst Rev, 2021. **7**(7): p. Cd010783.

16. Liu, C.C., et al., *Apolipoprotein E and Alzheimer disease: risk, mechanisms and therapy.* Nat Rev Neurol, 2013. **9**(2): p. 106-18.

17. Mahley, R.W., K.H. Weisgraber, and Y. Huang, *Apolipoprotein E: structure determines function, from atherosclerosis to Alzheimer's disease to AIDS.* J Lipid Res, 2009. **50 Suppl**(Suppl): p. S183-8.

18. Mayeux, R., et al., *The apolipoprotein epsilon 4 allele in patients with Alzheimer's disease.* Ann Neurol, 1993. **34**(5): p. 752-4.

19. Kuller, L.H., et al., *Relationship between ApoE, MRI findings, and cognitive function in the Cardiovascular Health Study.* Stroke, 1998. **29**(2): p. 388-98.

22. Chang, R.C., et al., *Neuropathology of cigarette smoking.* Acta Neuropathol, 2014. **127**(1): p. 53-69.

21. Henderson, A.S., *The risk factors for Alzheimer's disease: a review and a hypothesis.* Acta Psychiatr Scand, 1988. **78**(3): p. 257-75.

22. Soininen, H. and O.P. Heinonen, *Clinical and etiological aspects of senile dementia.* Eur Neurol, 1982. **21**(6): p. 401-10.

23. Durazzo, T.C., N. Mattsson, and M.W. Weiner, *Smoking and increased Alzheimer's disease risk: a review of potential mechanisms.* Alzheimers Dement, 2014. **10**(3 Suppl): p. S122-45.

24. Jonaitis, E., et al., *Cognitive activities and cognitive performance in middle-aged adults at risk for Alzheimer's disease.* Psychol Aging, 2013. **28**(4): p. 1004-14.

TauRx / Cytel Confidential: This is a controlled document and shall not be changed, saved, copied or reproduced by any unauthorized person.

25. Vecchio, I., et al., *The State of The Art on Acetylcholinesterase Inhibitors in the Treatment of Alzheimer's Disease.* J Cent Nerv Syst Dis, 2021. **13**: p. 11795735211029113.

26. Austin, P.C., *An Introduction to Propensity Score Methods for Reducing the Effects of Confounding in Observational Studies.* Multivariate Behav Res, 2011. **46**(3): p. 399-424.

27. R Core Team, *R: A language and environment for statistical computing. R Foundation for Statistical Computing, Vienna, Austria. URL* https://www.R-project.org/. 2022.

TauRx / Cytel Confidential: This is a controlled document and shall not be changed, saved, copied or reproduced by any unauthorized person.
